# Study of the interaction of 2*H*-furo[3,2-*b*]pyran-2-ones with nitrogen-containing nucleophiles

**DOI:** 10.3762/bjoc.21.44

**Published:** 2025-03-13

**Authors:** Constantine V Milyutin, Andrey N Komogortsev, Boris V Lichitsky

**Affiliations:** 1 N.D. Zelinsky Institute of Organic Chemistry, Russian Academy of Sciences, Leninsky Pr., 47, Moscow, 119991, Russian Federation

**Keywords:** allomaltol, enamines, 2*H*-furo[3,2-*b*]pyran-2-ones, pyrazol-3-ones, recyclization

## Abstract

For the first time, the reaction of substituted 2*H*-furo[3,2-*b*]pyran-2-ones with diverse *N*-nucleophiles was investigated. It was shown that the direction of the process depends on the type of employed nitrogen-containing reagent. For example, condensation with aliphatic amines leads to 2*H*-furo[3,2-*b*]pyran-2,7(3*H*)-diones bearing an exocyclic enamine moiety. At the same time, interaction with dinucleophiles results in recyclization accompanied by opening of the furan ring. Relied on the aforementioned process a general method for the synthesis of substituted pyrazol-3-ones with allomaltol fragment was designed. Structures of representatives of all obtained products were unambiguously confirmed by X-ray diffraction.

## Introduction

**Scheme 1:**
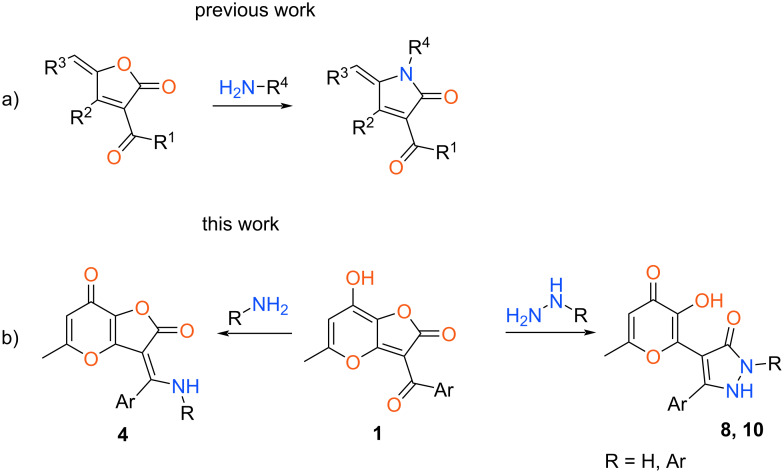
Various examples of transformations of furanones.

Substituted furan-2(5*H*)-ones (butenolides) are widely used as precursors for the preparation of diverse types of heterocyclic compounds possessing various biological activity [1–3]. Among the numerous approaches using considered furanones as starting compounds the recyclization processes are of significant interest [4–5]. The important subclass of such synthetic methods is the interaction with nitrogen-containing reagents. In this case depending on the structure of used *N*-nucleophile various types of a heterocyclic system can be obtained. A large number of examples of reactions with substituted amines have been reported in the literature. Generally, this process includes opening of the furanone ring followed by cyclization to the pyrrolone moiety [6–9]. Wherein, the wide variety of easily available starting butenolides and amines allows one to create a huge array of practically useful products. At the same time, the application of hydrazine derivatives expands the range of formed heterocycles. So, along with the aforementioned *N*-substituted pyrrolones such interaction can lead to pyridazinone systems [10–11]. Despite on the plenty of reactions with nitrogen-containing nucleophiles there is only one example of recyclization using furanone with a carbonyl group at position 3 (Scheme 1a, previous work) [6].

However, no work on this type of process with hydrazine derivatives is known in the literature. Therefore, the study of recyclizations of heterocyclic systems containing the 3-acylfuran-2(5*H*)-one core under action of various hydrazines is of a great interest.

Ongoing our research in the field of allomaltol chemistry in the present communication we investigated the interaction of substituted 2*H*-furo[3,2-*b*]pyran-2-ones **1** with nitrogen-containing nucleophiles (Scheme 1b, this work). As a result, the general approach to preparation of pyrazolones with a 3-hydroxy-4-pyranone unit was developed. It’s important to underline that both pyrazolone and allomaltol derivatives have the wide variety of biological activity [12–16]. This means that designing such hybrid products can significantly alter the pharmacological properties.

## Results and Discussion

**Scheme 2:**
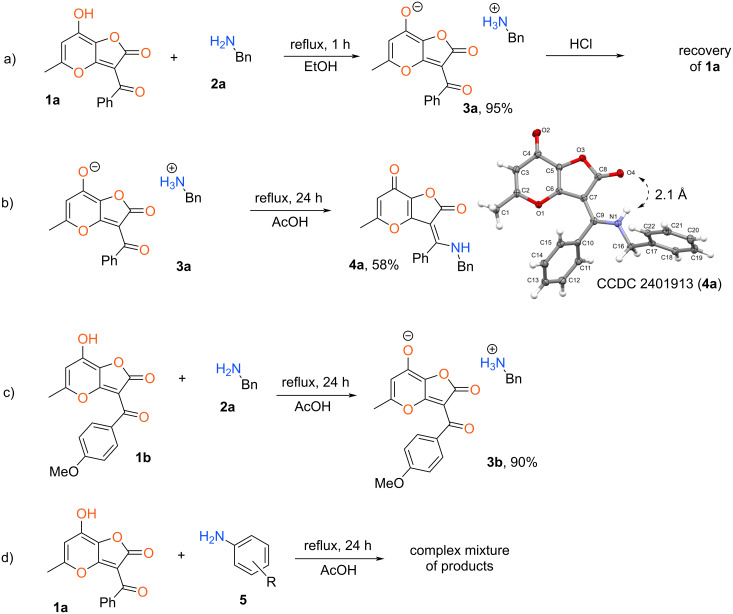
Interaction of starting 2*H*-furo[3,2-*b*]pyran-2-ones with diverse amines.

The starting compounds **1** were prepared from allomaltol employing a previously elaborated two-step method [17]. Relied on the obtained set of aroyl-substituted 2*H*-furo[3,2-*b*]pyran-2-ones **1** we investigated the interaction with diverse *N*-nucleophiles. Compound **1a** was selected as a model object for realization of considered research. At first, reaction with benzylamine (**2a**) was tested under various conditions. Initially, we performed the studied process with equivalent amounts of starting materials in ethanol at reflux for 1 h. As a result, no products of condensation or recyclization have been obtained. At the same time, stable salt **3a** was isolated in 95% yield (Scheme 2a). Besides that, the use of 3-fold excess of amine **2a** led to the same results. Apparently, the stability of salt **3a** is connected with high acidity of the starting furanone **1a** and its recovery is only possible under the action of strong acids (HCl, H_2_SO_4_). Further, we investigated the chemical behavior of synthesized salt **3a** at reflux in AcOH for 5 h. In this case the mixture of starting compound **3a** and an unidentified product was obtained. Next, we increased the reaction time up to 24 h what allowed us to achieve the complete conversion of salt **3a**. Wherein, the product **4a** was isolated in pure form with 58% yield. Based on X-ray analysis data the synthesized compound **4a** is 2*H*-furo[3,2-*b*]pyran-2,7(3*H*)-dione bearing an enamine fragment at the furanone ring (Scheme 2b). It’s interesting to note that prepared product **4a** exists in form of the *Z*-isomer. This configuration is apparently stabilized by an intramolecular hydrogen bond between the NH-unit and the carbonyl group.

The presented results indicate that the reaction of the aliphatic amine doesn’t lead to recyclization of the studied heterocyclic system. Further, we hypothesized that synthesis of the enamine **4a** can be realized in one-pot variant without isolation of intermediate salt **3a**. Indeed, the reflux of starting compound **1a** with amine **2a** in AcOH for 24 h led to formation of product **4a** with 62% yield.

**Scheme 3:**
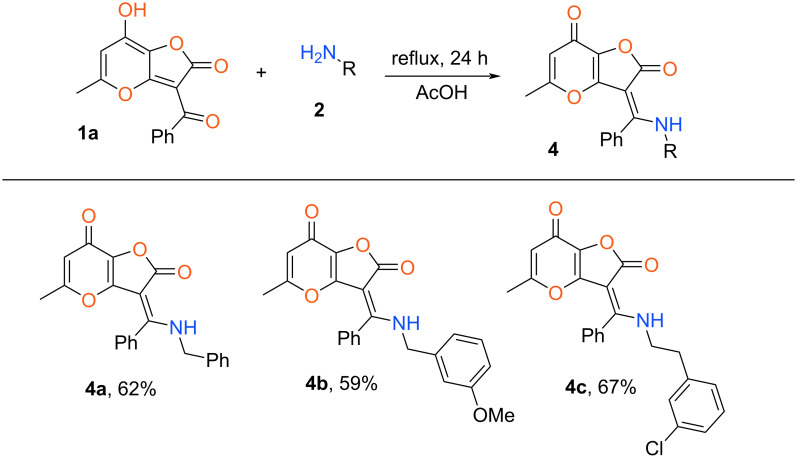
Synthesis of enamines **4**. Reaction conditions: **1a** (1 mmol, 0.38 g), amine **2** (1.2 mmol), AcOH (3 mL).

Using various furanones **1** and amines we attempted to synthesize analogues of compound **4a** based on the above procedure. It was found that the presence of an electron-donating group in the aroyl fragment deactivates the carbonyl moiety and as a consequence blocks the considered process. In this case only stable salt **3b** was obtained at reflux in AcOH for 24 h with 90% yield (Scheme 2c). At the same time, various aliphatic amines can be applied in the considered transformation using 2*H*-furo[3,2-*b*]pyran-2-ones **1** without electron-donating substituents at the aromatic ring. Also, the considered protocol failed for interactions of furanone **1a** with diverse anilines. In this case a complex mixture of products was obtained after 24 h reflux in AcOH (Scheme 2d). Apparently, for the realization of the presented condensation, the nucleophilicity of the aromatic amines is not sufficient. Wherein, the type of substituent in the aroyl fragment of furanone **1** doesn’t influence the result of this reaction. Thus, enamines **4** can be synthesized only using active aliphatic amines and 2*H*-furo[3,2-*b*]pyran-2-ones **1** without electron-donating units at the aryl fragment (Scheme 3).

Further, we investigated the interaction of furanones **1** with various hydrazine derivatives **6**. For this purpose, we tested the model reaction of furanone **1a** with phenylhydrazine **6a** under various conditions and the obtained results are summarized in Table 1. It should be mentioned that all experiments were carried out at reflux due to low solubility of the starting compound **1a** in all used solvents.

**Table 1:** Optimization of the reaction conditions^a^.

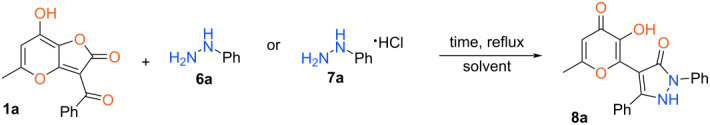				

Entry	Solvent	Reactant	Time, h	Yield, %

1	AcOH	**6a**	24	37
2	AcOH	**6a**	48	36
3	EtOH	**6a**	24	–
4	MeCN	**6a**	24	–
5	dioxane	**6a**	24	–
6	AcOH	**7a**	24	55
7	EtOH	**7a**	24	75
8	MeCN	**7a**	24	51
9	dioxane	**7a**	24	62
10	EtOH	**7a**	8	76
11	EtOH	**7a**	4	53

^a^Reaction conditions: **1a** (1 mmol, 0.38 g), reagent (1.1 mmol), solvent (5 mL).

At first, we carried out the process under conditions developed above for the synthesis of enamines **4**. So, reflux of the mixture of starting materials in AcOH for 24 h led to pyrazolone **8a** in 37% yield (Table 1, entry 1). Note that increasing the process time didn’t affect on the observed result (Table 1, entry 2). Next, we tested the studied reaction applying various solvents and in all cases the target product was not obtained (Table 1, entries 3–5). Apparently, the presence of acid reagent is necessary for implementation of considered recyclization. In this regard we tried to perform the process under study using phenylhydrazine in salt form. Indeed, reflux of furanone **1a** with the corresponding hydrochloride **7a** in AcOH for 24 h allows us to increase the yield of product **8a** up to 55% (Table 1, entry 6). Further, we varied the solvents for reaction with phenylhydrazine hydrochloride (Table 1, entries 7–9). All used solvents are suitable for the considered transformation while among the tested conditions the best result was achieved in the case of EtOH (Table 1, entry 7). Then, we optimized the process time for the reaction with reagent **7a**. It was shown that 8 h reflux is enough for the considered recyclization (Table 1, entry 10). Wherein, further shortening of the duration decreased the yield of product **8a** (Table 1, entry 11). Thus, the optimal conditions for studied process are the application of phenylhydrazine hydrochloride at reflux in EtOH for 8 h.

**Scheme 4:**
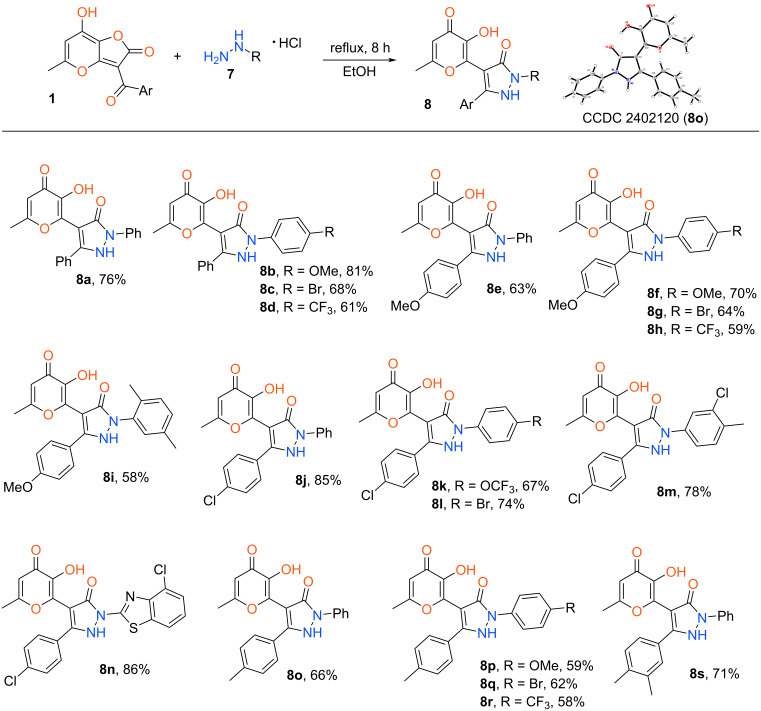
Synthesis of pyrazol-3-ones **8**. Reaction conditions: **1** (1 mmol), hydrazine **7** (1.1 mmol), EtOH (5 mL).

Having in hands the protocol elaborated above we have synthesized the wide range of target pyrazolones **8** bearing the allomaltol fragment (Scheme 4). The suggested method allows one to utilize arylhydrazines both with donor and acceptor substituents in the aromatic ring. Besides that, heterocyclic hydrazines also can be used in the considered transformation.

**Scheme 5:**
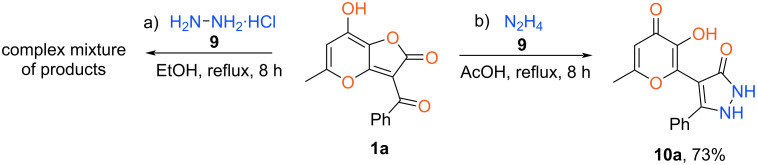
Synthesis of pyrazol-3-one **10a**.

In addition, we have tried to carry out the process under investigation with unsubstituted hydrazine. So, reflux of furanone **1a** with hydrazine monohydrochloride in EtOH for 8 h resulted in a complex mixture of products (Scheme 5a). Taking into account the fact that the nucleophilicity of hydrazine is higher than for arylhydrazines and similar to alkylamines we tested the conditions elaborated above for the preparation of enamines **4**. Interaction of starting compound **1a** with hydrazine was performed using acetic acid as a solvent at reflux for 8 h. As a result, the appropriate pyrazolone **10a**, unsubstituted at both nitrogen atoms, was obtained with 73% yield (Scheme 5b).

**Scheme 6:**
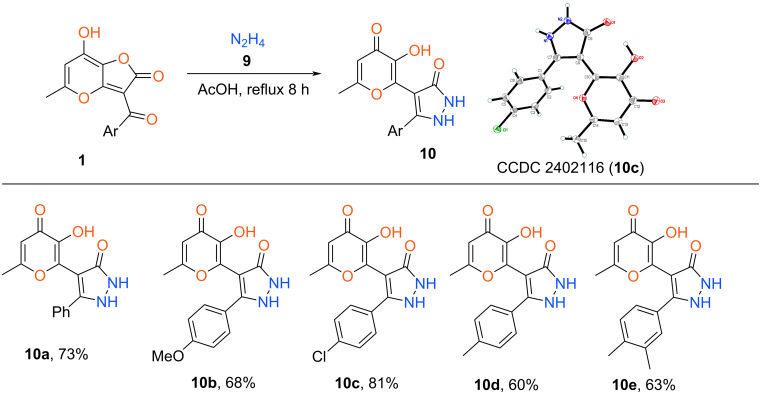
Synthesis of unsubstituted pyrazol-3-ones **10**. Reaction conditions: **1** (1 mmol), hydrazine hydrate (2 mmol, 0.10 g), AcOH (5 mL).

**Scheme 7:**
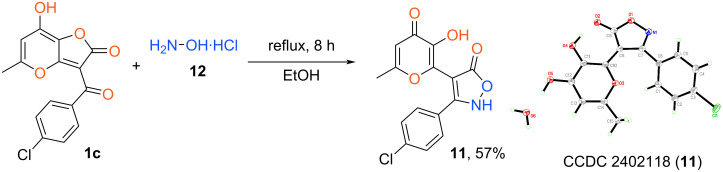
Synthesis of isoxazolone **11**. Reaction conditions: **1c** (1 mmol, 0.30 g), hydroxylamine hydrochloride (1.2 mmol, 0.08 g), EtOH (5 mL).

Based on the elaborated protocol we synthesized the set of target products **10** (Scheme 6). It has been shown that in contrast to the reaction with amines described above, this process does not depend on the type of substituent in the aromatic fragment. It is interesting to note that the presented approach (AcOH, reflux 8 h) failed in the case of aliphatic hydrazines (methylhydrazine, *tert*-butylhydrazine) leading to a complex mixture of products. Besides that, the use of hydrochlorides of aforementioned alkylhydrazines and EtOH as a solvent gave analogous negative results. At the same time, the disclosed recyclization can be extended for the synthesis of relative isoxazolone **11**. In this case the reaction of furanone **1c** with hydroxylamine hydrochloride **12** also was carried out in refluxing EtOH for 8 h (Scheme 7).

All prepared products **8**, **10** and **11** are solid crystalline compounds whose structure was proved by ^1^H, ^13^C NMR spectroscopy and high-resolution MS. ^1^H NMR spectra of obtained products contain characteristic signals of the protons of the methyl group in the region δ 1.72–2.16 ppm and proton of pyranone fragment in the region δ 5.98–6.62 ppm. Besides that, the key structures of synthesized products were established by X-ray analysis.

**Scheme 8:**
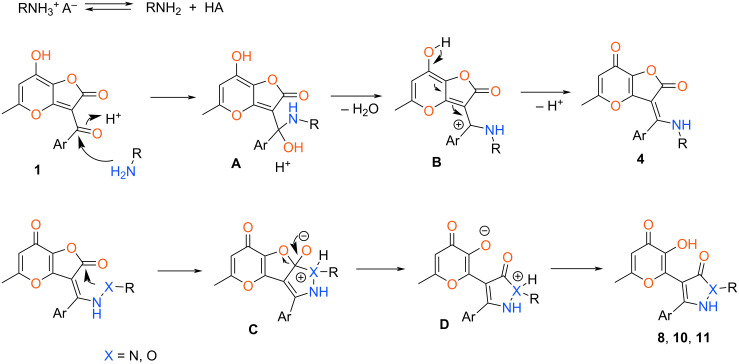
Proposed reaction mechanism.

The proposed mechanism of the considered processes is outlined at Scheme 8. Initially, the free nitrogen nucleophile is reversibly generated from the corresponding hydrochloride or acetate. Next, acid-catalyzed addition of the amine component to the carbon atom of the aroyl fragment leads to hemiaminal **A**. Then, enamine **4** is formed via dehydration of intermediate **B**. In the case of amines **2** the reaction stops at this stage while for other substrates the further recyclization proceeds. So, the additional NH or OH fragment attacks the lactone moiety leading to intermediate **C**. The subsequent opening of the furanone ring and proton transfer results in final compounds **8**, **10** and **11**.

**Scheme 9:**
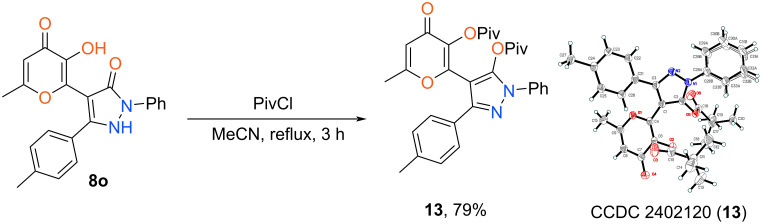
Synthesis of product **13**. Reaction conditions: **8o** (1 mmol, 0.37 g), pivaloyl chloride (3 mmol, 0.36 g), MeCN (5 mL).

The synthetic application of obtained pyrazolones **8** was demonstrated by its further derivatization. The interaction with electrophilic agents is determined by the presence of several nucleophilic centers in the molecule. In this regard we performed the acylation of starting compound **8o** using pivaloyl chloride. The process was carried out with 3-fold excess of the aforementioned reagent at reflux in MeCN for 3 h. As a result, product **13** bearing two acyl fragments at oxygen atoms of pyranone and pyrazole units was isolated (Scheme 9). The structure of synthesized compound **13** was unambiguously confirmed by X-ray diffraction.

## Conclusion

In summary, we studied the interaction of 2*H*-furo[3,2-*b*]pyran-2-one derivatives with various amines and hydrazines. It was demonstrated that, depending on the nature of the *N*-nucleophile used, two types of transformation are possible. So, 2*H*-furo[3,2-*b*]pyran-2,7(3*H*)-diones containing an exocyclic enamine unit are formed in the reaction with aliphatic amines. Wherein, condensation with hydrazines didn’t stop at the stage of enehydrazines and subsequent recyclization to pyrazolones occurred. The analogous process using hydroxylamine allowed us to prepare the similar isoxazolone with allomaltol fragment. Extensive studies have enabled the development of a straightforward approach to novel bifunctional products containing both allomaltol and pyrazolone cores. The structures of key synthesized products were unambiguously proved by X-ray diffraction.

## Supporting Information

File 1 General information, characterization data, NMR spectra and crystallographic data of synthesized compounds.

## Data Availability

All data that supports the findings of this study is available in the published article and/or the supporting information of this article.
