# Supplementary material for: Study of the interaction of 2H-furo[3,2-b]pyran-2-ones with nitrogen-containing nucleophiles
Source: Beilstein J Org Chem. 2025 Mar 13;21:556–63. doi: 10.3762/bjoc.21.44 (PMC11912642; doi:10.3762/bjoc.21.44)
Supplement: File 1 — General information, characterization data, NMR spectra and crystallographic data of synthesized compounds. [file Beilstein_J_Org_Chem-21-556-s001.pdf]

BEILSTEIN JOURNAL OF ORGANIC CHEMISTRY

Supporting Information

for

Study of the interaction of 2*H*-furo[3,2-*b*]pyran-2-ones with nitrogen-containing nucleophiles

Constantine V. Milyutin, Andrey N. Komogortsev and Boris V. Lichitsky

Beilstein J. Org. Chem. **2025,** *21,* 556–563. doi:10.3762/bjoc.21.44

General information, characterization data, NMR spectra and crystallographic data of synthesized compounds

License and Terms: This is a supporting information file under the terms of the Creative Commons Attribution License (https://creativecommons.org/licenses/by/4.0). Please note that the reuse, redistribution and reproduction in particular requires that the author(s) and source are credited and that individual graphics may be subject to special legal provisions.
The license is subject to the *Beilstein Journal of Organic Chemistry* terms and conditions: (https://www.beilstein-journals.org/bjoc/terms)

Table of contents

1. General information S2

2. Characterization data of compounds 3 .... S4

3. Characterization data of compounds 4 .... S5

4. Characterization data of compounds 8 .... S6

5. Characterization data of compounds 10 .... S10

6. Characterization data of compounds 11 and 13 .... S11

7. NMR ^{1}H and ^{13}C spectra for compounds 3 .... S12

8. NMR ^{1}H and ^{13}C spectra for compounds 4 .... S14

9. NMR ^{1}H and ^{13}C spectra for compounds 8 .... S17

10. NMR ^{1}H and ^{13}C spectra for compounds 10 .... S36

11. NMR ^{1}H and ^{13}C spectra for compounds 11 and 13 .... S41

12. X-ray crystallographic data and refinement details S43

12.1 Crystallographic data for *(Z)-3-((benzylamino)(phenyl)methylene)-5-methyl-2H-furo[3,2-b]pyran-2,7(3H)-dione* (**4a**) S44

12.2 Crystallographic data for *4-(3-hydroxy-6-methyl-4-oxo-4H-pyran-2-yl)-2-phenyl-5-(p-tolyl)-1,2-dihydro-3H-pyrazol-3-one* (**8o**) S54

12.3 Crystallographic data for *5-(4-chlorophenyl)-4-(3-hydroxy-6-methyl-4-oxo-4H-pyran-2-yl)-1,2-dihydro-3H-pyrazol-3-one* (**10c**) S64

12.4 Crystallographic data for *3-(4-chlorophenyl)-4-(3-hydroxy-6-methyl-4-oxo-4H-pyran-2-yl)isoxazol-5(2H)-one* (**11**) S72

12.5 Crystallographic data for *6-methyl-4-oxo-2-(1-phenyl-5-(pivaloyloxy)-3-(p-tolyl)-1H-pyrazol-4-yl)-4H-pyran-3-yl pivalate* (**13**) S80

1. General information

General information. Unless otherwise stated, all starting chemicals were commercially available and were used as received. The starting compounds **1** were prepared to a procedure described in the literature[1]. NMR spectra were recorded with Bruker AM 300 (300 MHz) in DMSO-d_6. Chemical shifts (ppm) are given relative to solvent signals (DMSO-d_6: 2.50 ppm (^{1}H NMR) and 39.52 ppm (^{13}C NMR). High-resolution mass spectra (HRMS) were obtained on a Bruker micrOTOF II instrument using electrospray ionization (ESI). The melting points were determined on a Kofler hot stage. Magnetic stirrer IKA C-MAG HS 7 was used for the reactions that require heating.

[1] Komogortsev, A.N.; Lichitsky, B.V; Milyutin, C.V; Melekhina, V.G. *J. Heterocycl. Chem.*, **2024**, *61*, 86–92.

Experimental procedure for the synthesis of salt 3a.

The mixture of 2*H*-furo[3,2-*b*]pyran-2-one **1a** (1 mmol, 0.27 g) and benzylamine (**2a**, 1.2 mmol, 0.13 g) in EtOH (3 mL) was refluxed for 1 h. Then the resulting solution was evaporated in vacuo and the residue was triturated with MeCN (3 mL). The obtained product was filtered off and washed with MeCN (3×5 mL).

Experimental procedure for the synthesis of product 4a.

The salt **3a** (1 mmol, 0.38 g) was refluxed for 24 h in AcOH (3 mL). Then the resulting solution was evaporated in vacuo and the residue was recrystallized from EtOH (3 mL). The obtained product was filtered off and washed with EtOH (3 × 5 mL).

General experimental procedure for the synthesis of enamines 4.

The mixture of 2*H*-furo[3,2-*b*]pyran-2-one **1a** (1 mmol, 0.27 g) and appropriate amine **2** (1.2 mmol) in AcOH (3 mL) was refluxed for 24 h. Then the resulting solution was evaporated in vacuo and the residue was recrystallized from EtOH (3 mL). The obtained product was filtered off and washed with EtOH (3 × 5 mL).

Experimental procedure for the synthesis of salt 3b.

The mixture of 2*H*-furo[3,2-*b*]pyran-2-one **1b** (1 mmol, 0.30 g) and benzylamine (**2a**, 1.2 mmol, 0.13 g) in AcOH (3 mL) was refluxed for 24 h. Then the resulting solution was evaporated in vacuo and the residue was triturated with MeCN (3 mL). The obtained product was filtered off and washed with MeCN (3 × 5 mL).

General experimental procedure for the synthesis of pyrazolones 8.

The mixture of corresponding 2*H*-furo[3,2-*b*]pyran-2-one **1** (1 mmol) and substituted hydrazine hydrochloride **7** (1.1 mmol) in EtOH (5 mL) was refluxed for 8 h. The obtained solution was cooled to room temperature and formed precipitate was filtered off and washed with EtOH (3 × 5 mL).

General experimental procedure for the synthesis of pyrazolones 10.

The mixture of corresponding 2*H*-furo[3,2-*b*]pyran-2-one **1** (1 mmol) and hydrazine hydrate **9** (2 mmol, 0.10 g) in AcOH (5 mL) was refluxed for 8 h. Then the resulting solution was evaporated in vacuo and the residue was recrystallized from EtOH (3 mL). The obtained product was filtered off and washed with EtOH (3×5 mL).

Experimental procedure for the synthesis of product 11.

The mixture of 2*H*-furo[3,2-*b*]pyran-2-one **1c** (1 mmol, 0.30 g) and hydroxylamine hydrochloride (1.2 mmol, 0.08 g) in EtOH (5 mL) was refluxed for 8 h. The obtained solution was cooled to room temperature and obtained precipitate was filtered off and washed with EtOH (3×5 mL).

Experimental procedure for the synthesis of product 13.

The mixture of compound **8o** (1 mmol, 0.37 g) and pivaloyl chloride (3 mmol, 0.36 g) in MeCN (5 mL) was refluxed for 3 h. Then the resulting solution was evaporated in vacuo and the residue was triturated with Et_2O (5 mL). The obtained product was filtered off and washed with Et_2O (3×5 mL).

2. Characterization data of compounds 3

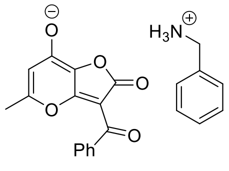

Phenylmethanaminium 3-benzoyl-5-methyl-2-oxo-2H-furo[3,2-b]pyran-7-olate salt (**3a**).

Yellow powder; yield 95% (0.36 g); mp 137-139°C. ^{1}H NMR (300 MHz, DMSO-d_6) δ 8.11 (s, 3H), 7.61 – 7.52 (m, 2H), 7.50 – 7.29 (m, 8H), 5.91 (s, 1H), 4.04 (s, 2H), 2.16 (s, 3H). ^{13}C NMR (75 MHz, DMSO-d_6) δ 186.10, 165.32, 161.44, 159.62, 157.09, 140.99, 134.03, 129.90, 128.89, 128.66, 128.52, 128.21, 127.26, 125.72, 113.16, 87.31, 42.47, 18.96. HRMS (ESI-TOF) *m/z*: Calcld for $C_{15}H_9O_5$ $[M\text{-}H]^-$ 269.0455; Found: 269.0462.

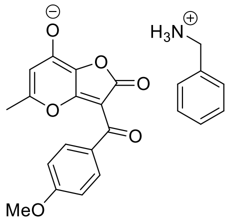

Phenylmethanaminium 3-(4-methoxybenzoyl)-5-methyl-2-oxo-2H-furo[3,2-b]pyran-7-olate salt (**3b**).

Yellow powder; yield 90% (0.37 g); mp 149-151°C. ^{1}H NMR (300 MHz, DMSO-d_6) δ 8.23 (s, 3H), 7.64 (d, *J* = 8.3 Hz, 2H), 7.51 – 7.36 (m, 5H), 6.91 (d, *J* = 8.3 Hz, 2H), 5.92 (s, 1H), 4.06 (s, 2H), 3.80 (s, 3H), 2.17 (s, 3H). ^{13}C NMR (75 MHz, DMSO-d_6) δ 185.11, 165.37, 161.12, 160.99, 159.40, 157.80, 134.01, 133.15, 130.47, 128.86, 128.64, 128.49, 125.50, 113.05, 112.47, 87.08, 55.20, 42.44, 18.98. HRMS (ESI-TOF) *m/z*: Calcld for $C_{16}H_{14}O_6$ $[M\text{-}H]^-$ 299.0561; Found: 299.0572.

3. Characterization data of compounds 4

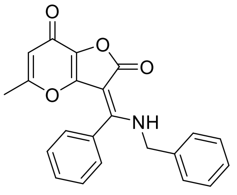

(Z)-3-((Benzylamino)(phenyl)methylene)-5-methyl-2H-furo[3,2-b]pyran-2,7(3H)-dione (**4a**).

Yellow powder; yield 62% (0.22 g); mp 213-215°C. ^{1}H NMR (300 MHz, DMSO-d_6) δ 10.19 (s, 1H), 7.68 – 7.45 (m, 5H), 7.39 – 7.27 (m, 3H), 7.24 – 7.15 (m, 2H), 6.01 (s, 1H), 4.54 (d, J = 4.6 Hz, 2H), 1.76 (s, 3H). ^{13}C NMR (75 MHz, DMSO-d_6) δ 165.46, 165.34, 164.19, 161.76, 151.62, 137.43, 131.29, 130.05, 129.58, 129.16, 129.08, 128.25, 128.17, 127.69, 114.37, 85.99, 48.38, 18.71. HRMS (ESI-TOF) *m/z*: Calcld for $C_{22}H_{16}NO_4$ [M+H]$^+$ 360.1230; Found: 360.1244.

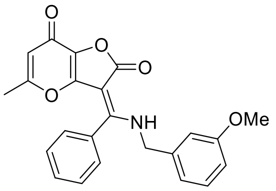

(Z)-3-(((3-Methoxybenzyl)amino)(phenyl)methylene)-5-methyl-2H-furo[3,2-b]pyran-2,7(3H)-dione (**4b**).

Yellow powder; yield 59% (0.23 g); mp 185-187°C. ^{1}H NMR (300 MHz, DMSO-d_6) δ 10.17 (s, 1H), 7.68 – 7.45 (m, 5H), 7.31 – 7.20 (m, 1H), 6.89 – 6.81 (m, 1H), 6.81 – 6.71 (m, 2H), 6.00 (s, 1H), 4.50 (d, J = 5.3 Hz, 2H), 3.71 (s, 3H), 1.76 (s, 3H). ^{13}C NMR (75 MHz, DMSO-d_6) δ 165.05, 164.97, 163.82, 161.39, 159.48, 151.24, 138.52, 130.91, 129.92, 129.60, 129.19, 128.67, 127.90, 119.35, 113.93, 113.24, 112.95, 85.61, 55.11, 47.91, 18.30. HRMS (ESI-TOF) *m/z*: Calcld for $C_{23}H_{20}NO_5$ [M+H]$^+$ 390.1336; Found: 390.1335.

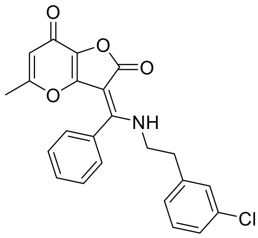

(Z)-3-(((3-Chlorophenethyl)amino)(phenyl)methylene)-5-methyl-2H-furo[3,2-b]pyran-2,7(3H)-dione (**4c**).

Pale yellow powder; yield 67% (0.27 g); mp 173-175°C. ^{1}H NMR (300 MHz, DMSO-d_6) δ 9.90 (t, J = 6.4 Hz, 1H), 7.64 – 7.41 (m, 3H), 7.33 – 7.19 (m, 4H), 7.13 (s, 1H), 7.07 – 6.99 (m, 1H), 5.98 (s, 1H), 3.57 – 3.47 (m, 2H), 2.87 (t, J = 6.8 Hz, 2H), 1.72 (s, 3H). ^{13}C NMR (75 MHz, DMSO-d_6) δ 165.01, 164.79, 163.69, 161.28, 151.16, 140.41, 133.20, 130.71, 130.38, 129.60, 128.95, 128.80, 128.54, 127.67, 127.57, 126.65, 113.86, 84.95, 46.13, 35.17, 18.24. HRMS (ESI-TOF) *m/z*: Calcld for $C_{23}H_{19}ClNO_4$ [M+H]$^+$ 408.0997; Found: 408.0997.

4. Characterization data of compounds 8

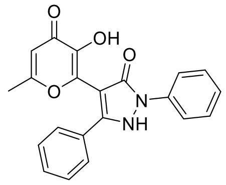

4-(3-Hydroxy-6-methyl-4-oxo-4H-pyran-2-yl)-2,5-diphenyl-1,2-dihydro-3H-pyrazol-3-one (**8a**)

Pale yellow powder; yield 76% (0.27 g); mp 210-212°C. ^{1}H NMR (300 MHz, DMSO-d_6) δ 7.88 (d, J = 8.0 Hz, 2H), 7.59 – 7.45 (m, 4H), 7.44 – 7.35 (m, 3H), 7.34 – 7.26 (m, 1H), 6.43 (s, 1H), 2.07 (s, 3H). ^{13}C NMR (75 MHz, DMSO-d_6) δ 170.05, 164.14, 156.02, 148.36, 146.62, 140.84, 138.42, 133.51, 128.93, 128.16, 127.28, 125.83, 120.99, 109.72, 93.40, 18.94. HRMS (ESI-TOF) *m/z*: Calcld for $C_{21}H_{17}N_2O_4$ $[M+H]^+$ 361.1183; Found: 361.1194.

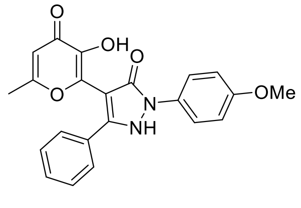

4-(3-Hydroxy-6-methyl-4-oxo-4H-pyran-2-yl)-2-(4-methoxyphenyl)-5-phenyl-1,2-dihydro-3H-pyrazol-3-one (**8b**).

Brown powder; yield 81% (0.32 g); mp 183-185°C. ^{1}H NMR (300 MHz, DMSO-d_6) δ 7.73 (d, J = 8.5 Hz, 2H), 7.54 (d, J = 7.0 Hz, 2H), 7.45 – 7.32 (m, 3H), 7.06 (d, J = 8.5 Hz, 2H), 6.39 (s, 1H), 3.80 (s, 3H), 2.08 (s, 3H). ^{13}C NMR (75 MHz, DMSO-d_6) δ 170.78, 164.19, 157.44, 154.84, 147.74, 141.34, 133.48, 131.45, 128.17, 128.08, 127.13, 123.08, 114.08, 110.00, 93.01, 55.36, 18.99. HRMS (ESI-TOF) *m/z*: Calcld for $C_{22}H_{19}N_2O_4$ $[M+H]^+$ 391.1288; Found: 391.1290.

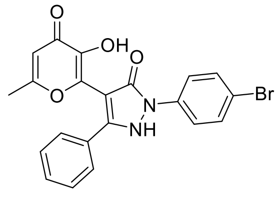

2-(4-Bromophenyl)-4-(3-hydroxy-6-methyl-4-oxo-4H-pyran-2-yl)-5-phenyl-1,2-dihydro-3H-pyrazol-3-one (**8c**).

Yellow powder; yield 68% (0.30 g); mp 201-203°C. ^{1}H NMR (300 MHz, DMSO-d_6) δ 7.91 (d, J = 8.8 Hz, 2H), 7.66 (d, J = 8.8 Hz, 2H), 7.59 – 7.49 (m, 2H), 7.45 – 7.34 (m, 3H), 6.48 (s, 1H), 2.03 (s, 3H). ^{13}C NMR (75 MHz, DMSO-d_6) δ 168.84, 164.08, 157.46, 148.93, 148.50, 139.94, 137.92, 133.59, 131.80, 128.27, 128.12, 127.53, 122.23, 117.75, 109.25, 93.75, 18.85. HRMS (ESI-TOF) *m/z*: Calcld for $C_{21}H_{16}BrN_2O_4$ $[M+H]^+$ 439.0288; Found: 439.0298.

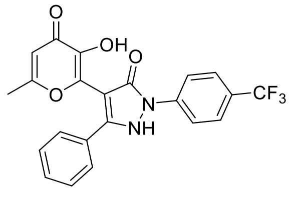

4-(3-Hydroxy-6-methyl-4-oxo-4H-pyran-2-yl)-5-phenyl-2-(4-(trifluoromethyl)phenyl)-1,2-dihydro-3H-pyrazol-3-one (**8d**).

Yellow powder; yield 61% (0.26 g); mp 220-222°C.^{1}H NMR (300 MHz, DMSO-d_6) δ 8.23 (d, J = 8.4 Hz, 2H), 7.83 (d, J = 8.5 Hz, 2H), 7.59 – 7.50 (m, 2H), 7.47 – 7.37 (m, 3H), 6.51 (s, 1H), 1.99 (s, 3H).^{13}C NMR (75 MHz, DMSO-d_6) δ 168.54, 161.63 (d, J_{CF} = 314.0 Hz), 149.77 (d, J_{CF} = 26.0 Hz), 142.11, 138.92, 133.87, 128.25, 128.01, 127.82, 126.14, 119.62, 109.07, 93.91, 18.70. HRMS (ESI-TOF) *m/z*: Calcld for $C_{22}H_{16}F_3N_2O_4$ $[M+H]^+$ 429.1057; Found: 429.1060.

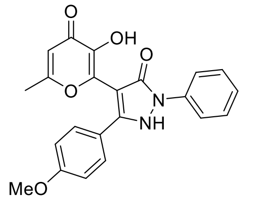

4-(3-Hydroxy-6-methyl-4-oxo-4H-pyran-2-yl)-5-(4-methoxyphenyl)-2-phenyl-1,2-dihydro-3H-pyrazol-3-one (**8e**).

Pale yellow powder; yield 63% (0.25 g); mp 212-214°C. ^{1}H NMR (300 MHz, DMSO-d_6) δ 7.87 (d, J = 8.0 Hz, 2H), 7.55 – 7.43 (m, 4H), 7.29 (t, J = 7.3 Hz, 1H), 6.97 (d, J = 8.4 Hz, 2H), 6.42 (s, 1H), 3.78 (s, 3H), 2.11 (s, 3H). ^{13}C NMR (75 MHz, DMSO-d_6) δ 170.52, 164.27, 159.42, 155.96, 148.21, 146.14, 141.19, 138.34, 128.96, 128.60, 125.80, 125.59, 121.00, 113.67, 109.93, 93.28, 55.17, 19.11. HRMS (ESI-TOF) *m/z*: Calcld for $C_{22}H_{19}N_2O_5$ $[M+H]^+$ 391.1288; Found: 391.1299.

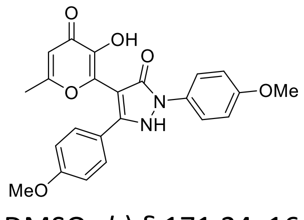

4-(3-Hydroxy-6-methyl-4-oxo-4H-pyran-2-yl)-2,5-bis(4-methoxyphenyl)-1,2-dihydro-3H-pyrazol-3-one (**8f**).

Dark yellow powder; yield 70% (0.29 g); mp 220-222°C. ^{1}H NMR (300 MHz, DMSO-d_6) δ 7.71 (d, J = 8.7 Hz, 2H), 7.47 (d, J = 8.4 Hz, 2H), 7.05 (d, J = 8.7 Hz, 2H), 6.96 (d, J = 8.4 Hz, 2H), 6.37 (s, 1H), 3.80 (s, 3H), 3.77 (s, 3H), 2.11 (s, 3H). ^{13}C NMR (75 MHz, DMSO-d_6) δ 171.24, 164.29, 159.35, 157.43, 154.84, 147.55, 145.15, 141.62, 131.33, 128.46, 125.56, 123.13, 114.11, 113.68, 110.21, 92.87, 55.39, 55.17, 19.14. HRMS (ESI-TOF) *m/z*: Calcld for $C_{23}H_{21}N_2O_6$ $[M+H]^+$ 421.1394; Found: 421.1391.

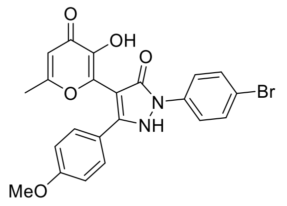

2-(4-Bromophenyl)-4-(3-hydroxy-6-methyl-4-oxo-4H-pyran-2-yl)-5-(4-methoxyphenyl)-1,2-dihydro-3H-pyrazol-3-one (**8g**).

Pale brown powder; yield 64% (0.30 g); mp 249-254°C. ^{1}H NMR (300 MHz, DMSO-d_6) δ 7.89 (d, J = 8.1 Hz, 2H), 7.66 (d, J = 8.1 Hz, 2H), 7.48 (d, J = 8.0 Hz, 2H), 6.96 (d, J = 8.1 Hz, 2H), 6.46 (s, 1H), 3.78 (s, 3H), 2.09 (s, 3H). ^{13}C NMR (75 MHz, DMSO-d_6) δ 169.43, 164.20, 159.45, 157.19, 148.71, 147.76, 140.37, 137.85, 131.81, 128.79, 125.68, 122.22, 117.68, 113.61, 109.50, 93.52, 55.18, 19.03. HRMS (ESI-TOF) *m/z*: Calcld for $C_{22}H_{18}BrN_2O_5$ [M+H]$^+$ 469.0394; Found: 469.0405.

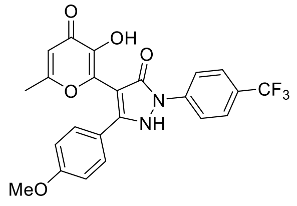

4-(3-Hydroxy-6-methyl-4-oxo-4H-pyran-2-yl)-5-(4-methoxyphenyl)-2-(4-(trifluoromethyl)phenyl)-1,2-dihydro-3H-pyrazol-3-one (**8h**).

Pale yellow powder; yield 59% (0.27 g); mp 180-182°C. ^{1}H NMR (300 MHz, DMSO-d_6) δ 8.20 (d, J = 9.0 Hz, 2H), 7.83 (d, J = 8.7 Hz, 2H), 7.54 – 7.44 (m, 2H), 7.03 – 6.92 (m, 2H), 6.55 – 6.49 (m, 1H), 3.78 (s, 3H), 2.07 (s, 3H). ^{13}C NMR (76 MHz, DMSO) δ 168.55, 164.08, 159.53, 149.44, 141.92, 139.53, 129.04, 126.20 (d, J_{CF} = 3.7 Hz), 125.80, 119.79, 113.56, 109.11, 93.87, 55.19, 18.96. HRMS (ESI-TOF) *m/z*: Calcld for $C_{23}H_{18}FN_2O_5$ [M+H]$^+$ 459.1162; Found: 459.1163.

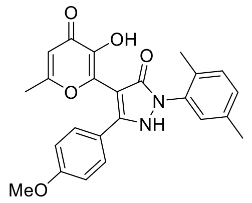

2-(2,5-Dimethylphenyl)-4-(3-hydroxy-6-methyl-4-oxo-4H-pyran-2-yl)-5-(4-methoxyphenyl)-1,2-dihydro-3H-pyrazol-3-one (**8i**).

White powder; yield 58% (0.24 g); mp 164-166°C. ^{1}H NMR (300 MHz, DMSO-d_6) δ 7.45 (d, J = 8.3 Hz, 2H), 7.32 – 7.16 (m, 3H), 6.95 (d, J = 8.3 Hz, 2H), 6.31 (s, 1H), 3.77 (s, 3H), 2.33 (s, 3H), 2.16 (s, 3H), 2.10 (s, 3H). ^{13}C NMR (75 MHz, DMSO-d_6) δ 172.26, 164.22, 159.41, 147.38, 143.93, 141.99, 136.03, 135.87, 132.06, 130.57, 129.49, 128.41, 128.11, 113.70, 110.66, 55.17, 20.29, 19.16, 17.17. HRMS (ESI-TOF) *m/z*: Calcld for $C_{24}H_{23}N_2O_5$ [M+H]$^+$ 419.1601; Found: 419.1600.

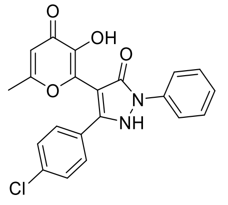

5-(4-Chlorophenyl)-4-(3-hydroxy-6-methyl-4-oxo-4H-pyran-2-yl)-2-phenyl-1,2-dihydro-3H-pyrazol-3-one (**8j**).

Pale yellow powder; yield 85% (0.33 g); mp 233-235°C. ^{1}H NMR (300 MHz, DMSO-d_6) δ 7.92 – 7.83 (m, 2H), 7.62 – 7.42 (m, 6H), 7.36 – 7.25 (m, 1H), 6.45 (s, 1H), 2.12 (s, 3H). ^{13}C NMR (126 MHz, DMSO-d_6) δ 170.27, 164.44, 155.83, 147.14, 146.31, 141.00, 138.41, 132.86, 132.51, 129.02, 128.98, 128.33, 126.04, 121.17, 109.85, 93.22, 19.10. HRMS (ESI-TOF) *m/z*: Calcld for $C_{21}H_{16}ClN_2O_4$ [M+H]$^+$ 395.0793; Found: 395.0788.

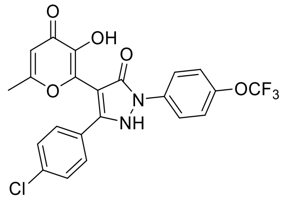

5-(4-Chlorophenyl)-4-(3-hydroxy-6-methyl-4-oxo-4H-pyran-2-yl)-2-(4-(trifluoromethoxy)phenyl)-1,2-dihydro-3H-pyrazol-3-one (**8k**).

Yellow powder; yield 67% (0.32 g); mp 238-240°C. ^{1}H NMR (300 MHz, DMSO-d_6) δ 8.08 – 7.99 (m, 2H), 7.62 – 7.53 (m, 1H), 7.52 – 7.44 (m, 5H), 6.50 (s, 1H), 2.09 (s, 3H). ^{13}C NMR (75 MHz, DMSO-d_6) δ 169.12, 164.35, 157.46, 148.29, 147.76, 145.59, 140.01, 137.59, 133.01, 132.56, 129.31, 128.27, 122.18, 121.78, 109.39, 93.55 18.97. HRMS (ESI-TOF) *m/z*: Calcld for $C_{22}H_{15}ClF_3N_2O_5$ [M+H]$^+$ 479.0616; Found: 479.0617.

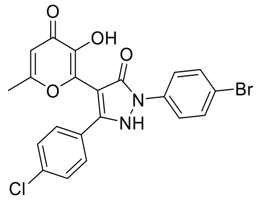

2-(4-Bromophenyl)-5-(4-chlorophenyl)-4-(3-hydroxy-6-methyl-4-oxo-4H-pyran-2-yl)-1,2-dihydro-3H-pyrazol-3-one (**8l**).

Pale yellow powder; yield 74% (0.35 g); mp 245-247°C. ^{1}H NMR (300 MHz, DMSO-d_6) δ 7.91 (d, J = 8.7 Hz, 2H), 7.65 (d, J = 8.8 Hz, 2H), 7.56 (d, J = 8.4 Hz, 2H), 7.46 (d, J = 8.3 Hz, 2H), 6.47 (s, 1H), 2.07 (s, 3H). ^{13}C NMR (126 MHz, DMSO-d_6) δ 169.26, 164.17, 157.58, 148.21, 147.61, 139.94, 137.98, 132.92, 132.63, 131.85, 129.31, 128.23, 122.25, 117.82, 109.47, 93.54, 18.97. HRMS (ESI-TOF) *m/z*: Calcld for $C_{21}H_{15}BrClN_2O_4$ [M+H]$^+$ 472.9898; Found: 472.9898.

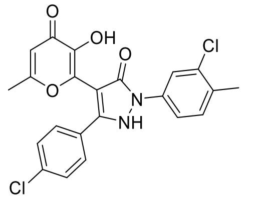

2-(3-Chloro-4-methylphenyl)-5-(4-chlorophenyl)-4-(3-hydroxy-6-methyl-4-oxo-4H-pyran-2-yl)-1,2-dihydro-3H-pyrazol-3-one (**8m**).

Yellow powder; yield 78% (0.34 g); mp 245-247°C. ^{1}H NMR (300 MHz, DMSO-d_6) δ 8.02 – 7.95 (m, 1H), 7.86 – 7.77 (m, 1H), 7.56 (d, *J* = 8.4 Hz, 2H), 7.50 – 7.39 (m, 3H), 6.52 (s, 1H), 2.34 (s, 3H), 2.07 (s, 3H). ^{13}C NMR (75 MHz, DMSO-d_6) δ 168.92, 164.08, 157.83, 148.72, 147.55, 139.64, 137.68, 133.25, 132.90, 132.63, 132.33, 131.42, 129.38, 128.15, 120.09, 118.74, 109.22, 93.64, 19.08, 18.90. HRMS (ESI-TOF) *m/z*: Calcld for $C_{21}H_{15}BrClN_2O_4$ [M+H]$^+$ 443.0560; Found: 443.0557.

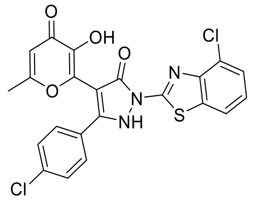

2-(4-Chlorobenzo[d]thiazol-2-yl)-5-(4-chlorophenyl)-4-(3-hydroxy-6-methyl-4-oxo-4H-pyran-2-yl)-1,2-dihydro-3H-pyrazol-3-one (**8n**).

Yellow powder; yield 86% (0.42 g); mp 300+°C. ^{1}H NMR (300 MHz, DMSO-d_6) δ 8.69 (br. s, 1H), 7.98 (d, *J* = 7.9 Hz, 1H), 7.61 (d, *J* = 8.1 Hz, 2H), 7.56 – 7.44 (m, 3H), 7.36 – 7.25 (m, 1H), 6.62 (s, 1H), 1.96 (s, 3H). ^{13}C NMR (75 MHz, DMSO-d_6) δ 165.97, 164.30, 162.27, 155.51, 153.40, 151.48, 146.07, 136.63, 133.95, 133.56, 132.10, 130.38, 127.96, 126.38, 125.13, 124.84, 120.85, 108.12, 93.10, 18.58. HRMS (ESI-TOF) *m/z*: Calcld for $C_{22}H_{14}Cl_2N_3O_4S$ [M+H]$^+$ 486.0077; Found: 486.0085.

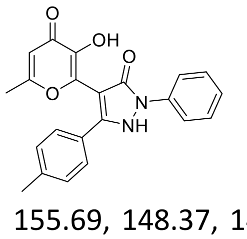

4-(3-Hydroxy-6-methyl-4-oxo-4H-pyran-2-yl)-2-phenyl-5-(p-tolyl)-1,2-dihydro-3H-pyrazol-3-one (**8o**).

Pale yellow powder; yield 66% (0.25 g); mp 214-216°C. ^{1}H NMR (300 MHz, DMSO-d_6) δ 7.87 (d, *J* = 8.0 Hz, 2H), 7.54 – 7.40 (m, 4H), 7.30 (t, *J* = 7.4 Hz, 1H), 7.21 (d, *J* = 7.8 Hz, 2H), 6.41 (s, 1H), 2.32 (s, 3H), 2.10 (s, 3H). ^{13}C NMR (75 MHz, DMSO-d_6) δ 170.45, 164.25, 155.69, 148.37, 146.05, 143.17, 141.17, 138.36, 137.61, 130.49, 128.75, 127.11, 125.84, 121.05, 109.86, 93.28, 20.86, 19.03. HRMS (ESI-TOF) *m/z*: Calcld for $C_{22}H_{19}N_2O_4$ [M+H]$^+$ 375.1339; Found: 375.1335.

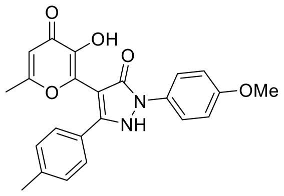

4-(3-Hydroxy-6-methyl-4-oxo-4H-pyran-2-yl)-2-(4-methoxyphenyl)-5-(p-tolyl)-1,2-dihydro-3H-pyrazol-3-one (**8p**).

Orange powder; yield 59% (0.24 g); mp 217-219°C. ^{1}H NMR (300 MHz, DMSO-d_6) δ 7.73 (d, *J* = 8.8 Hz, 2H), 7.42 (d, *J* = 7.8 Hz, 2H), 7.19 (d, *J* = 7.8 Hz, 2H), 7.05 (d, *J* = 8.7 Hz, 2H), 6.36 (s, 1H), 3.80 (s, 3H), 2.32 (s, 3H), 2.09 (s, 3H). ^{13}C NMR (75 MHz, DMSO-d_6) δ 171.28, 164.10, 157.35, 154.92, 147.69, 145.25, 141.41, 137.43, 131.50, 130.59, 128.72, 127.03, 123.00, 114.06, 110.18, 92.85, 55.36, 20.85, 19.04. HRMS (ESI-TOF) *m/z*: Calcld for $C_{23}H_{21}N_2O_5$ [M+H]$^+$ 405.1445; Found: 405.1440.

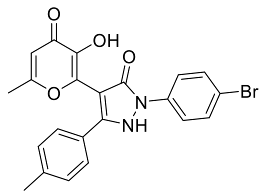

2-(4-Bromophenyl)-4-(3-hydroxy-6-methyl-4-oxo-4H-pyran-2-yl)-5-(p-tolyl)-1,2-dihydro-3H-pyrazol-3-one (**8q**).

Pale yellow powder; yield 62% (0.28 g); mp 191-193°C. ^{1}H NMR (300 MHz, DMSO-d_6) δ 7.89 (d, *J* = 8.8 Hz, 2H), 7.66 (d, *J* = 8.8 Hz, 2H), 7.43 (d, *J* = 7.8 Hz, 2H), 7.21 (d, *J* = 7.8 Hz, 2H), 6.46 (s, 1H), 2.32 (s, 3H), 2.07 (s, 3H). ^{13}C NMR (75 MHz, DMSO-d_6) δ 169.44, 164.21, 157.01, 148.90, 147.68, 140.38, 137.88, 137.73, 131.83, 130.57, 128.74, 127.34, 122.30, 117.78, 109.49, 93.56, 20.92, 18.98. HRMS (ESI-TOF) *m/z*: Calcld for $C_{22}H_{18}BrN_2O_4$ [M+H]$^+$ 453.0444; Found: 453.0452.

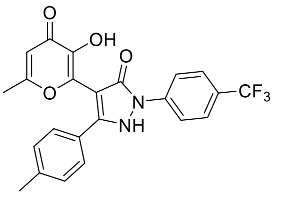

4-(3-Hydroxy-6-methyl-4-oxo-4H-pyran-2-yl)-5-(p-tolyl)-2-(4-(trifluoromethyl)phenyl)-1,2-dihydro-3H-pyrazol-3-one (**8r**).

Yellow powder; yield 58% (0.26 g); mp 224-226°C. ^{1}H NMR (300 MHz, DMSO-d_6) δ 8.21 (d, *J* = 8.5 Hz, 2H), 7.83 (d, *J* = 8.6 Hz, 2H), 7.44 (d, *J* = 7.8 Hz, 2H), 7.22 (d, *J* = 7.8 Hz, 2H), 6.50 (s, 1H), 2.33 (s, 3H), 2.04 (s, 3H). ^{13}C NMR (75 MHz, DMSO-d_6) δ 168.70, 163.95,

158.87, 149.57, 149.21, 141.97, 139.44, 137.73, 130.73, 128.61, 127.57, 126.13, 119.77, 109.18, 93.81, 20.89, 18.82. HRMS (ESI-TOF) *m/z*: Calcld for $C_{23}H_{18}F_3N_2O_4$ $[M+H]^+$ 443.1213; Found: 443.1214.

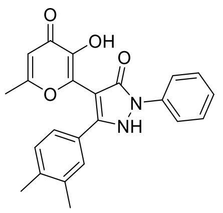

5-(3,4-Dimethylphenyl)-4-(3-hydroxy-6-methyl-4-oxo-4H-pyran-2-yl)-2-phenyl-1,2-dihydro-3H-pyrazol-3-one (**8s**).

White powder; yield 71% (0.28 g); mp 282-284°C. ^{1}H NMR (300 MHz, DMSO-d_6) δ 7.87 (d, *J* = 8.0 Hz, 2H), 7.49 (t, *J* = 7.8 Hz, 2H), 7.36 (s, 1H), 7.33 – 7.20 (m, 2H), 7.14 (d, *J* = 7.8 Hz, 1H), 6.42 (s, 1H), 2.27 – 2.20 (m, 6H), 2.10 (s, 3H). ^{13}C NMR (75 MHz, DMSO-d_6) δ 174.32, 161.83, 160.95, 147.95, 140.15, 138.36, 135.22, 134.95, 132.94, 129.42, 128.52, 125.81, 123.70, 118.90, 110.53, 92.47, 19.43, 19.26, 18.51. HRMS (ESI-TOF) *m/z*: Calcld for $C_{23}H_{21}N_2O_4$ $[M+H]^+$ 389.1496; Found: 389.1510.

5. Characterization data of compounds 10

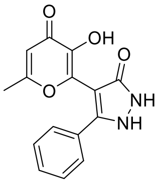

4-(3-Hydroxy-6-methyl-4-oxo-4H-pyran-2-yl)-5-phenyl-1,2-dihydro-3H-pyrazol-3-one (**10a**).

White powder; yield 73% (0.21 g); mp 132-135°C. ^{1}H NMR (300 MHz, DMSO-*d*$_6$) δ 7.49 – 7.28 (m, 5H), 6.25 (s, 1H), 2.13 (s, 3H). ^{13}C NMR (75 MHz, DMSO-*d*$_6$) δ 173.70, 164.80, 142.88, 141.95, 130.14, 128.67, 128.50, 126.45, 111.07, 93.44, 19.27. HRMS (ESI-TOF) *m/z*: Calcld for $C_{15}H_{13}N_2O_4$ [M+H]$^+$ 285.0870; Found: 285.0871.

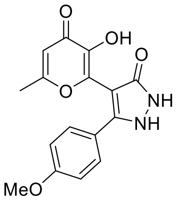

4-(3-Hydroxy-6-methyl-4-oxo-4H-pyran-2-yl)-5-(4-methoxyphenyl)-1,2-dihydro-3H-pyrazol-3-one (**10b**).

Pale yellow powder; yield 68% (0.21 g); mp 243-245°C. ^{1}H NMR (300 MHz, DMSO-*d*$_6$) δ 7.37 (d, *J* = 8.5 Hz, 2H), 6.97 (d, *J* = 8.5 Hz, 2H), 6.24 (s, 1H), 3.76 (s, 3H), 2.14 (s, 3H). ^{13}C NMR (75 MHz, DMSO-*d*$_6$) δ 173.88, 164.92, 159.59, 142.94, 142.40, 142.26, 127.99, 122.45, 114.21, 111.16, 93.14, 55.31, 19.40. HRMS (ESI-TOF) *m/z*: Calcld for $C_{16}H_{15}N_2O_5$ [M+H]$^+$ 315.0975; Found: 315.0975.

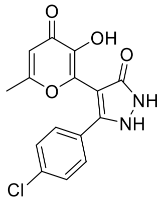

5-(4-Chlorophenyl)-4-(3-hydroxy-6-methyl-4-oxo-4H-pyran-2-yl)-1,2-dihydro-3H-pyrazol-3-one (**10c**).

White powder; yield 81% (0.26 g); mp 267-268°C. ^{1}H NMR (300 MHz, DMSO-*d*$_6$) δ 7.52 – 7.39 (m, 4H), 6.25 (s, 1H), 2.16 (s, 3H). ^{13}C NMR (75 MHz, DMSO-*d*$_6$) δ 173.59, 164.84, 142.79, 141.64, 133.00, 128.69, 128.07, 111.03, 19.30. HRMS (ESI-TOF) *m/z*: Calcld for $C_{15}H_{12}ClN_2O_4$ [M+H]$^+$ 319.0480; Found: 319.0495.

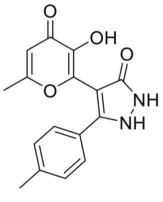

4-(3-Hydroxy-6-methyl-4-oxo-4H-pyran-2-yl)-5-(p-tolyl)-1,2-dihydro-3H-pyrazol-3-one (**10d**).

Pale yellow powder; yield 60% (0.18 g); mp 244-246°C. ^{1}H NMR (300 MHz, DMSO-*d*$_6$) δ 7.32 (d, *J* = 8.0 Hz, 2H), 7.20 (d, *J* = 7.9 Hz, 2H), 6.24 (s, 1H), 2.30 (s, 3H), 2.14 (s, 3H). ^{13}C NMR (75 MHz, DMSO-*d*$_6$) δ 173.68, 164.74, 142.87, 141.99, 137.99, 129.20, 126.30, 111.02, 20.82, 19.28. HRMS (ESI-TOF) *m/z*: Calcld for $C_{16}H_{15}N_2O_4$ [M+H]$^+$ 299.1026; Found: 299.1030.

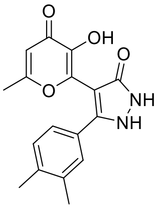

5-(3,4-Dimethylphenyl)-4-(3-hydroxy-6-methyl-4-oxo-4H-pyran-2-yl)-1,2-dihydro-3H-pyrazol-3-one (**10e**).

White powder; yield 63% (0.20 g); mp 177-179°C. ^{1}H NMR (300 MHz, DMSO-*d*$_6$) δ 7.24 (s, 1H), 7.17 – 7.11 (m, 2H), 6.24 (s, 1H), 2.21 (s, 3H), 2.19 (s, 3H), 2.14 (s, 3H). ^{13}C NMR (75 MHz, DMSO-*d*$_6$) δ 173.75, 164.74, 159.49, 142.90, 142.48, 142.13, 136.87, 136.39, 129.72, 127.59, 123.96, 111.05, 93.30, 19.53, 19.29, 19.21. HRMS (ESI-TOF) *m/z*: Calcld for $C_{17}H_{17}N_2O_4$ [M+H]$^+$ 313.1183; Found: 313.1191.

6. Characterization data of compounds 11 and 13

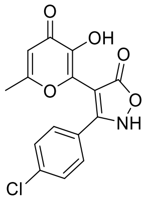

3-(4-Chlorophenyl)-4-(3-hydroxy-6-methyl-4-oxo-4H-pyran-2-yl)isoxazol-5(2H)-one (**11**).

Yellow powder; yield 57% (0.18 g); mp 212-214°C. ^{1}H NMR (300 MHz, DMSO-d_6) δ 7.60 – 7.48 (m, 4H), 6.62 (s, 1H), 6.17 (br. s, 1H), 1.94 (s, 3H). ^{13}C NMR (75 MHz, DMSO-d_6) δ 175.51, 165.48, 164.18, 160.69, 154.13, 135.95, 134.24, 130.35, 129.82, 128.33, 107.98, 84.36 18.55. HRMS (ESI-TOF) *m/z*: Calcld for $C_{15}H_{11}ClNO_5$ [M+H]$^+$ 320.0320; Found: 320.0333.

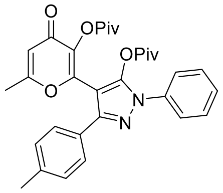

6-Methyl-4-oxo-2-(1-phenyl-5-(pivaloyloxy)-3-(p-tolyl)-1H-pyrazol-4-yl)-4H-pyran-3-yl pivalate (**13**).

White powder; yield 79% (0.43 g); mp 177-179°C. ^{1}H NMR (300 MHz, DMSO-d_6) δ 7.66 – 7.45 (m, 7H), 7.27 (d, *J* = 7.8 Hz, 2H), 6.42 (s, 1H), 2.34 (s, 3H), 2.17 (s, 3H), 1.15 (s, 9H), 1.12 (s, 9H). ^{13}C NMR (75 MHz, DMSO-d_6) δ 179.39, 174.37, 172.36, 165.96, 151.16, 137.94, 129.13, 129.00, 126.57, 121.97, 113.68, 38.30, 37.72, 27.02, 26.47, 20.87, 19.15. HRMS (ESI-TOF) *m/z*: Calcld for $C_{32}H_{35}N_2O_6$ [M+H]$^+$ 543.2490; Found: 543.2509.

7. NMR ^{1}H and ^{13}C spectra for compounds 3

^{1}H NMR spectrum (300 MHz) of **3a** in DMSO-d_6

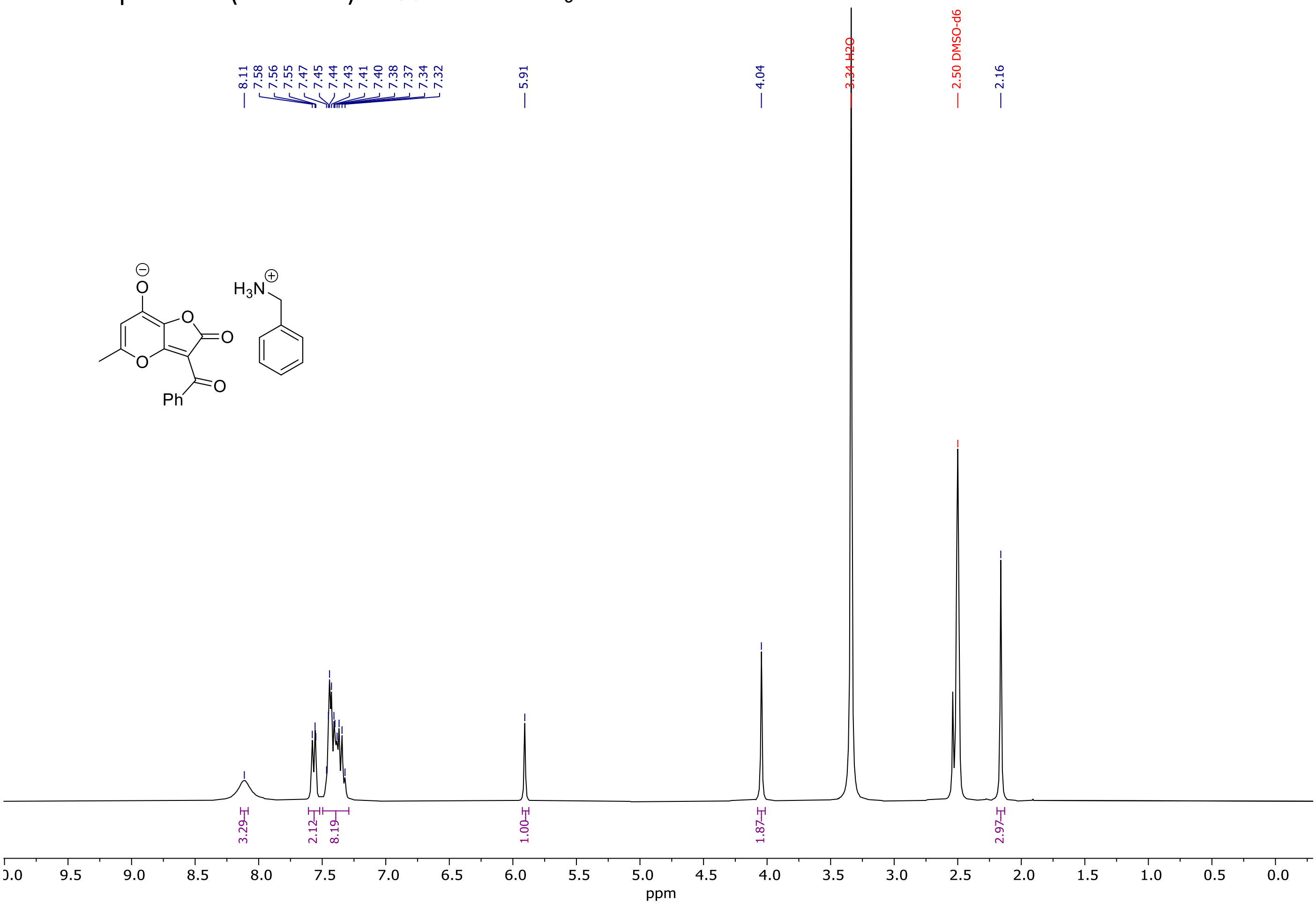

^{13}C {^{1}H} NMR spectrum (75 MHz) of **3a** in DMSO-d_6

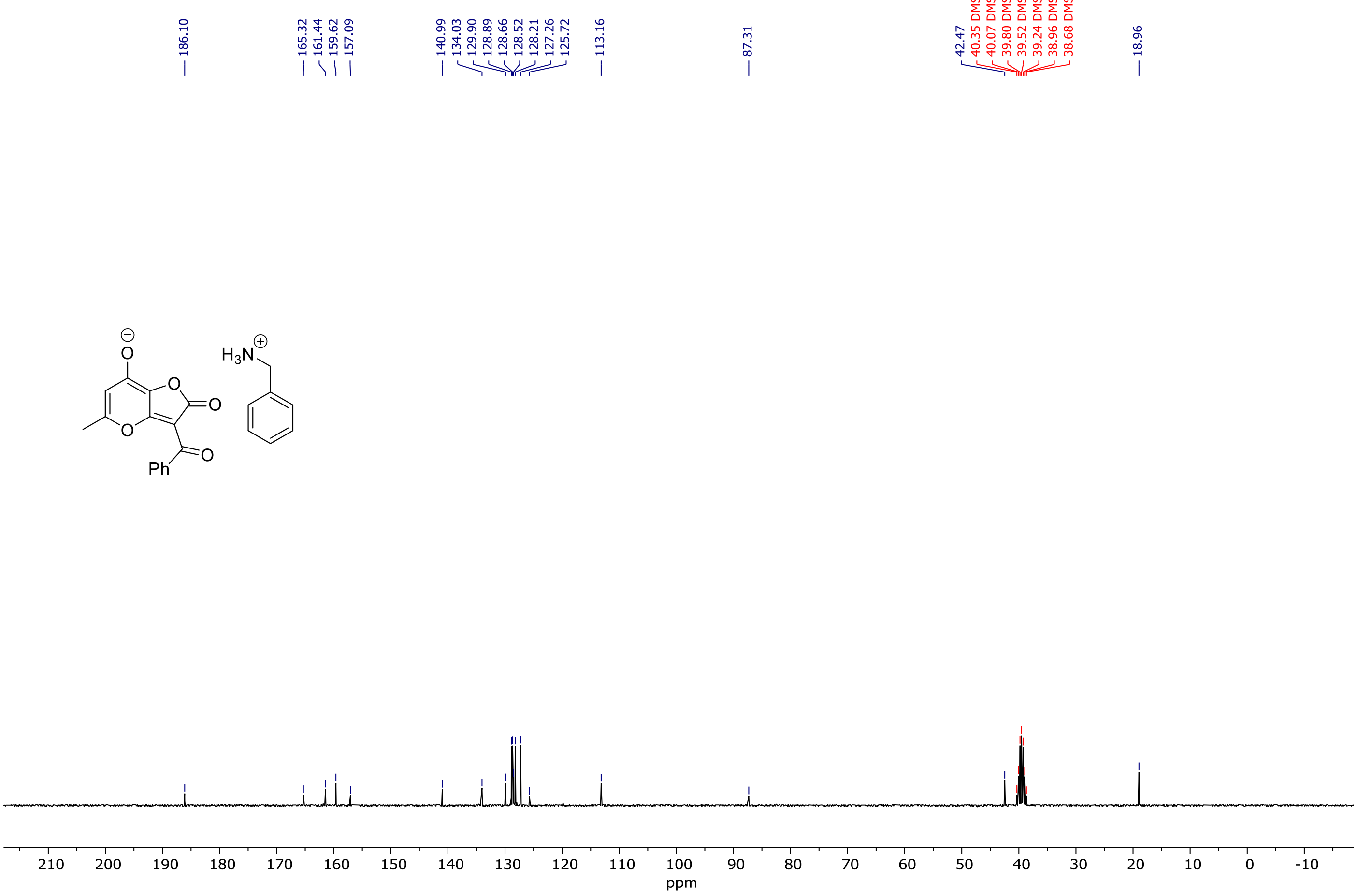

^{1}H NMR spectrum (300 MHz) of **3b** in DMSO-d_6

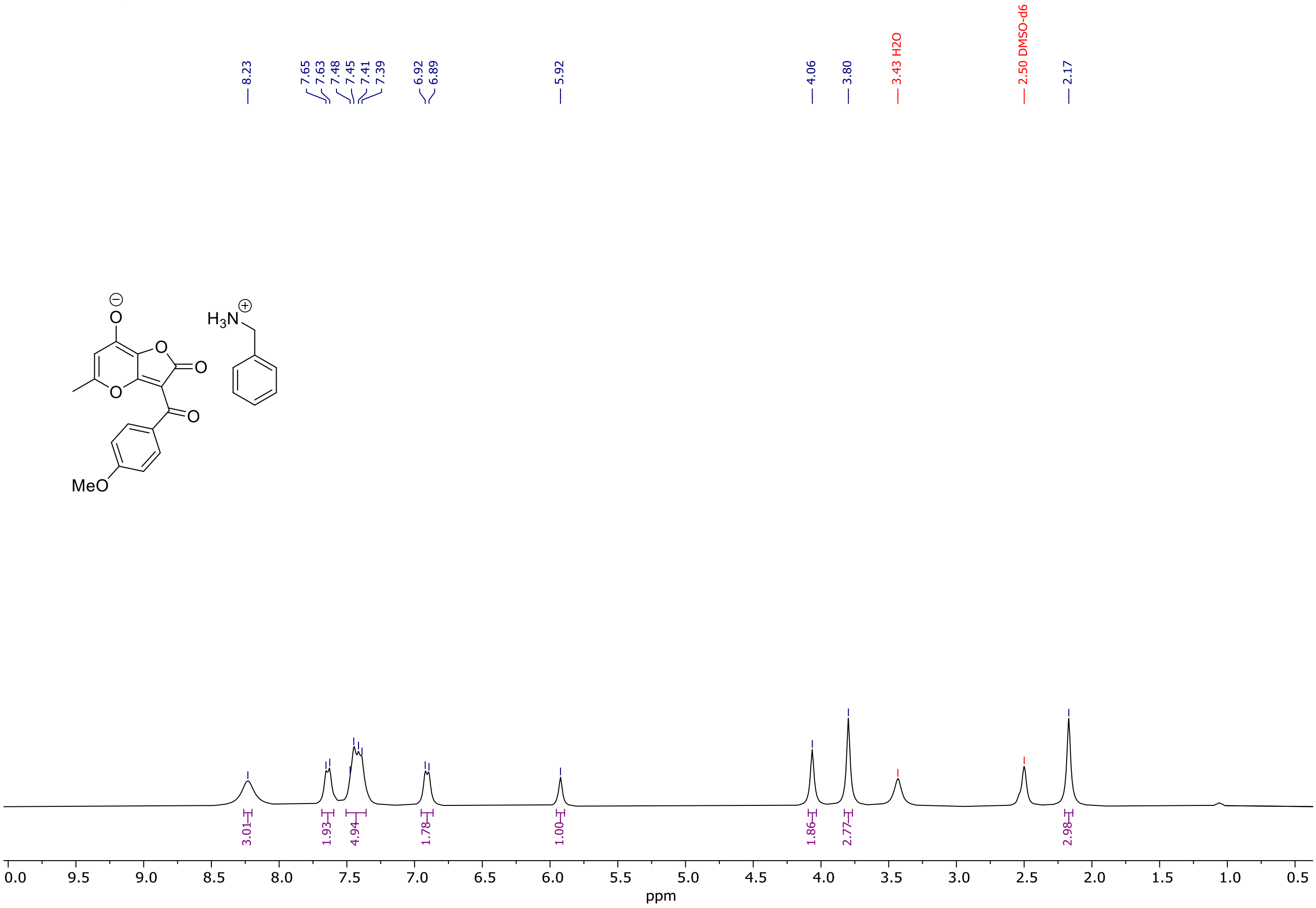

^{13}C {^{1}H} NMR spectrum (75 MHz) of **3b** in DMSO-d_6

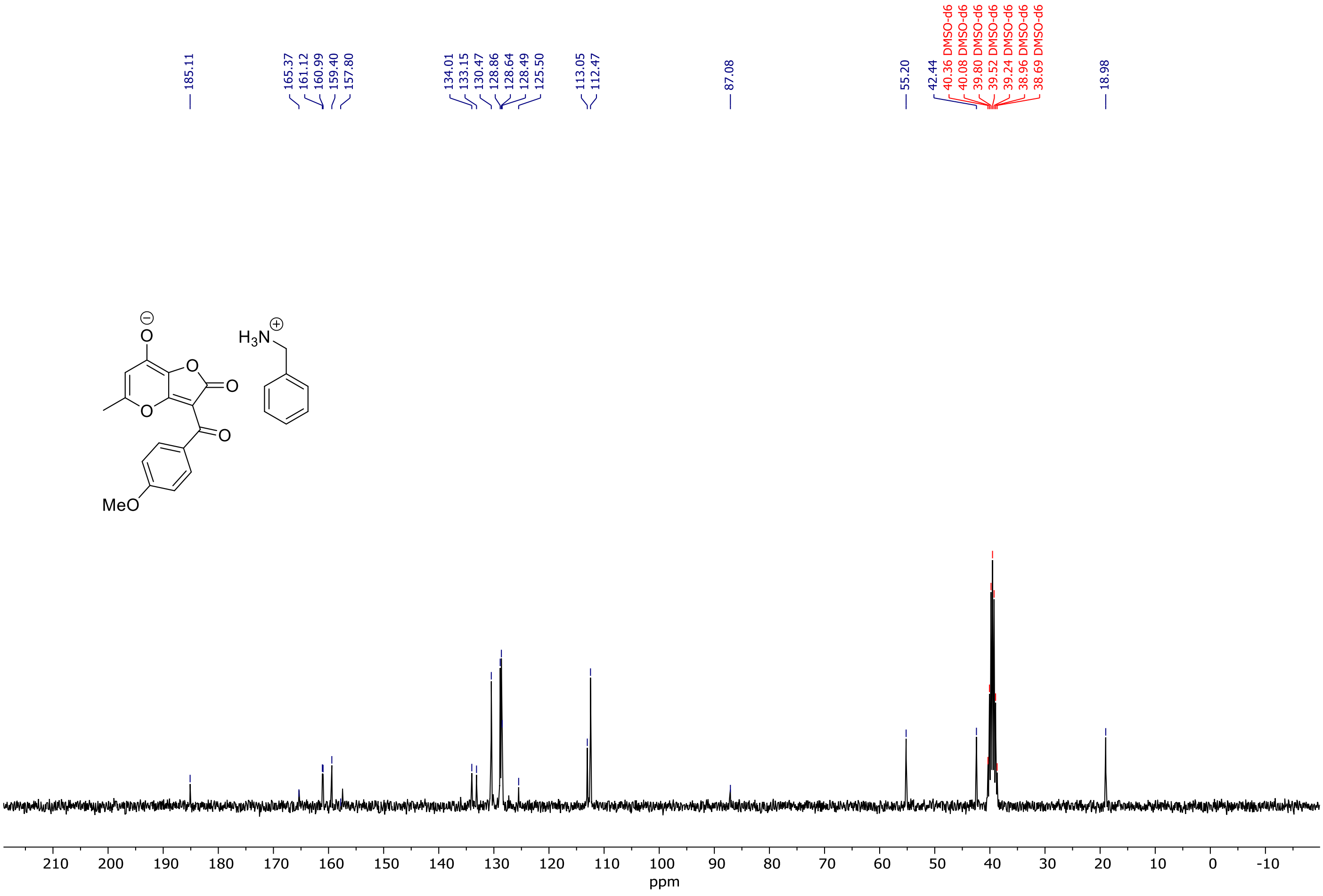

8. NMR ^{1}H and ^{13}C spectra for compounds 4

^{1}H NMR spectrum (300 MHz) of **4a** in DMSO-d_6

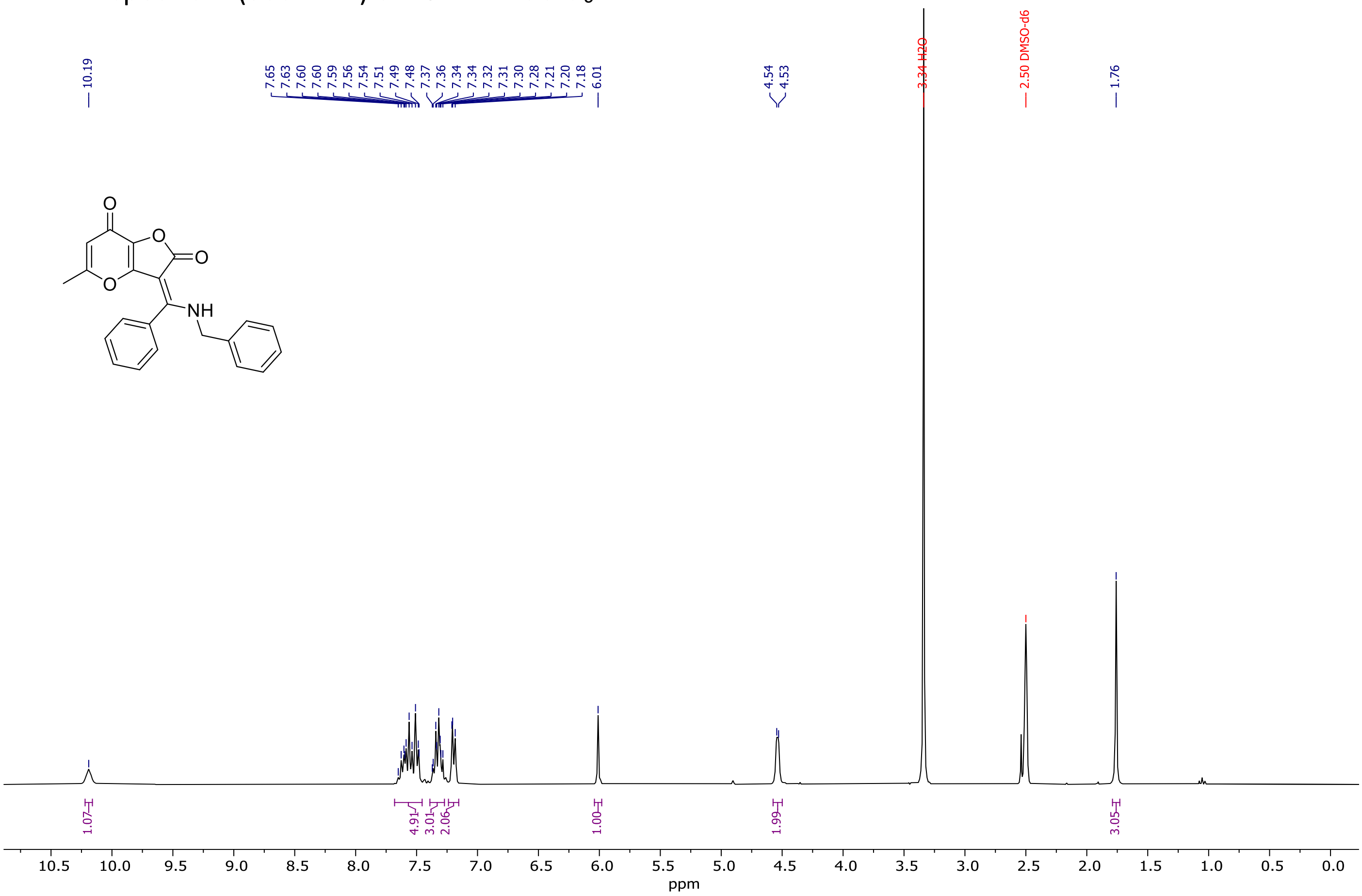

^{13}C {^{1}H} NMR spectrum (75 MHz) of **4a** in DMSO-d_6

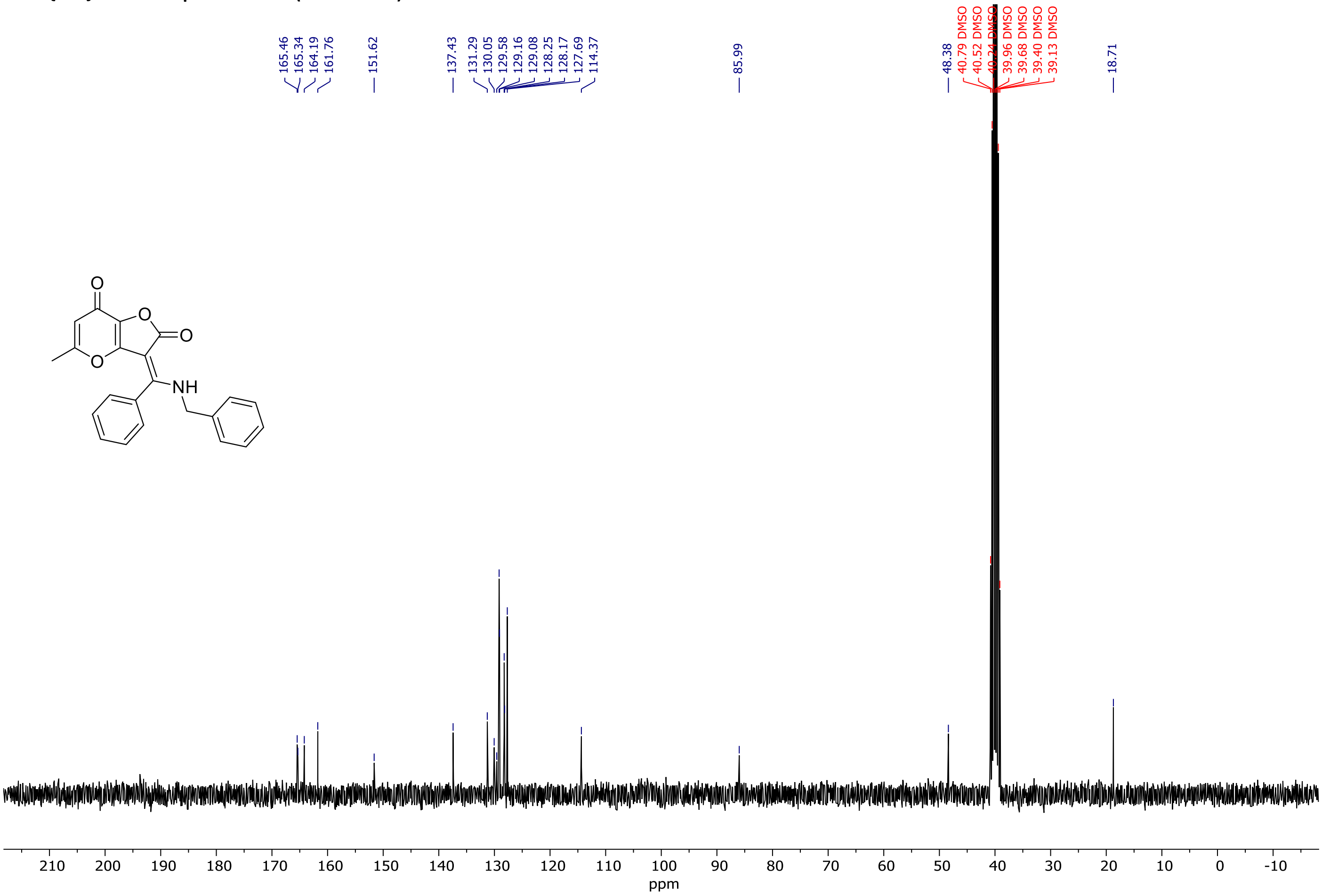

^{1}H NMR spectrum (300 MHz) of **4b** in DMSO-d_6

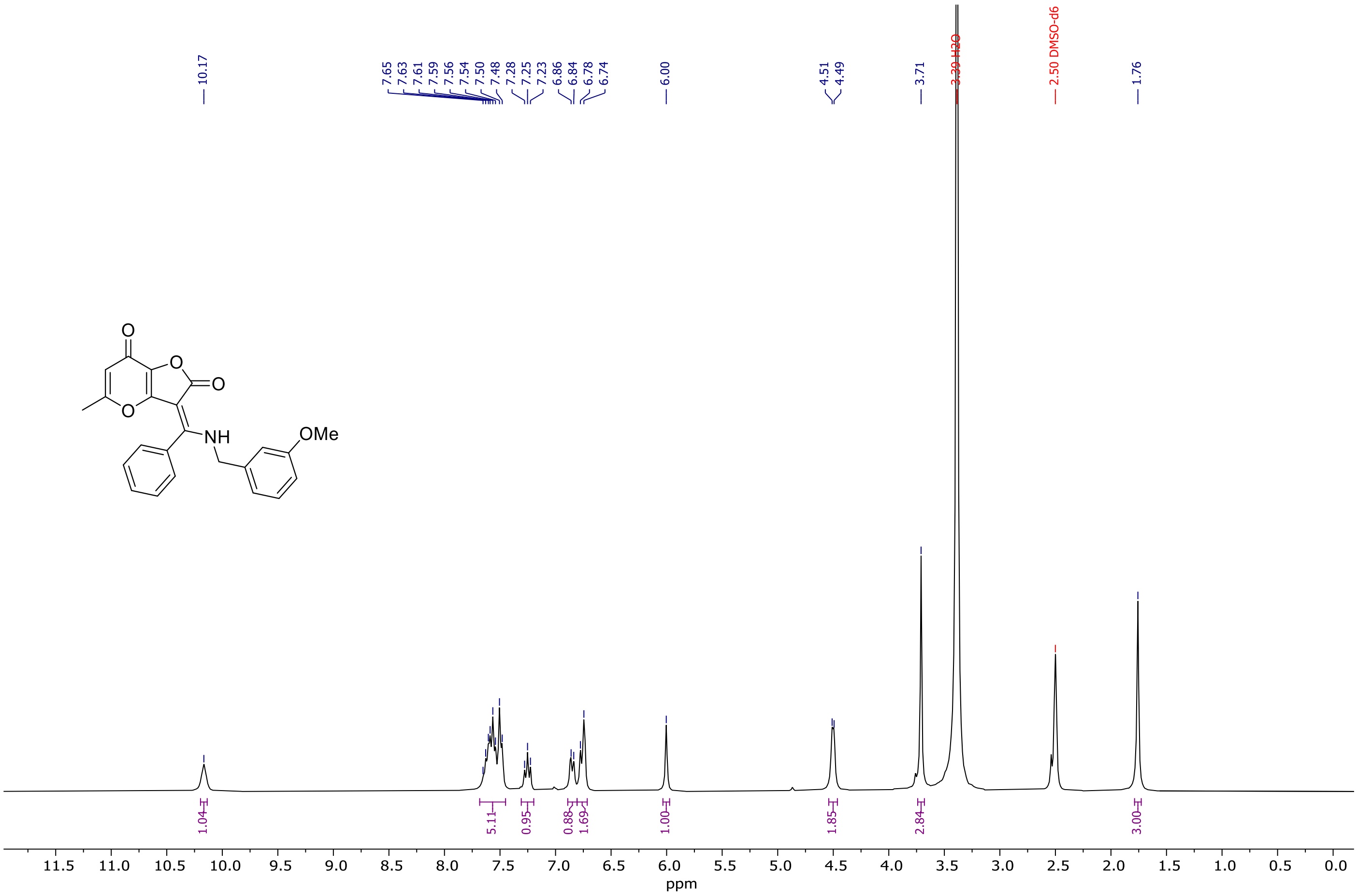

^{13}C {^{1}H} NMR spectrum (75 MHz) of **4b** in DMSO-d_6

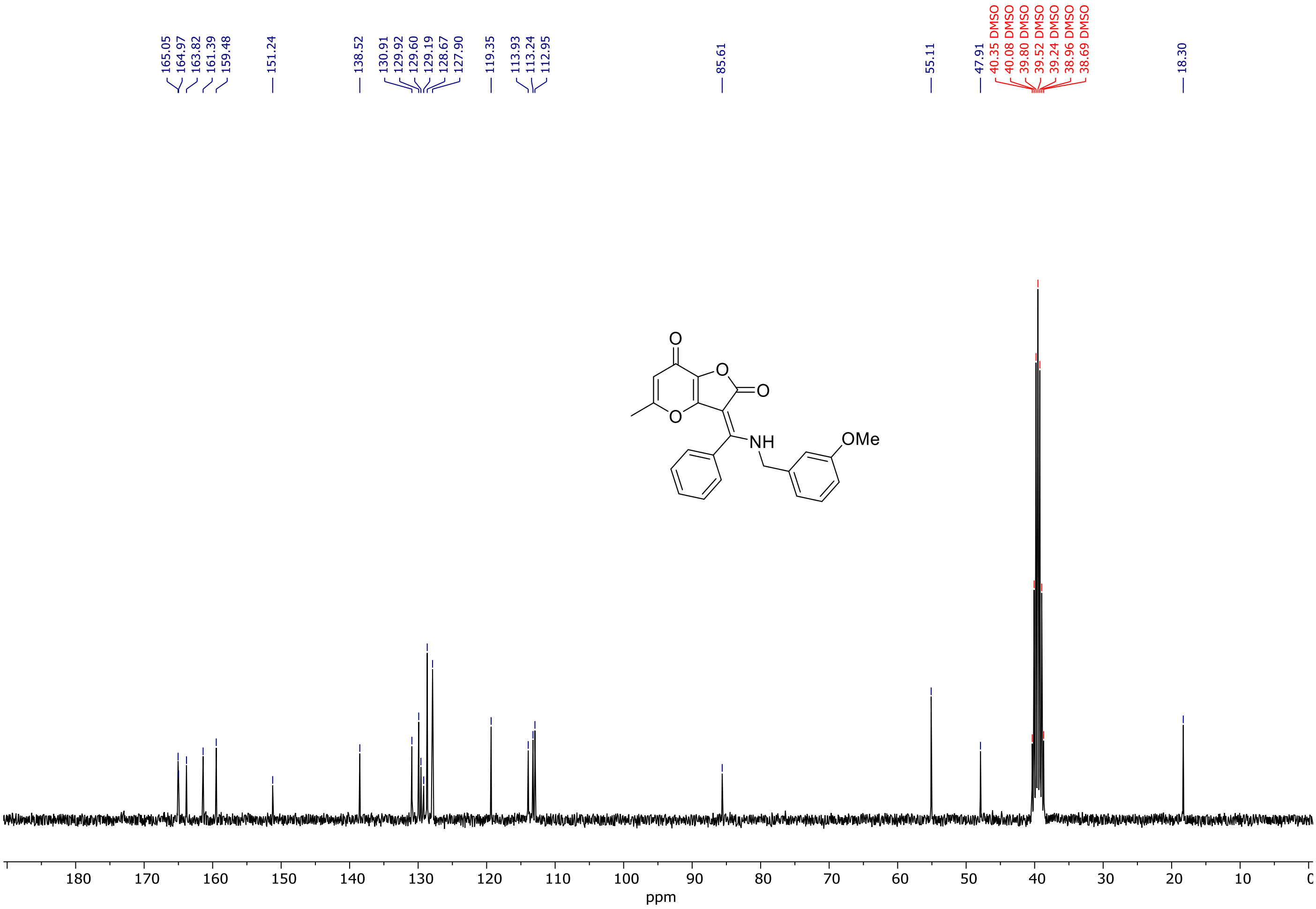

^{1}H NMR spectrum (300 MHz) of **4c** in DMSO-d_6

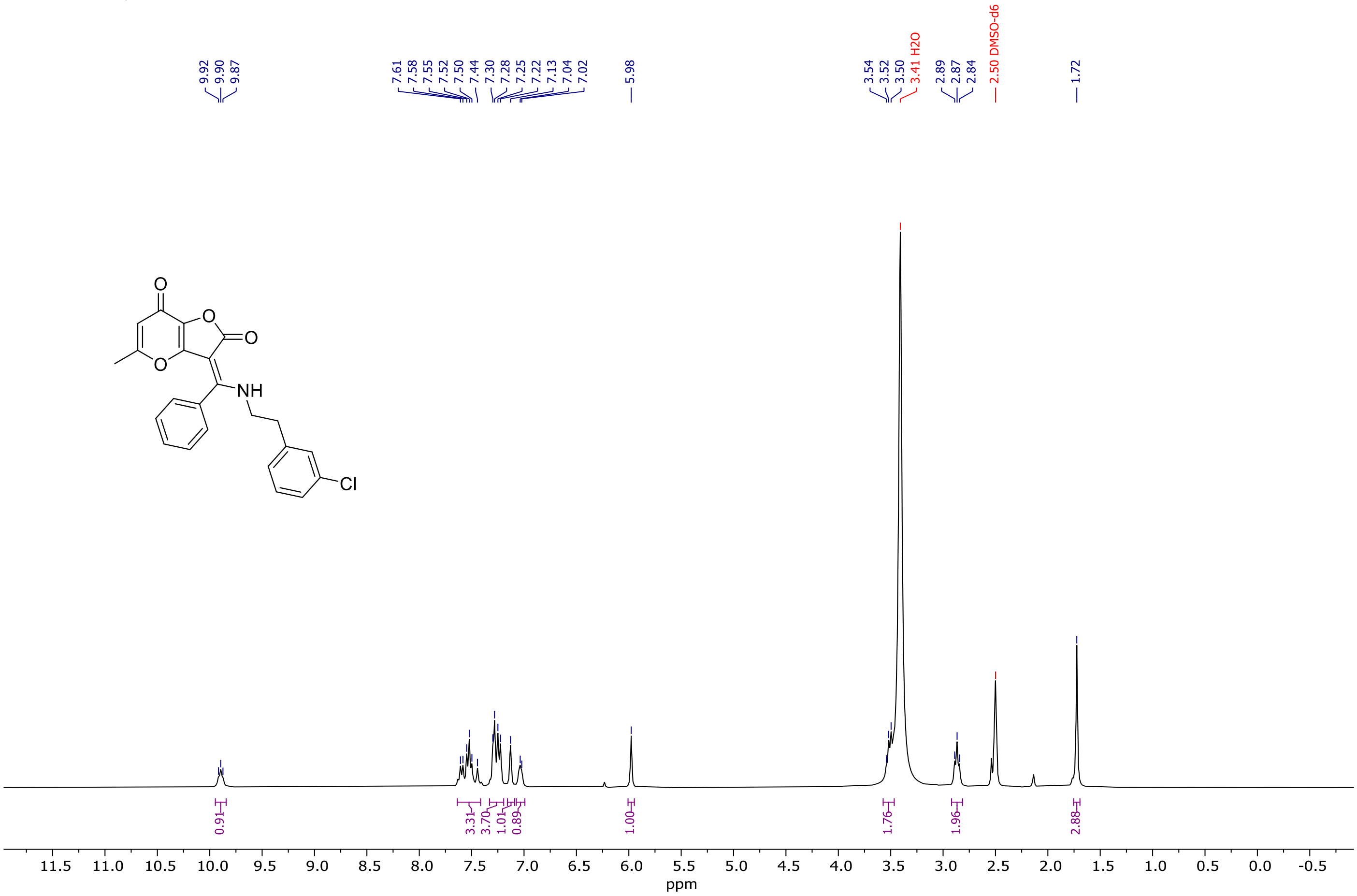

^{13}C {^{1}H} NMR spectrum (75 MHz) of **4c** in DMSO-d_6

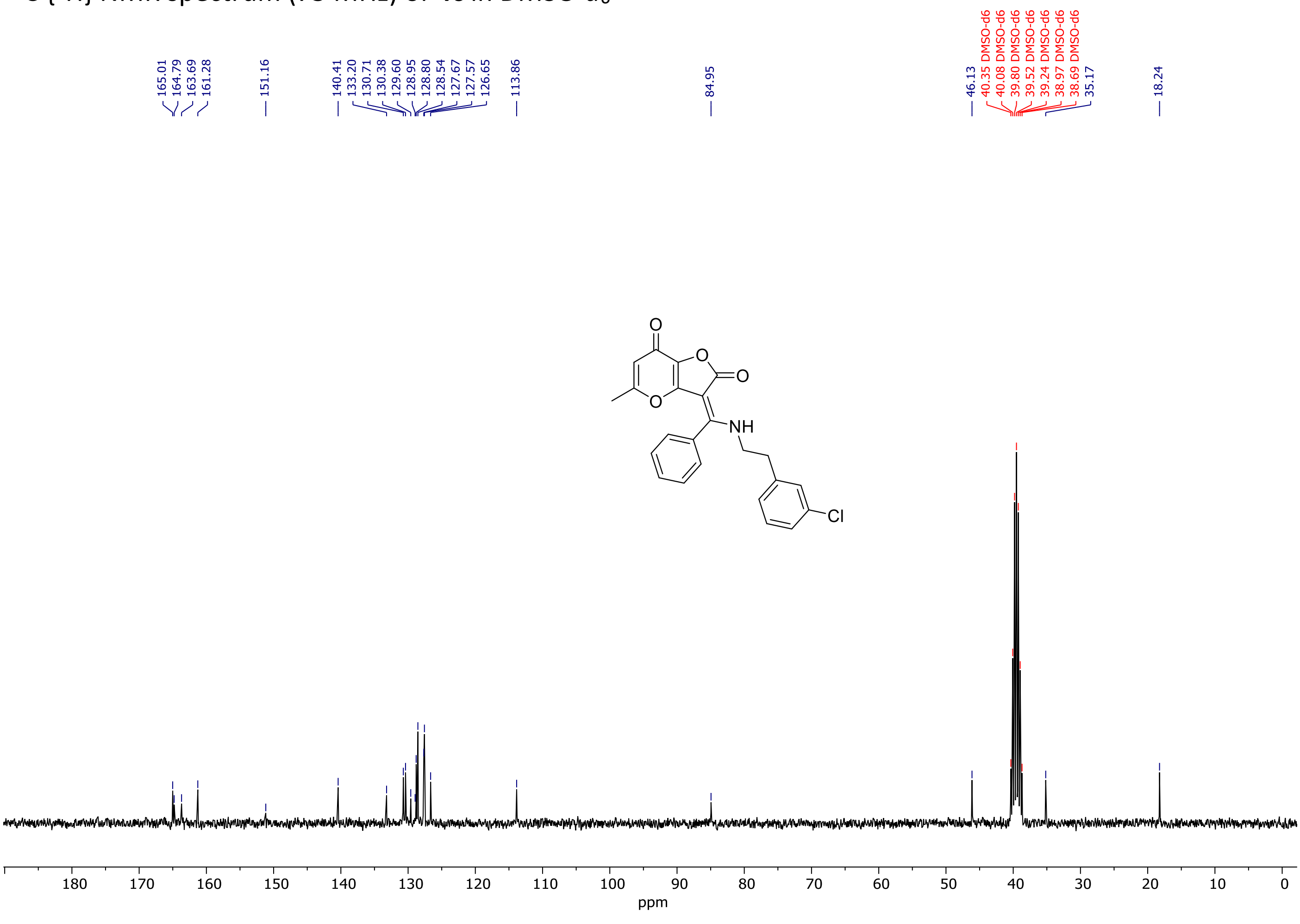

9. NMR ^{1}H and ^{13}C spectra for compounds 8

^{1}H NMR spectrum (300 MHz) of **8a** in DMSO-d_6

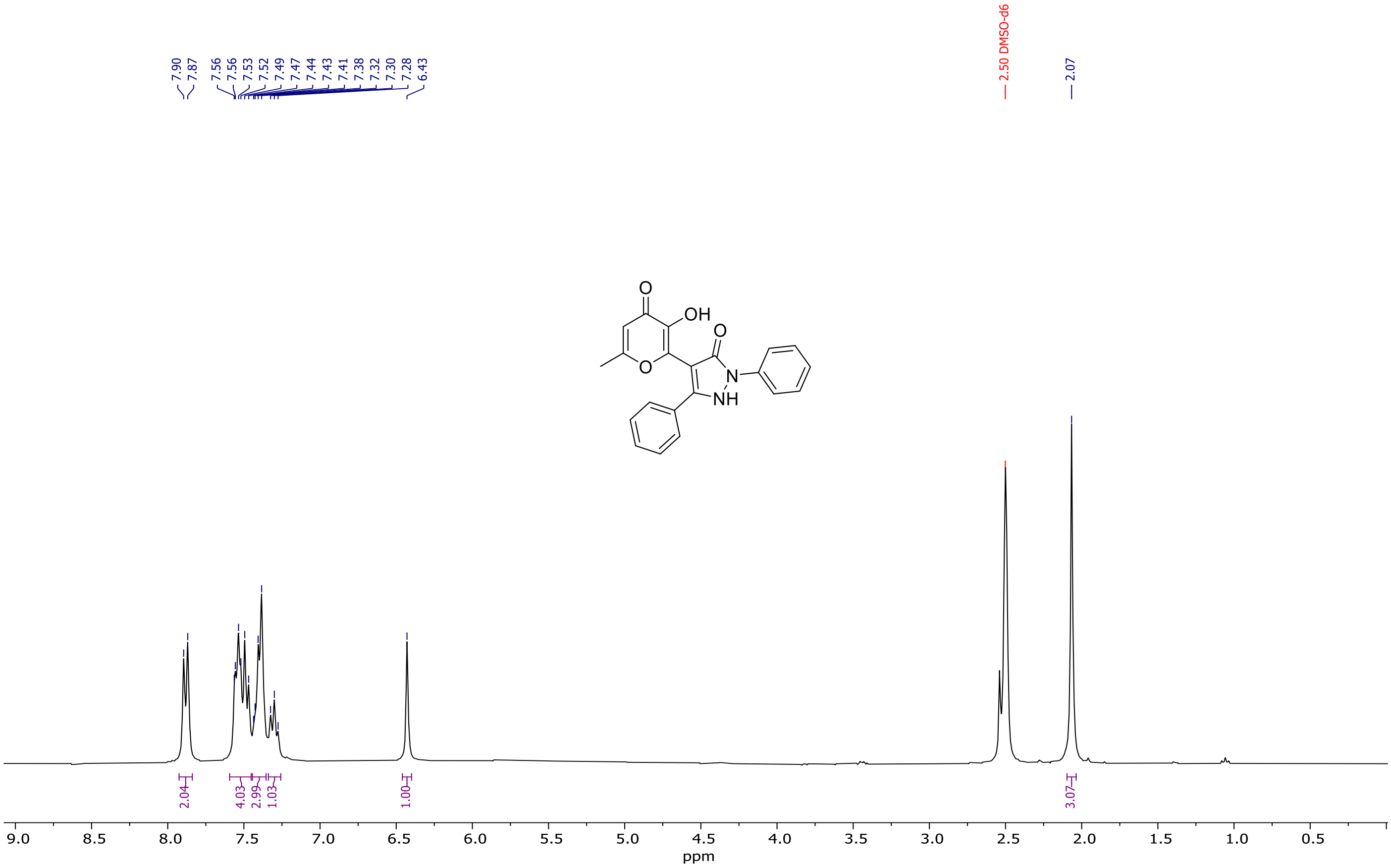

^{13}C {^{1}H} NMR spectrum (75 MHz) of **8a** in DMSO-d_6

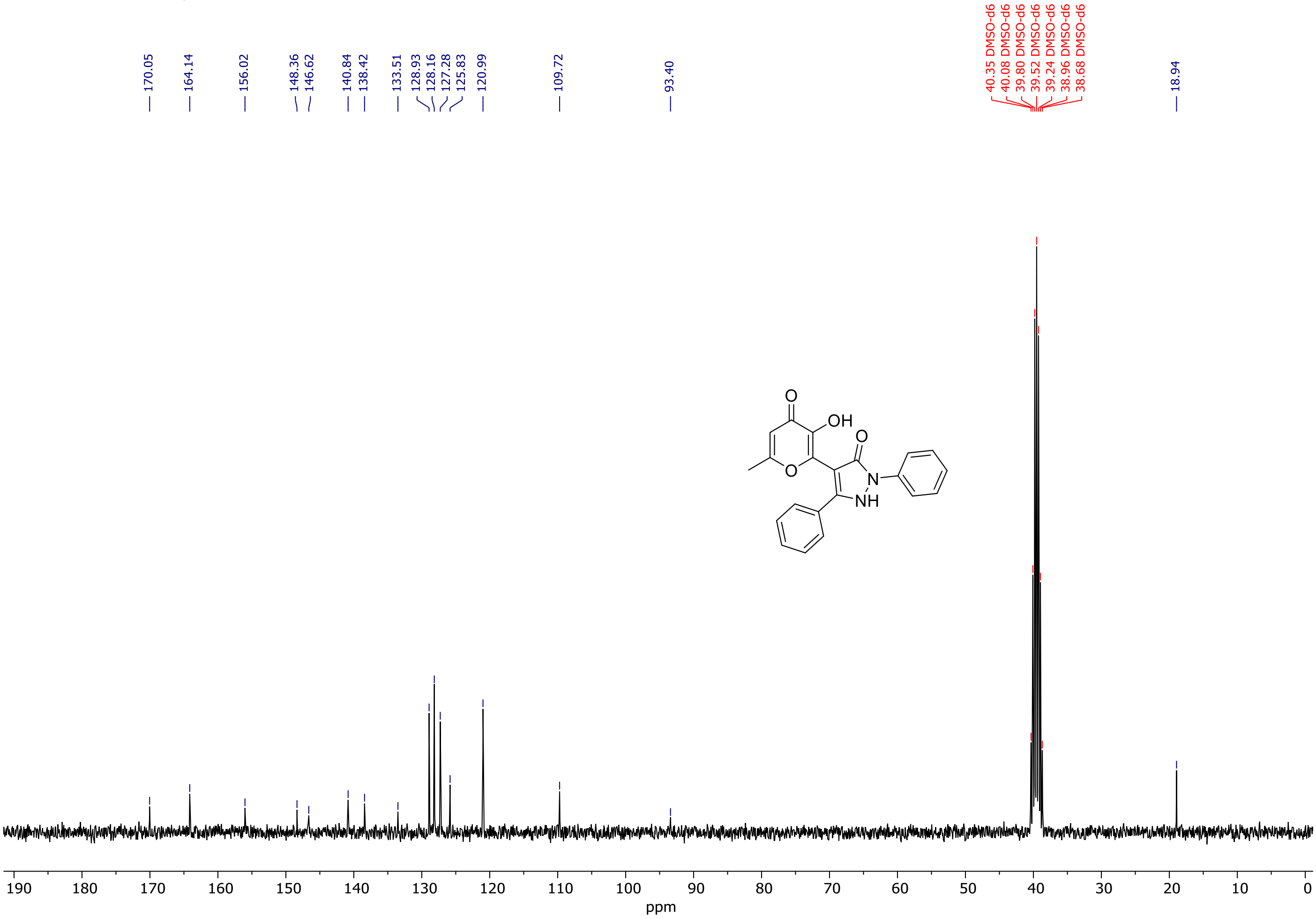

^{1}H NMR spectrum (300 MHz) of **8b** in DMSO-d_6

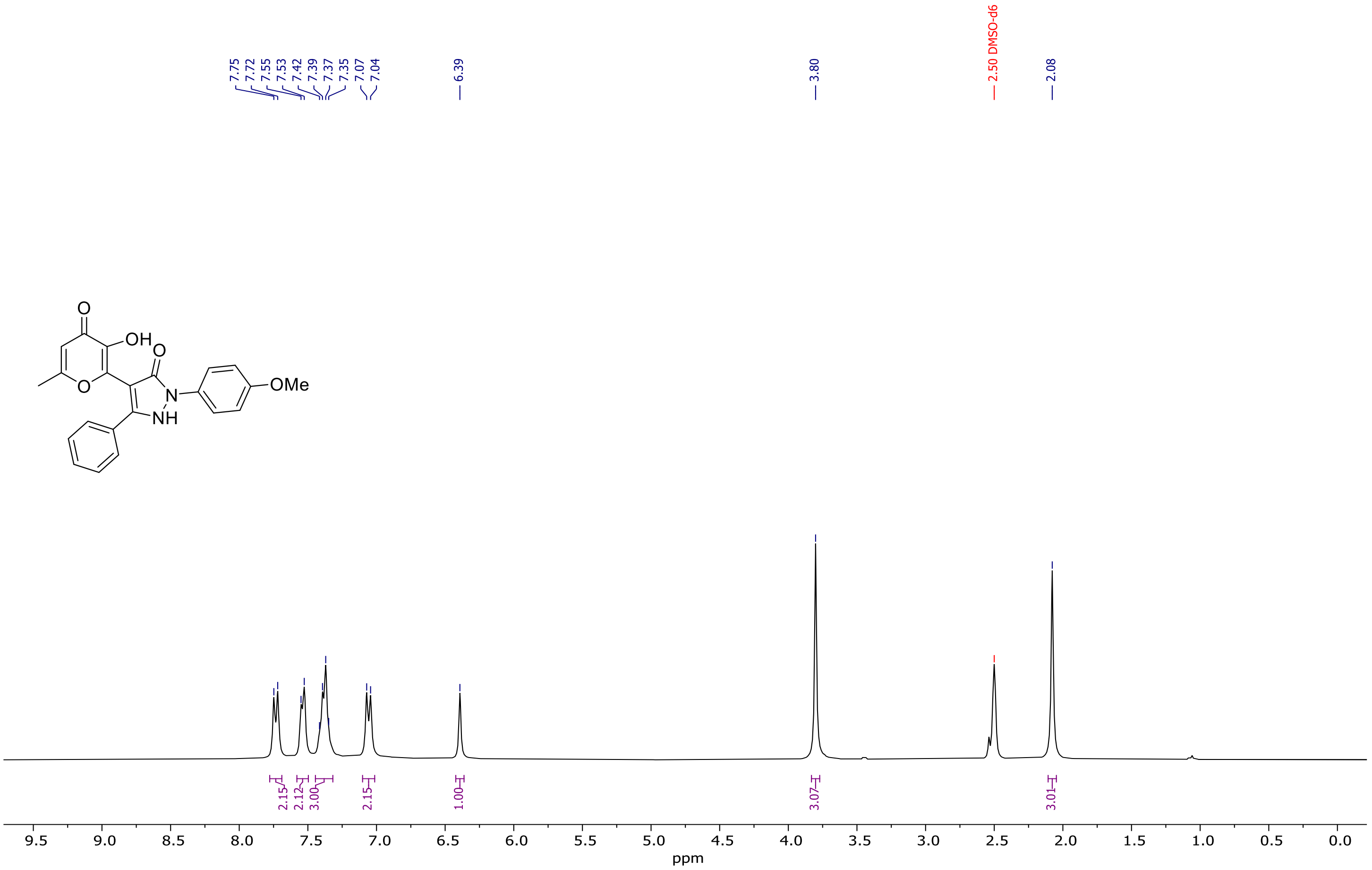

^{13}C {^{1}H} NMR spectrum (75 MHz) of **8b** in DMSO-d_6

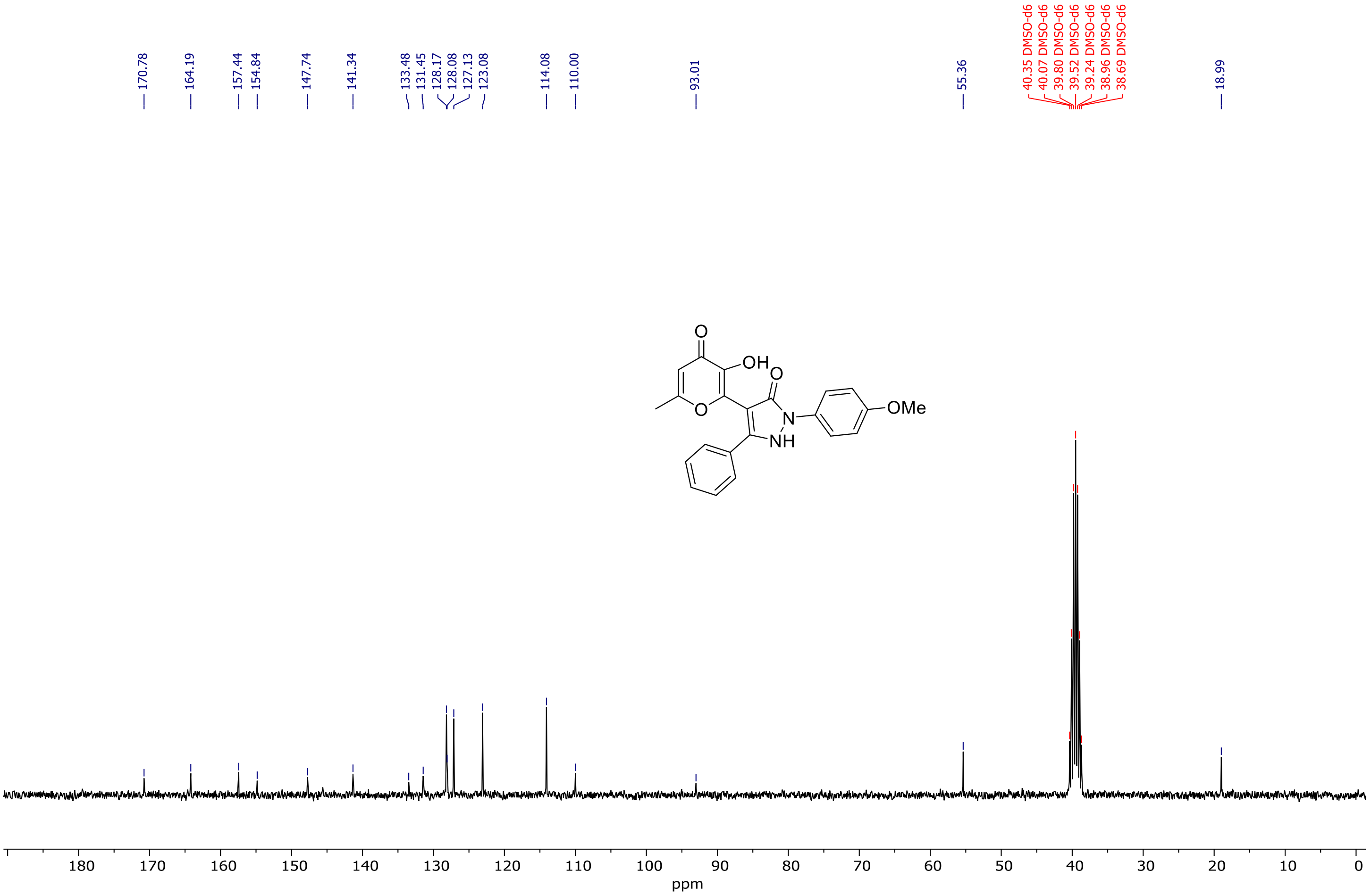

^{1}H NMR spectrum (300 MHz) of **8c** in DMSO-d_6

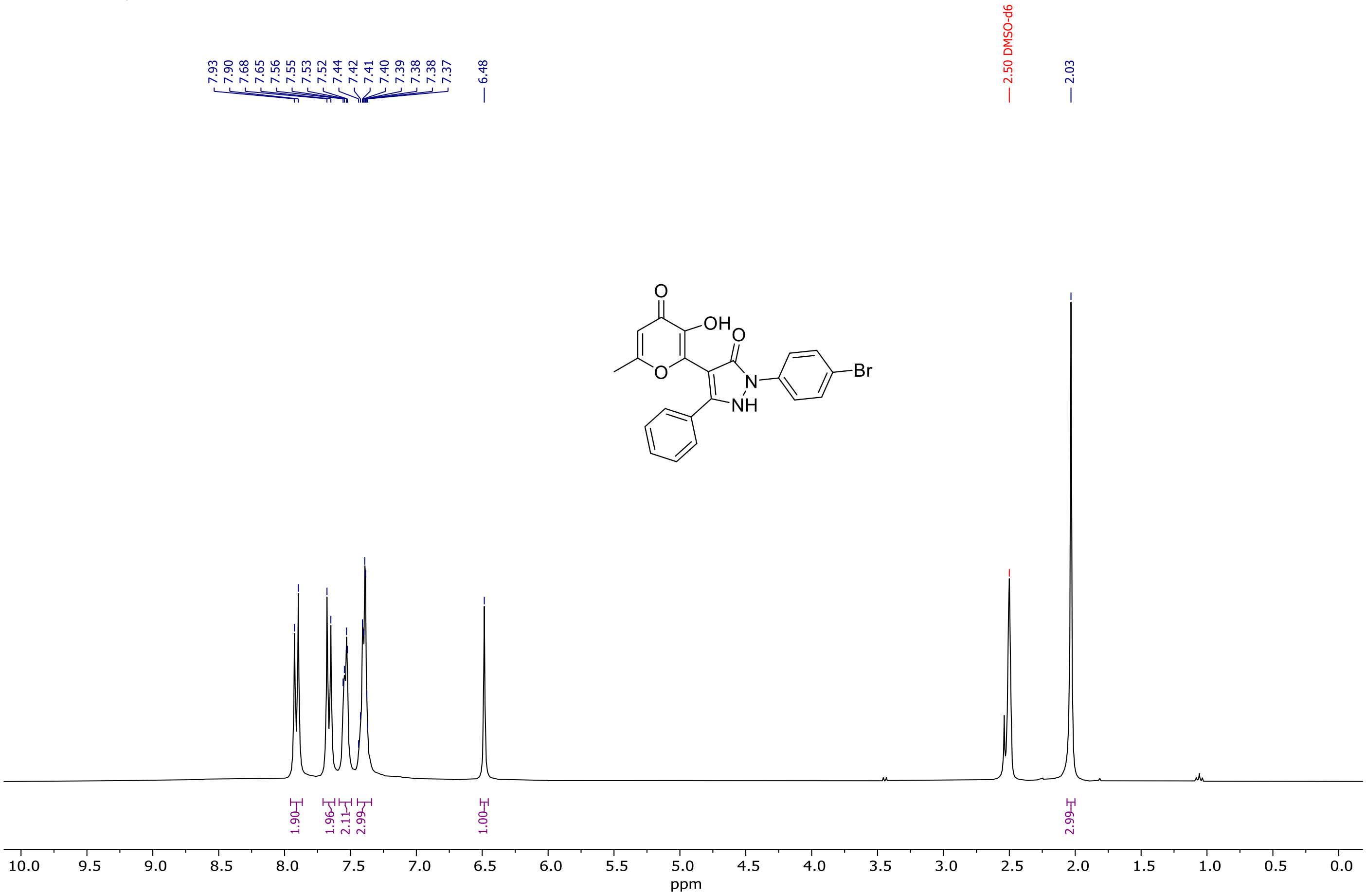

^{13}C {^{1}H} NMR spectrum (75 MHz) of **8c** in DMSO-d_6

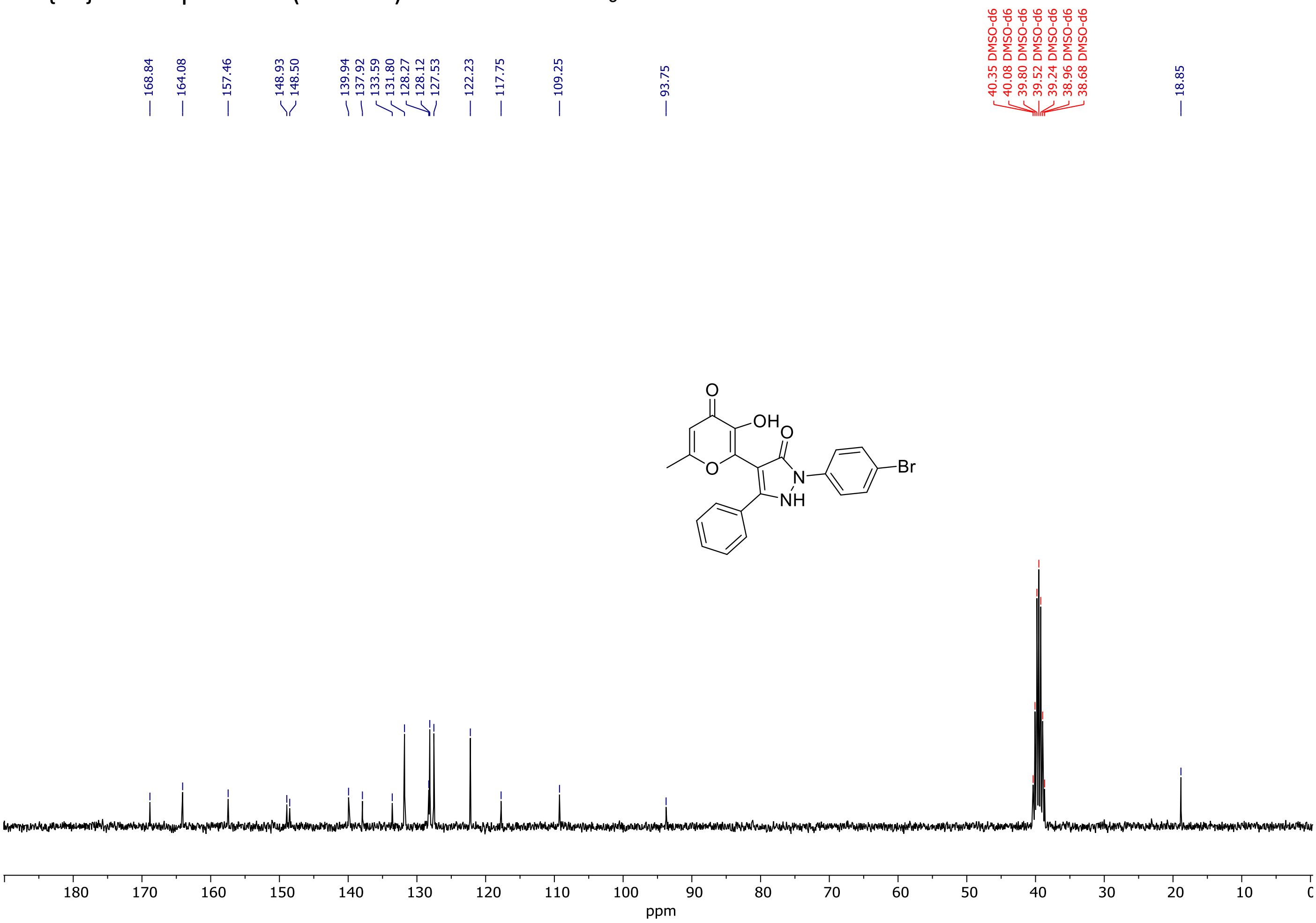

^{1}H NMR spectrum (300 MHz) of **8d** in DMSO-d_6

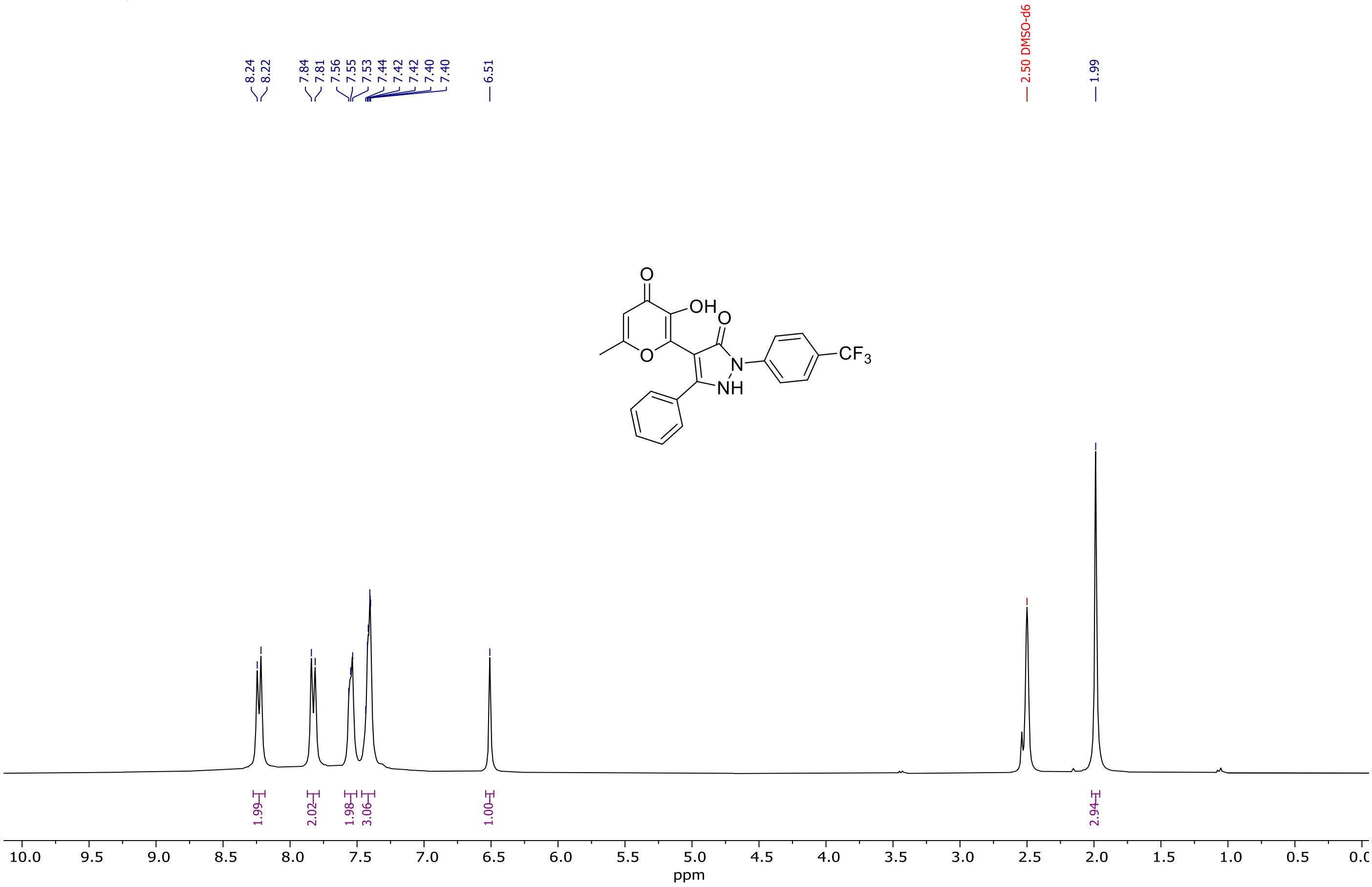

^{13}C {^{1}H} NMR spectrum (75 MHz) of **8d** in DMSO-d_6

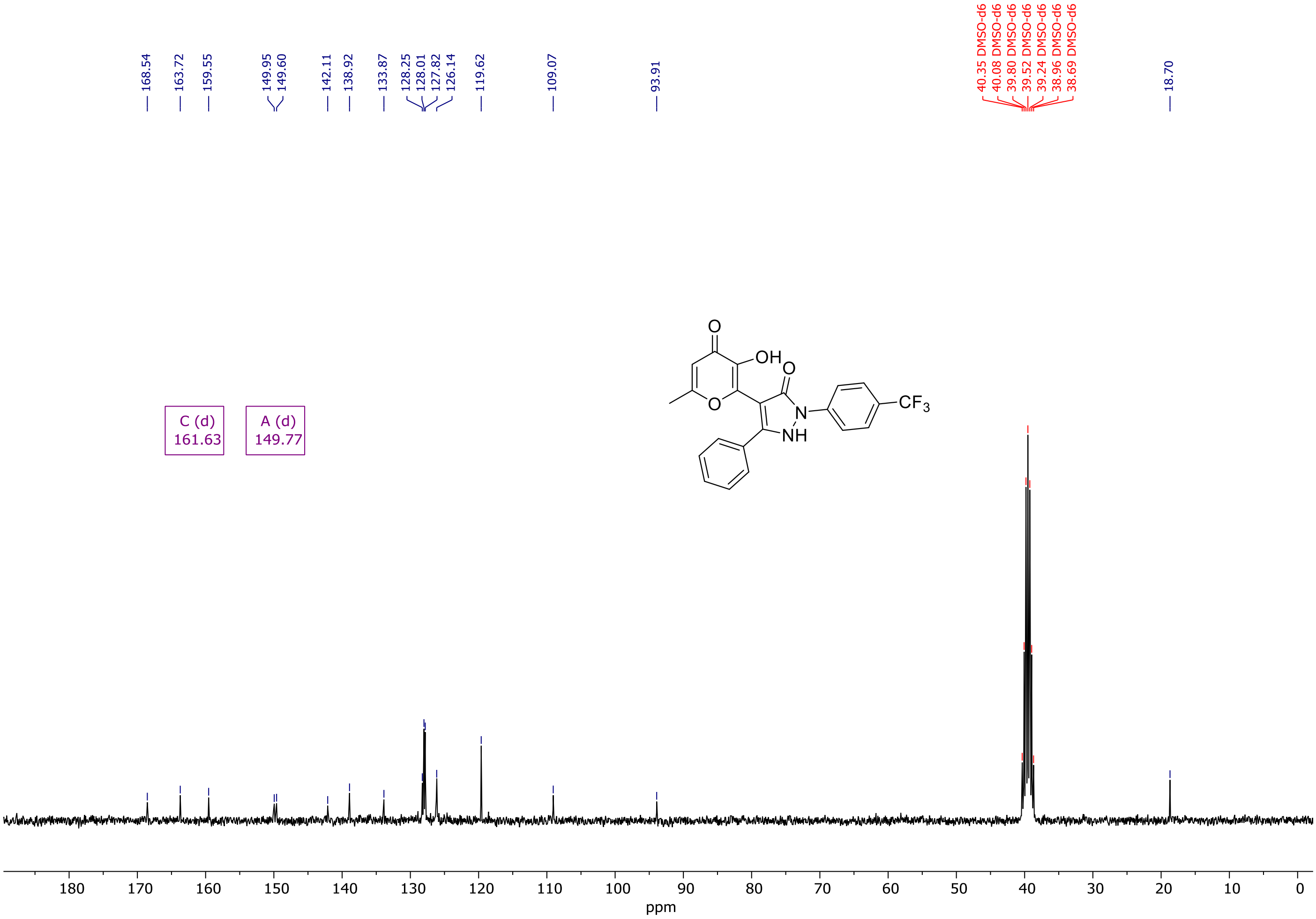

^{1}H NMR spectrum (300 MHz) of **8e** in DMSO-d_6

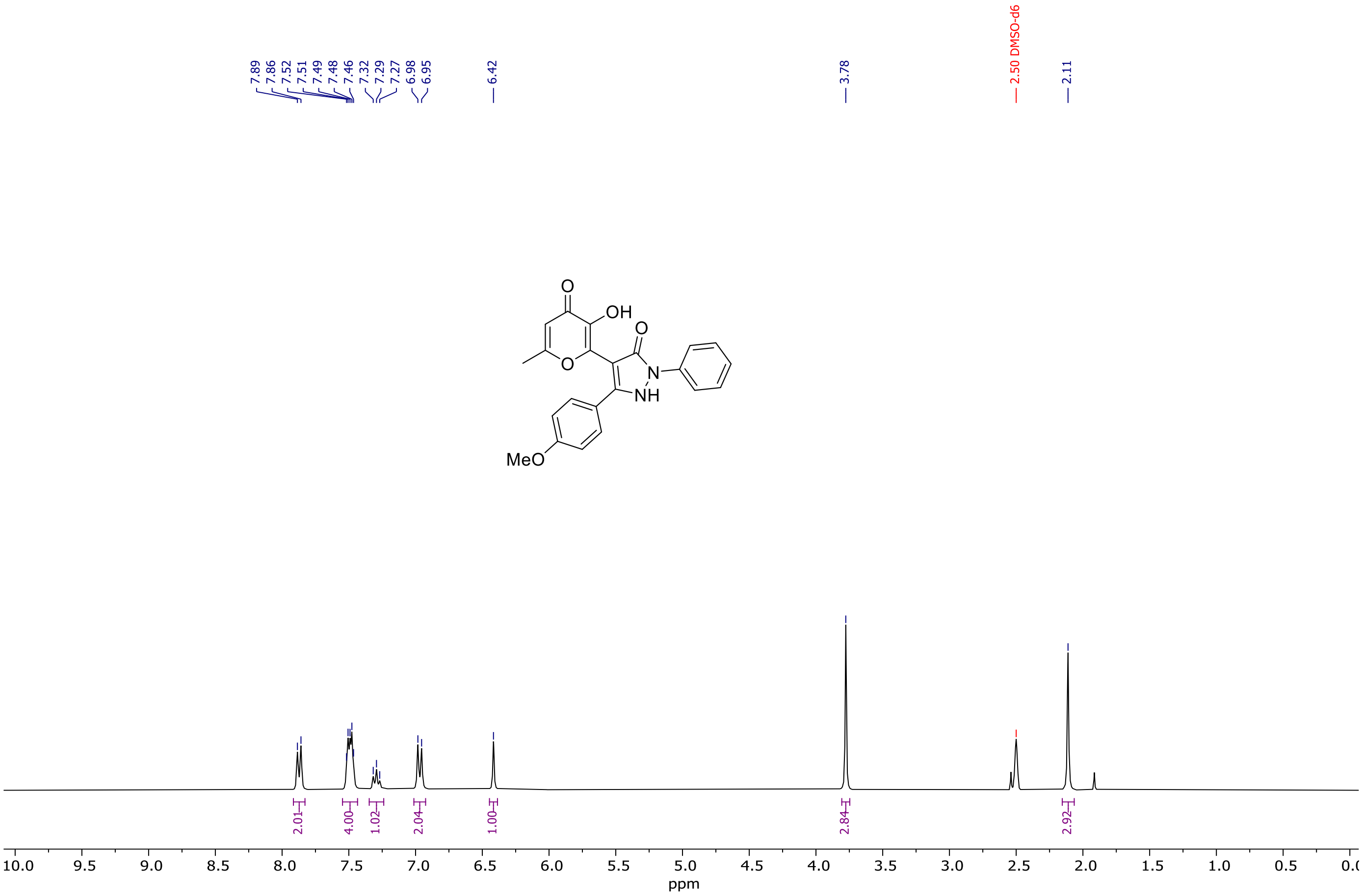

^{13}C {^{1}H} NMR spectrum (75 MHz) of **8e** in DMSO-d_6

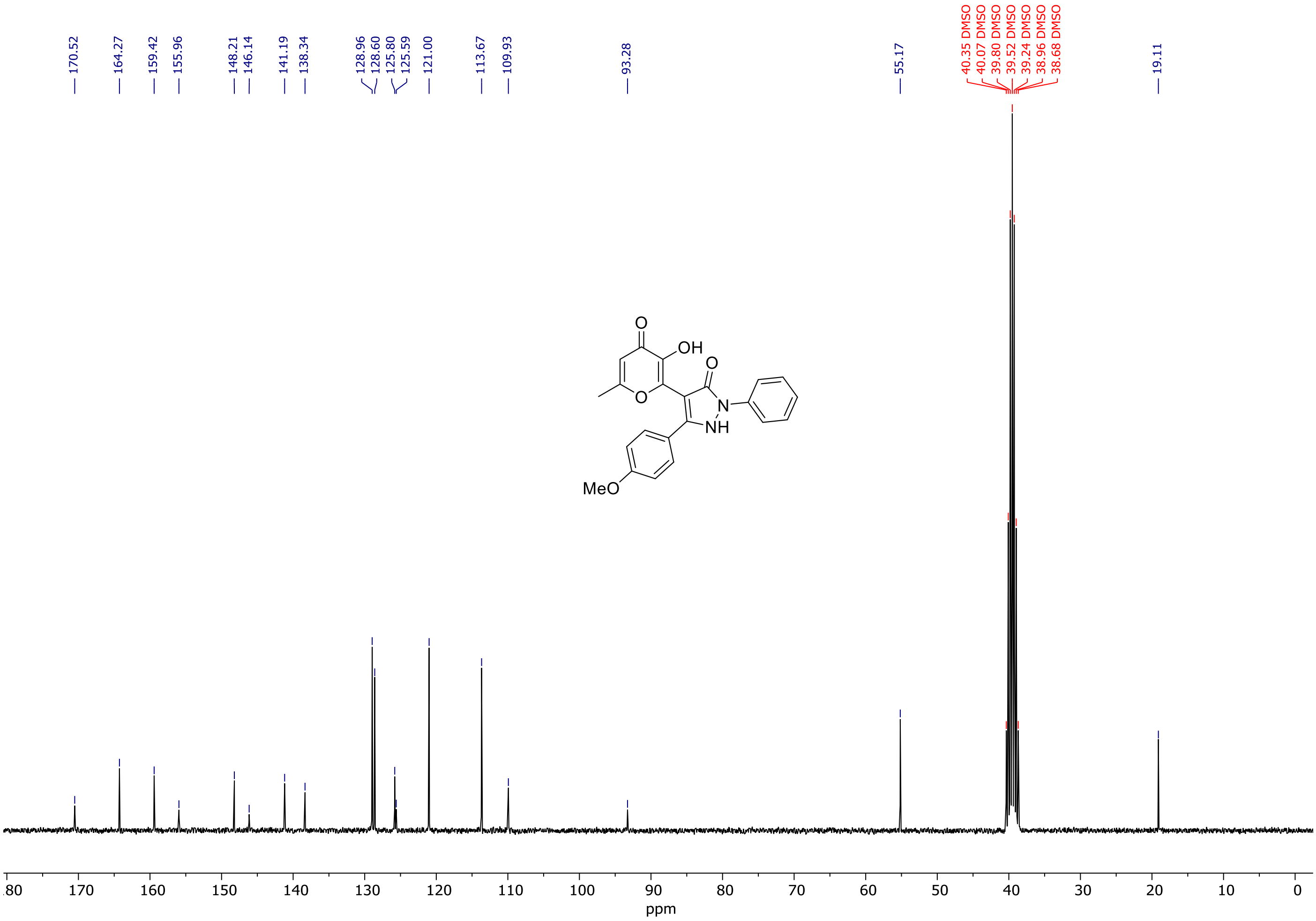

^{1}H NMR spectrum (300 MHz) of **8f** in DMSO-d_6

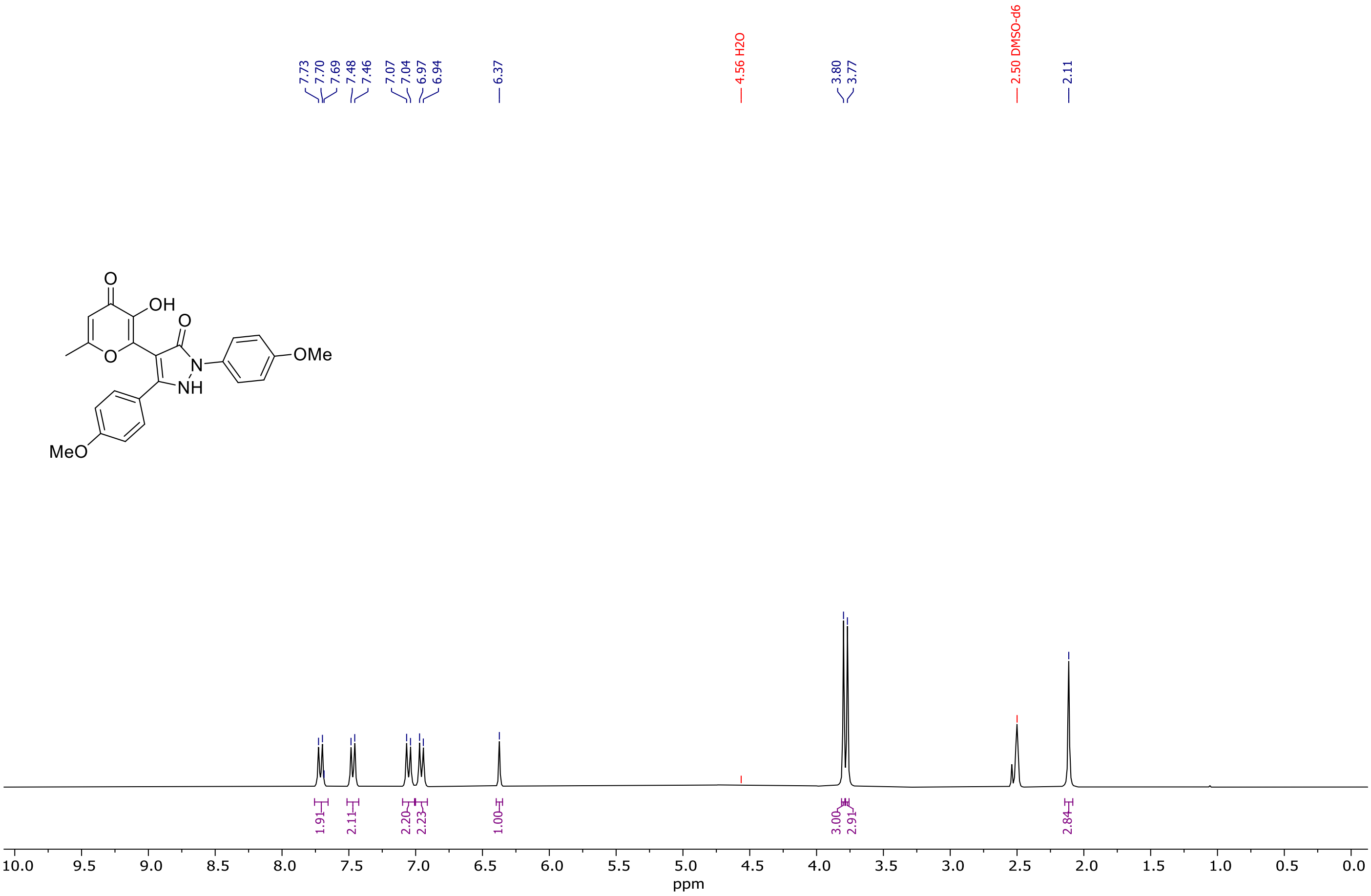

^{13}C {^{1}H} NMR spectrum (75 MHz) of **8f** in DMSO-d_6

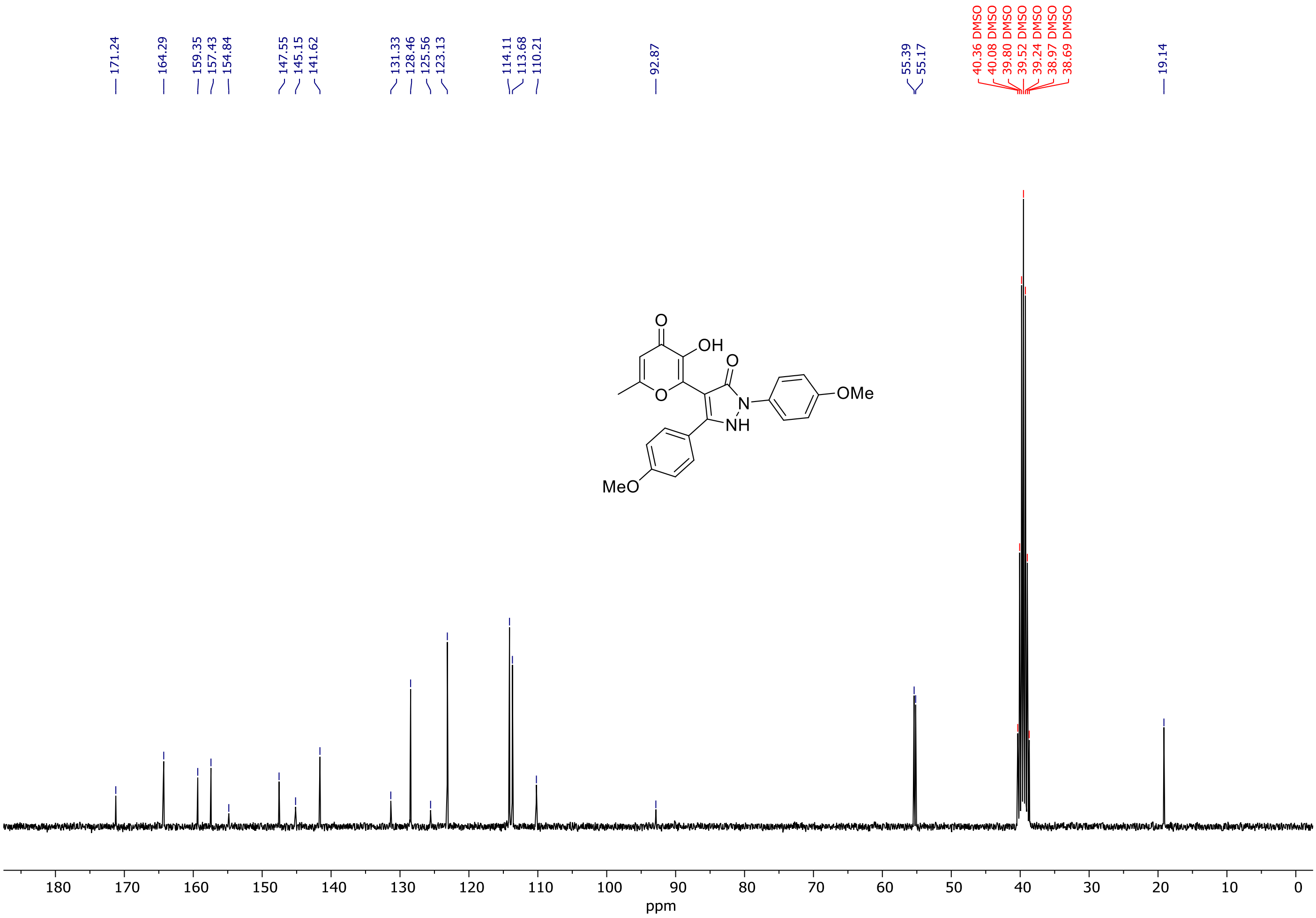

^{1}H NMR spectrum (300 MHz) of **8g** in DMSO-d_6

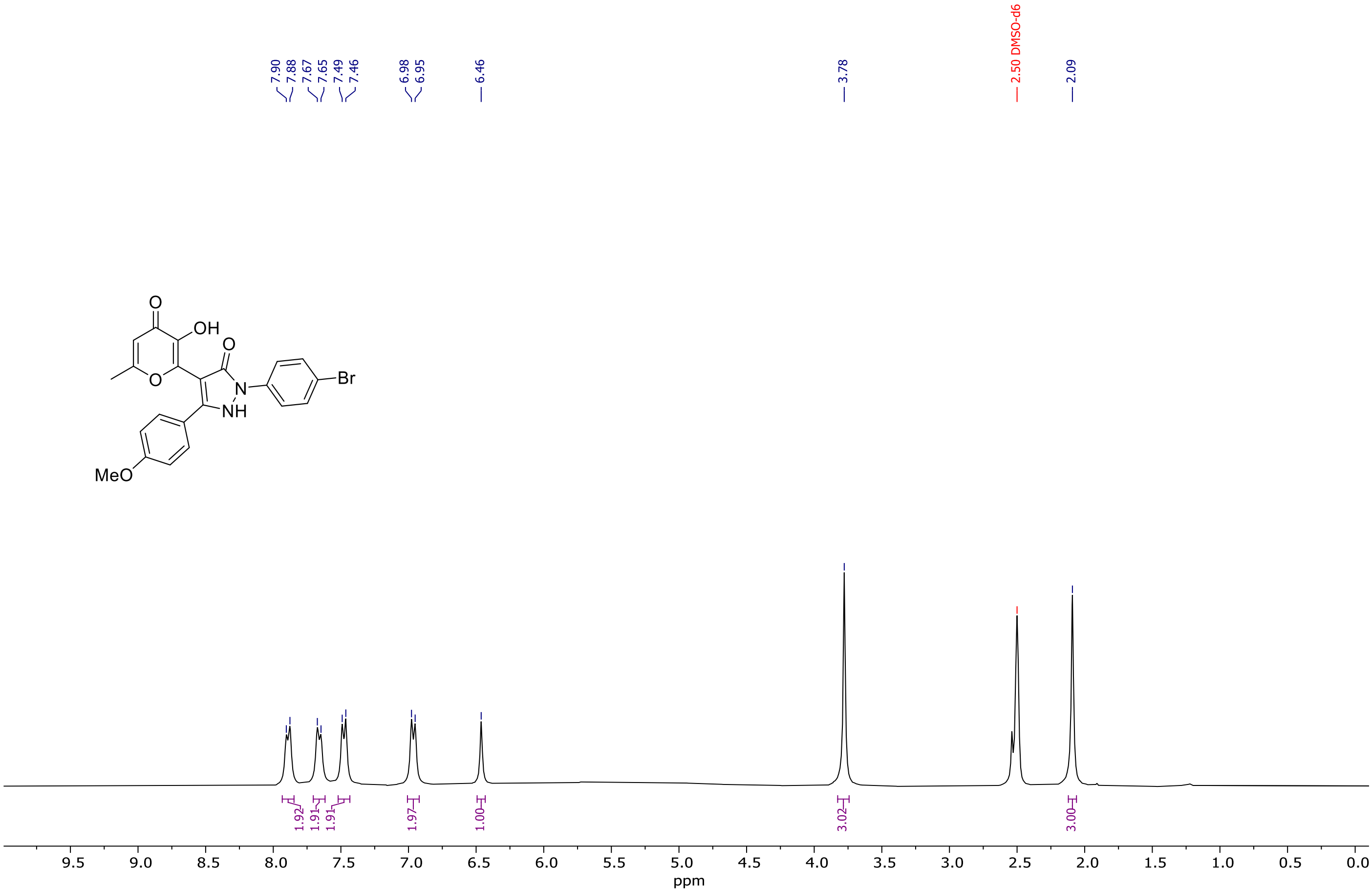

^{13}C {^{1}H} NMR spectrum (75 MHz) of **8g** in DMSO-d_6

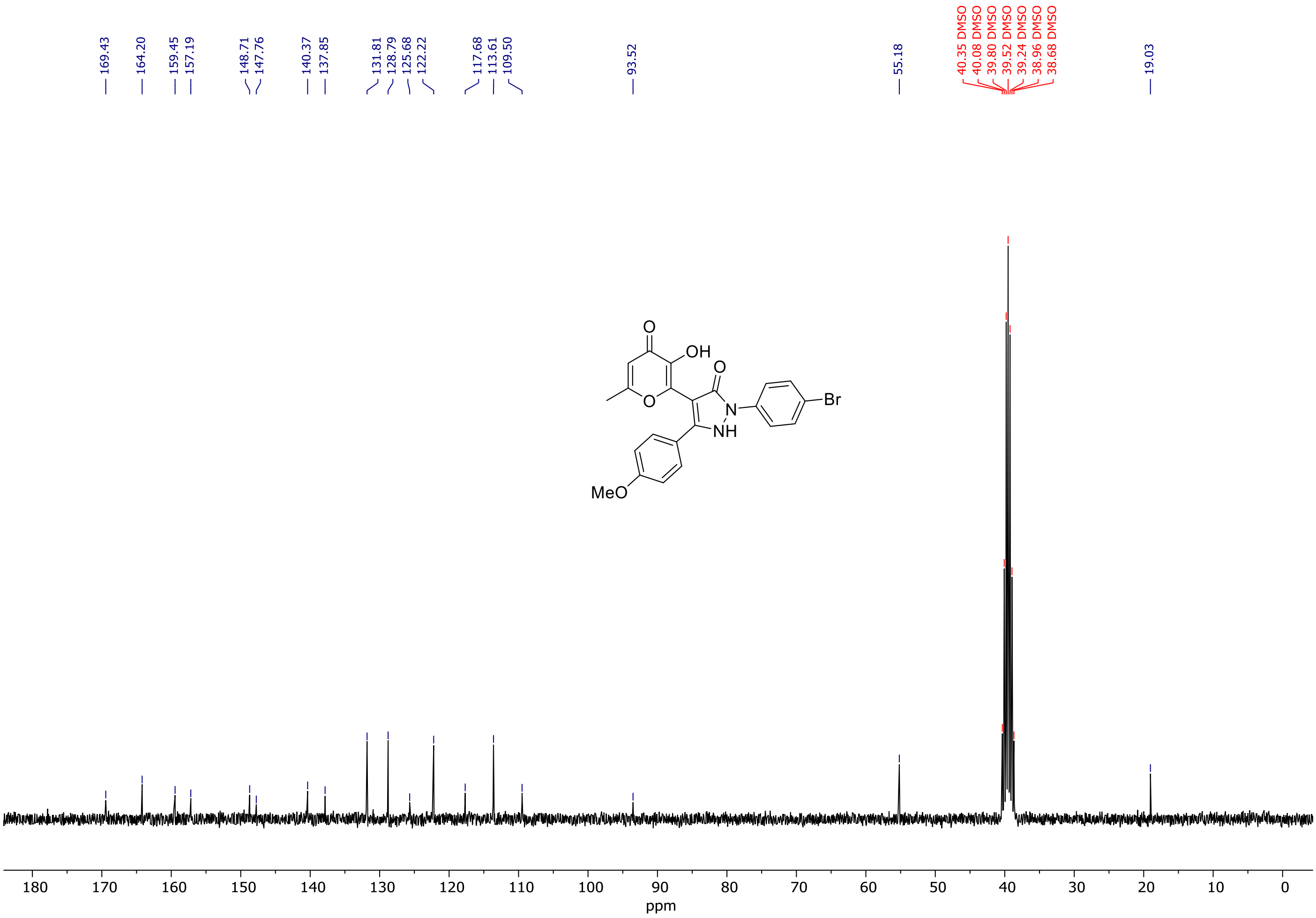

^{1}H NMR spectrum (300 MHz) of **8h** in DMSO-d_6

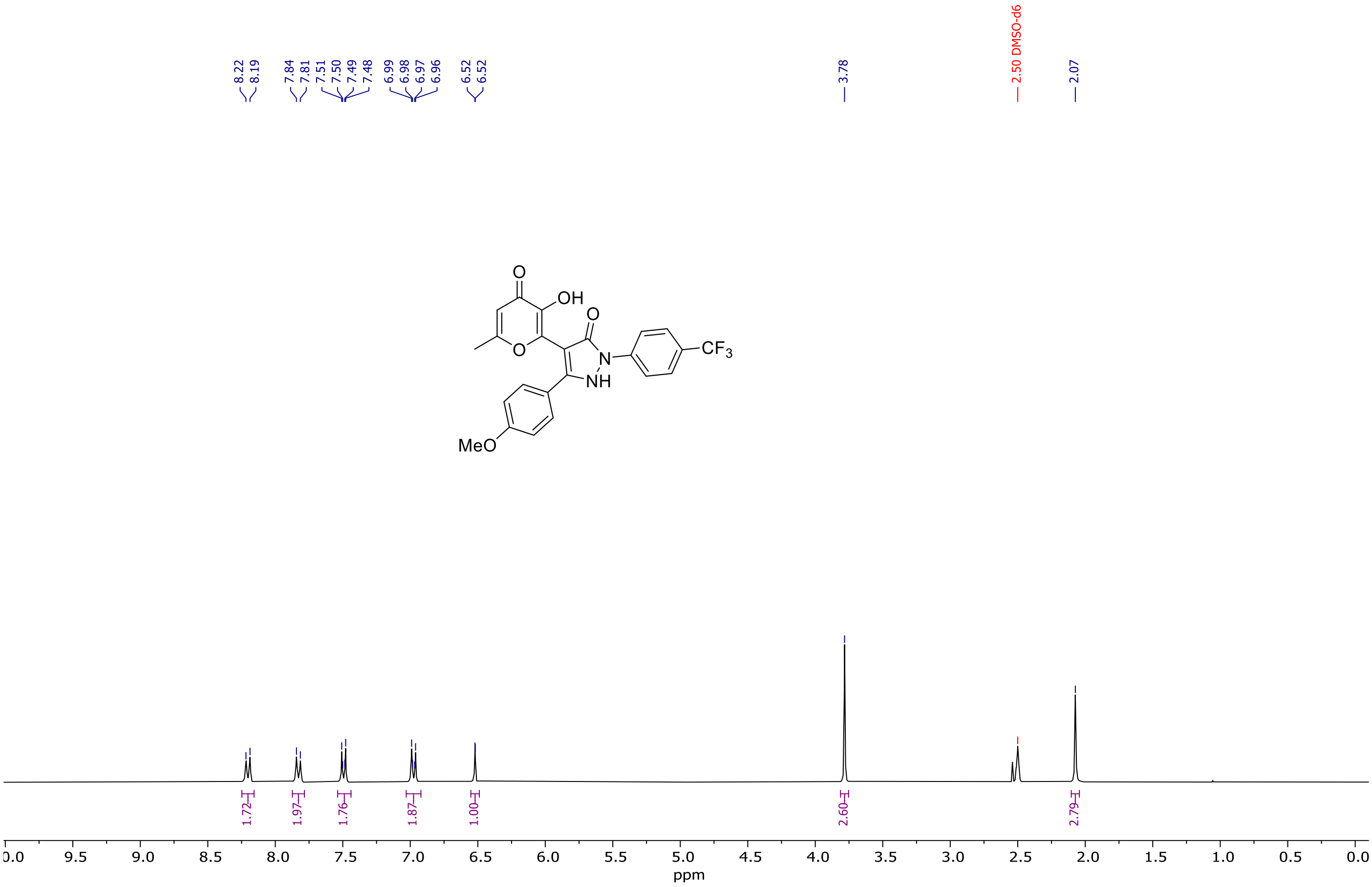

$^{13}C\{^{1}H\}$ NMR spectrum (75 MHz) of **8h** in DMSO-d_6

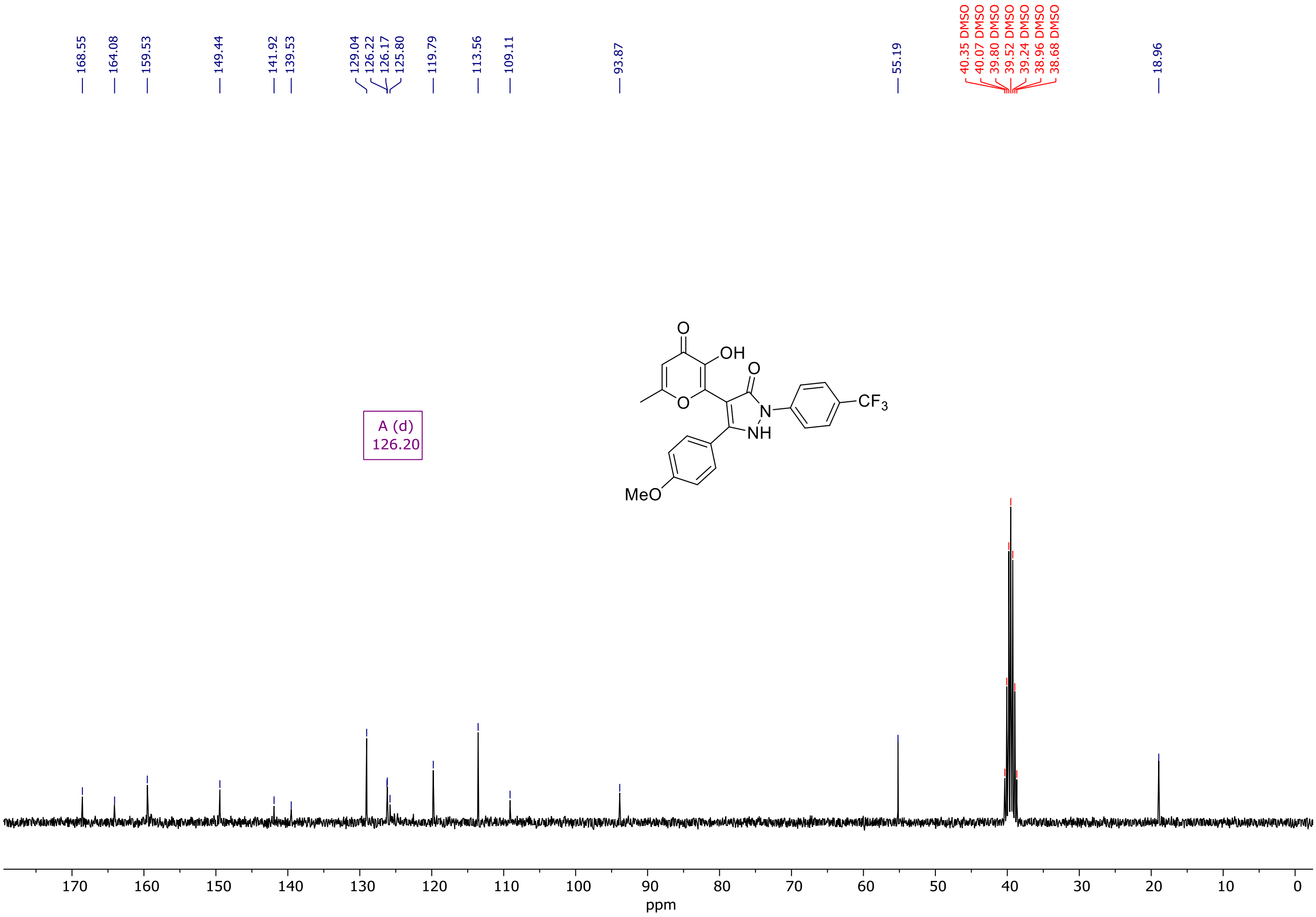

^{1}H NMR spectrum (300 MHz) of **8i** in DMSO-d_6

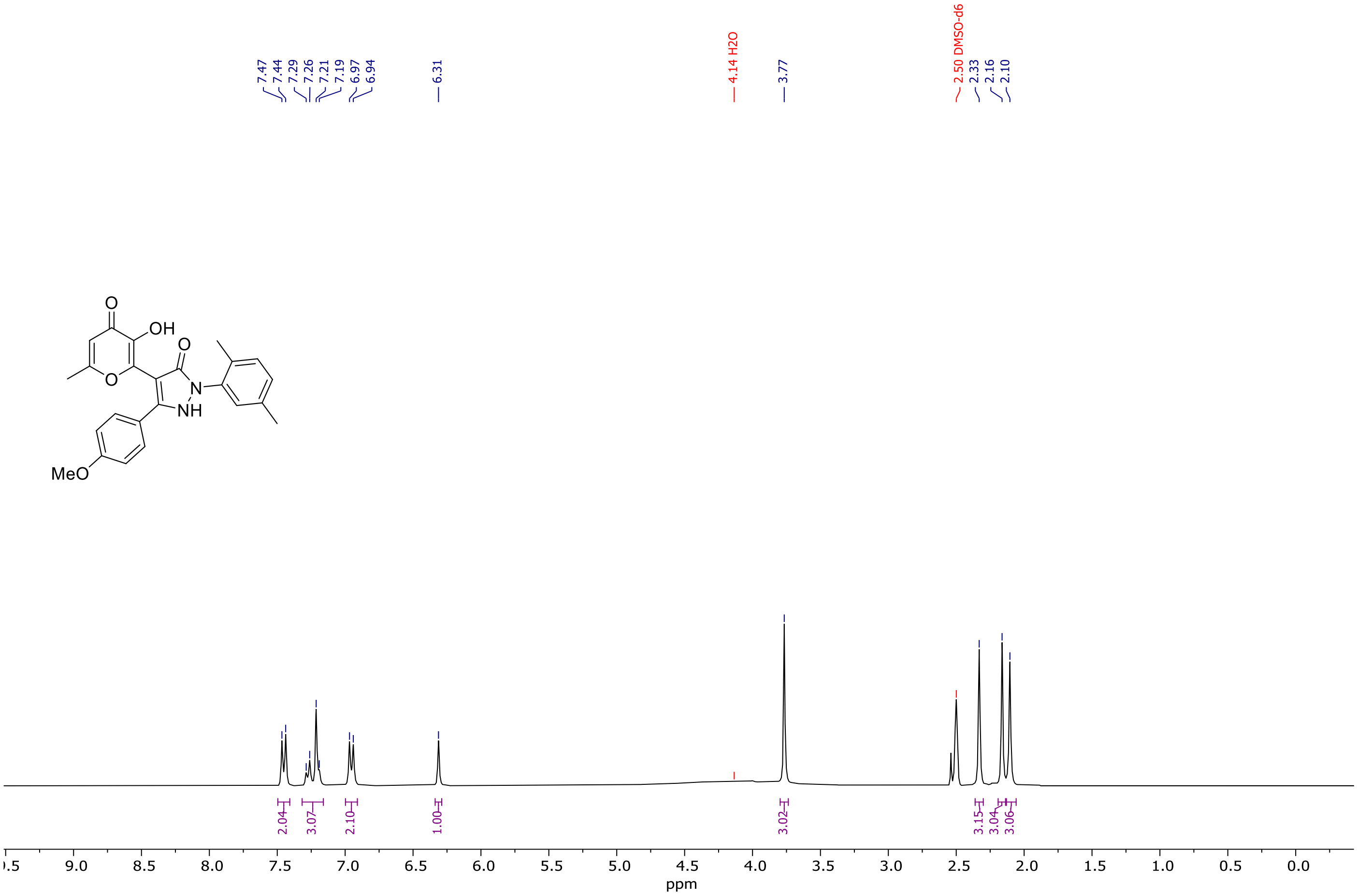

^{13}C {^{1}H} NMR spectrum (75 MHz) of **8i** in DMSO-d_6

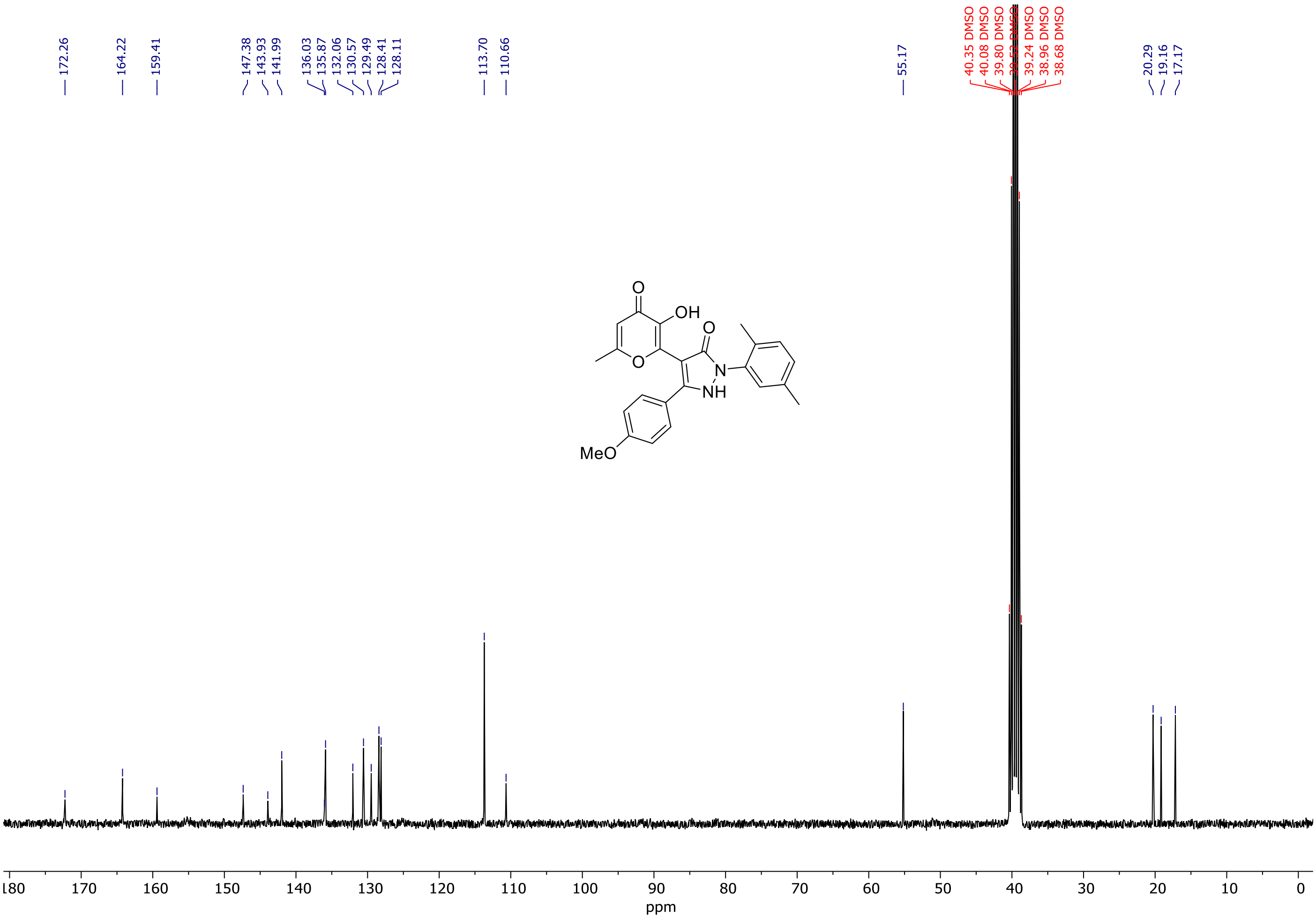

^1H NMR spectrum (300 MHz) of **8j** in DMSO-d_6

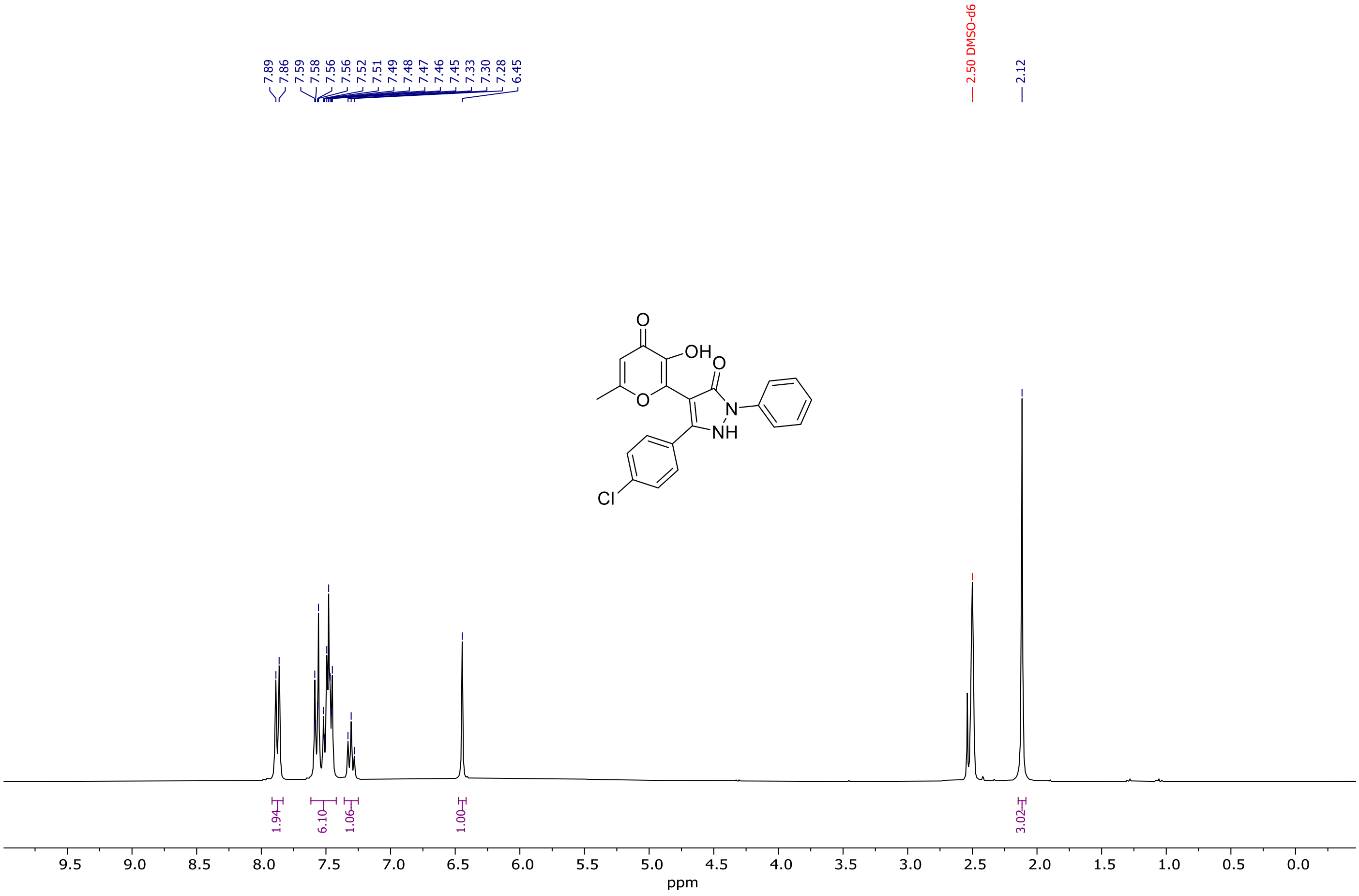

^{13}C {^1H} NMR spectrum (75 MHz) of **8j** in DMSO-d_6

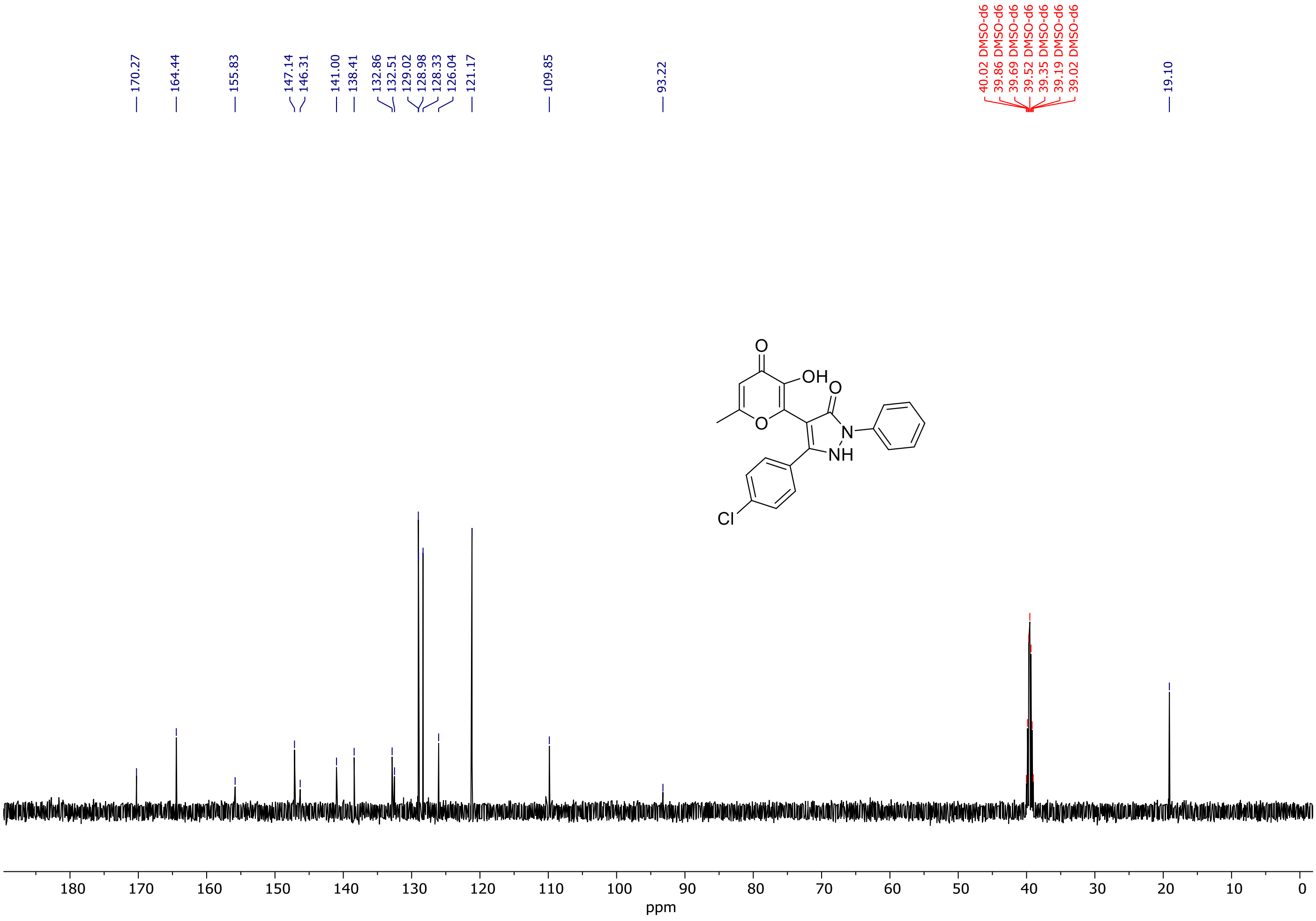

^{1}H NMR spectrum (300 MHz) of **8k** in DMSO-d_6

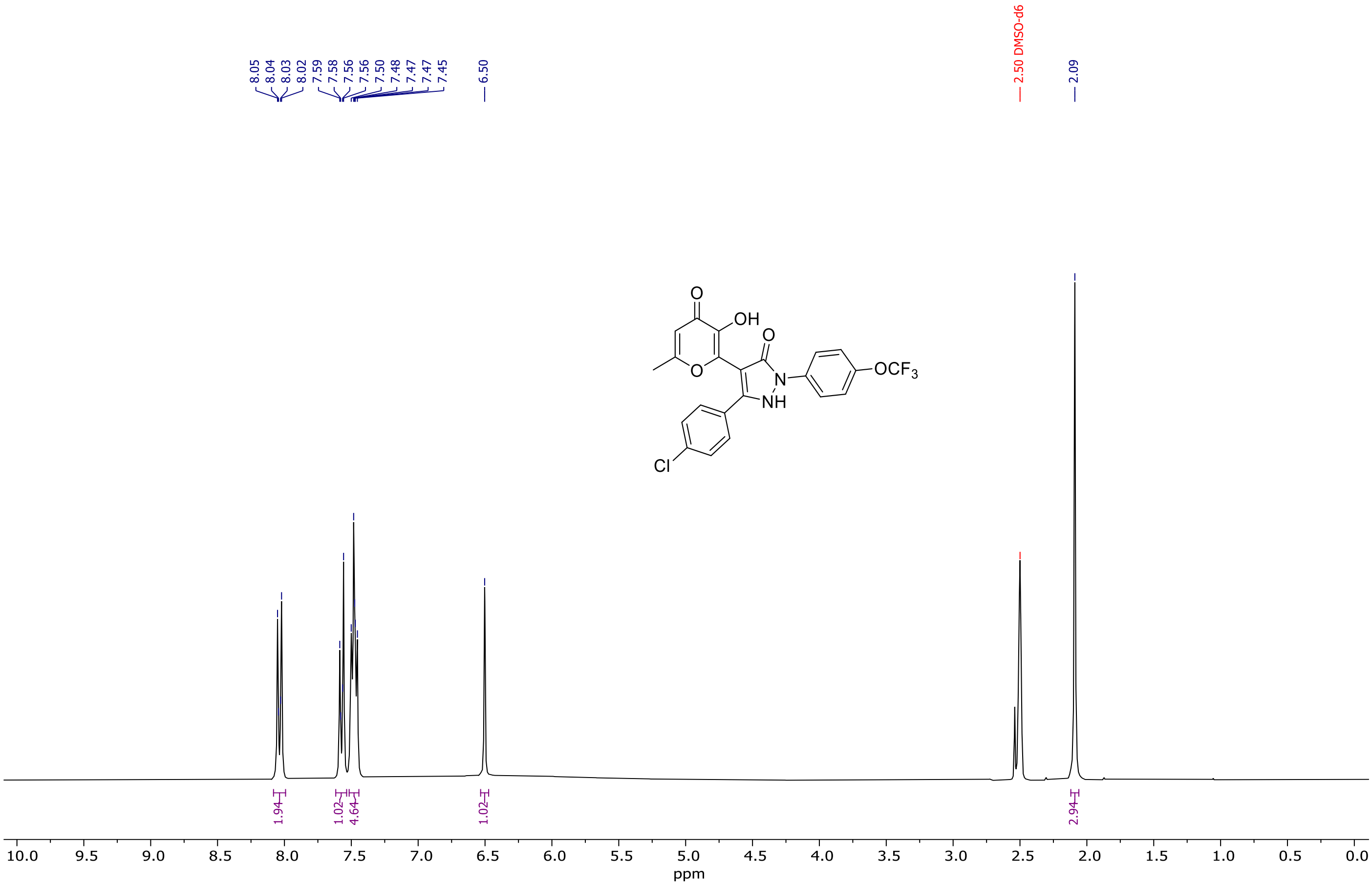

^{13}C {^{1}H} NMR spectrum (75 MHz) of **8k** in DMSO-d_6

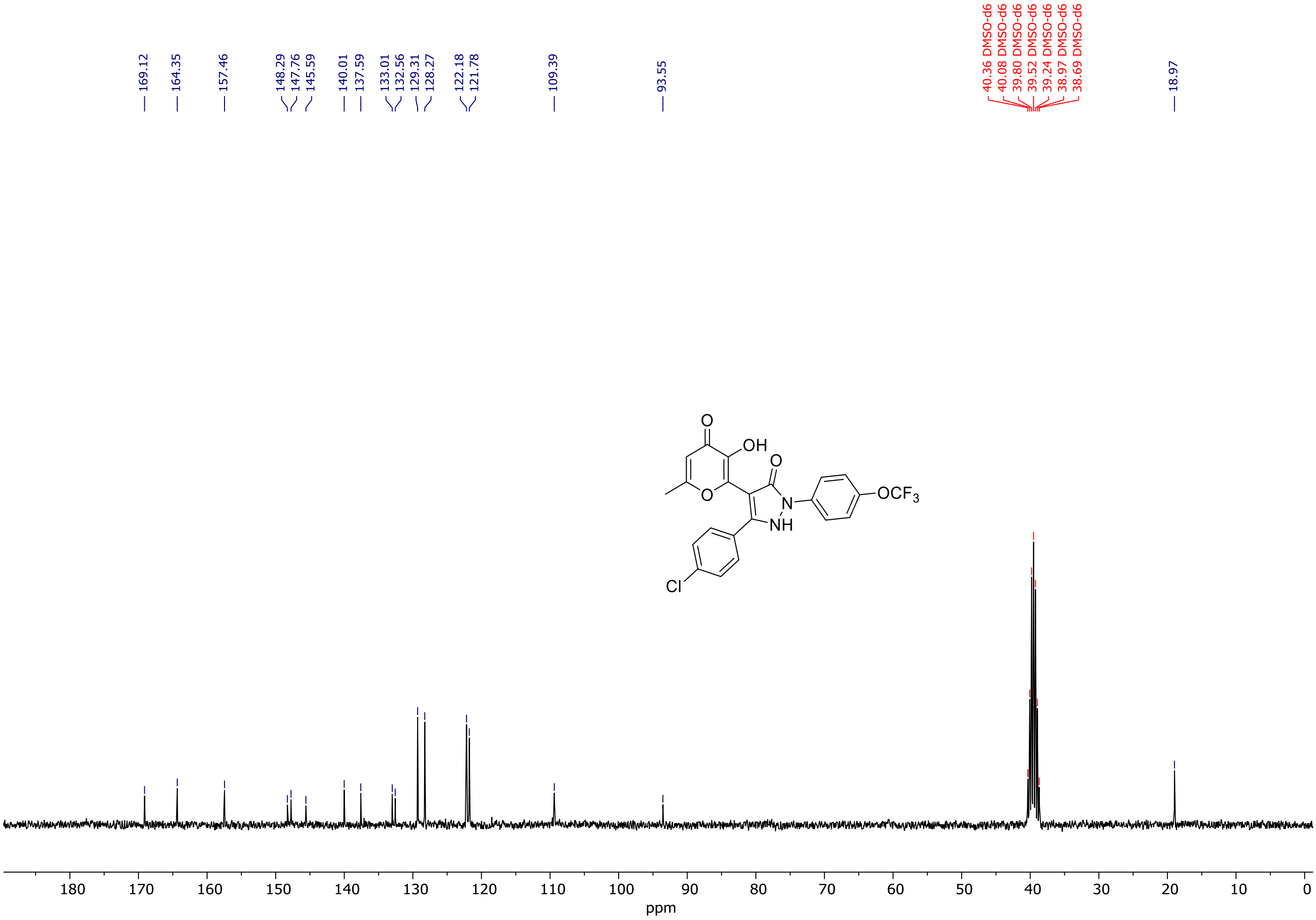

^{1}H NMR spectrum (300 MHz) of **8l** in DMSO-d_6

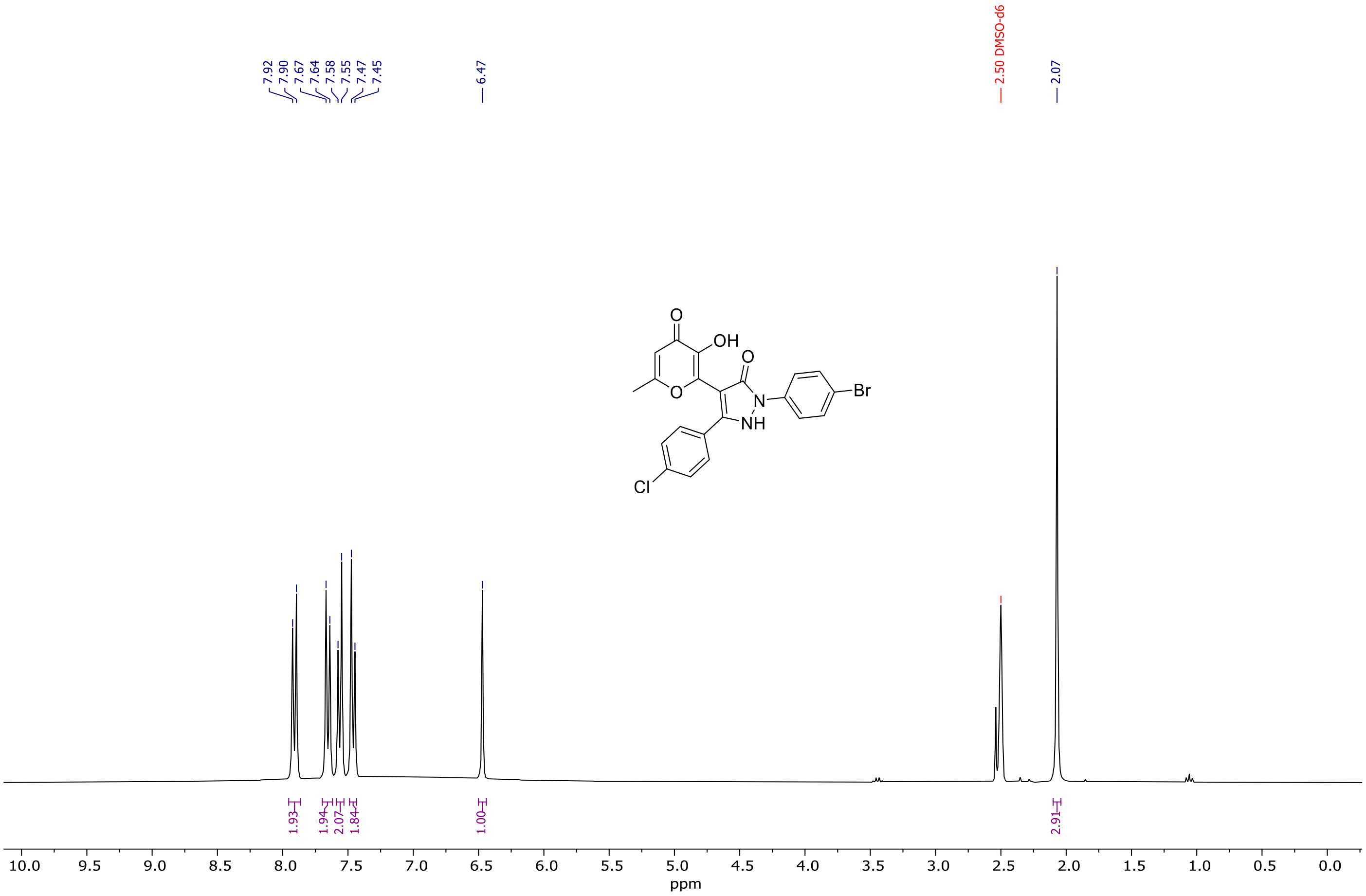

^{13}C {^{1}H} NMR spectrum (75 MHz) of **8l** in DMSO-d_6

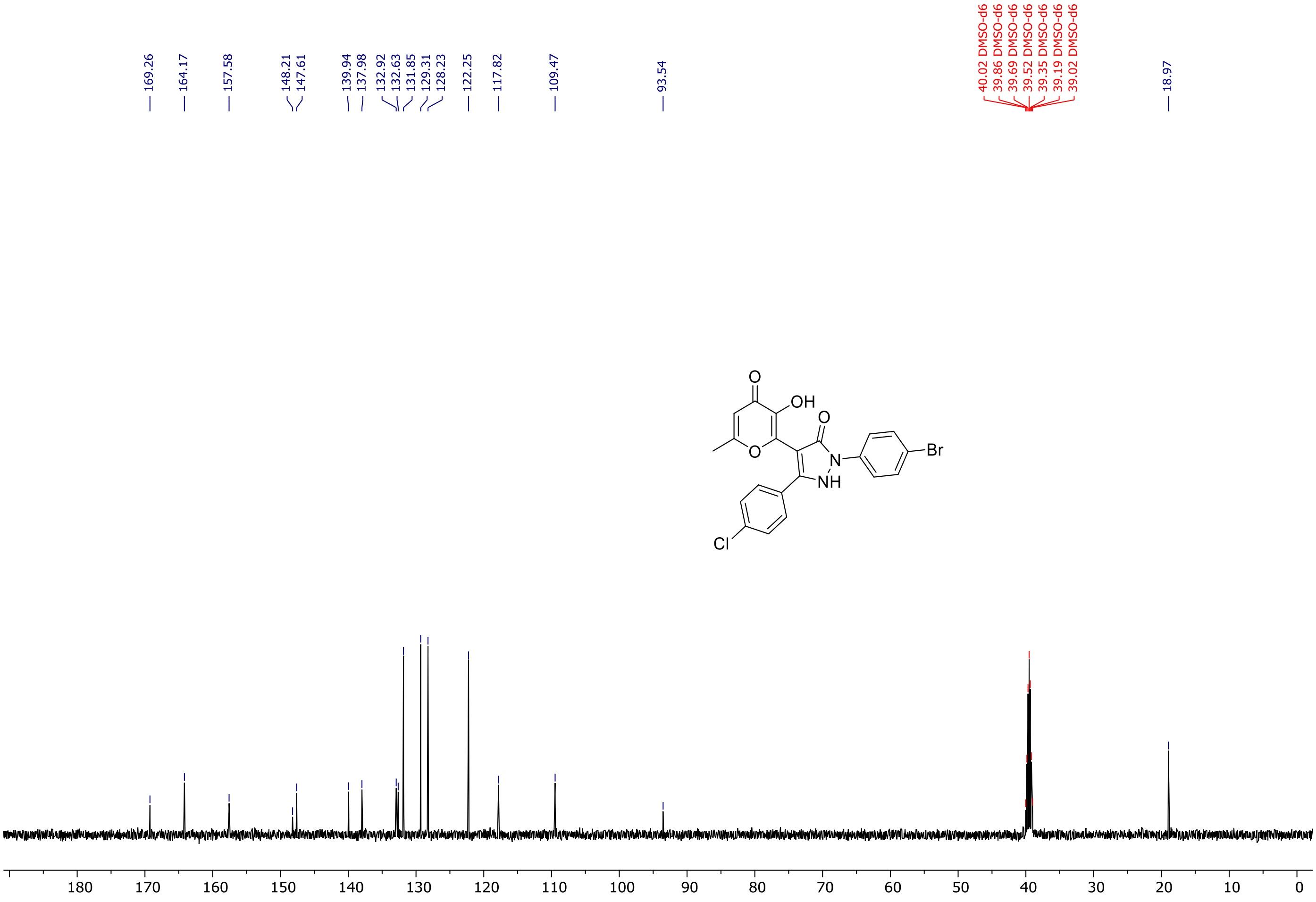

^{1}H NMR spectrum (300 MHz) of **8m** in DMSO-d_6

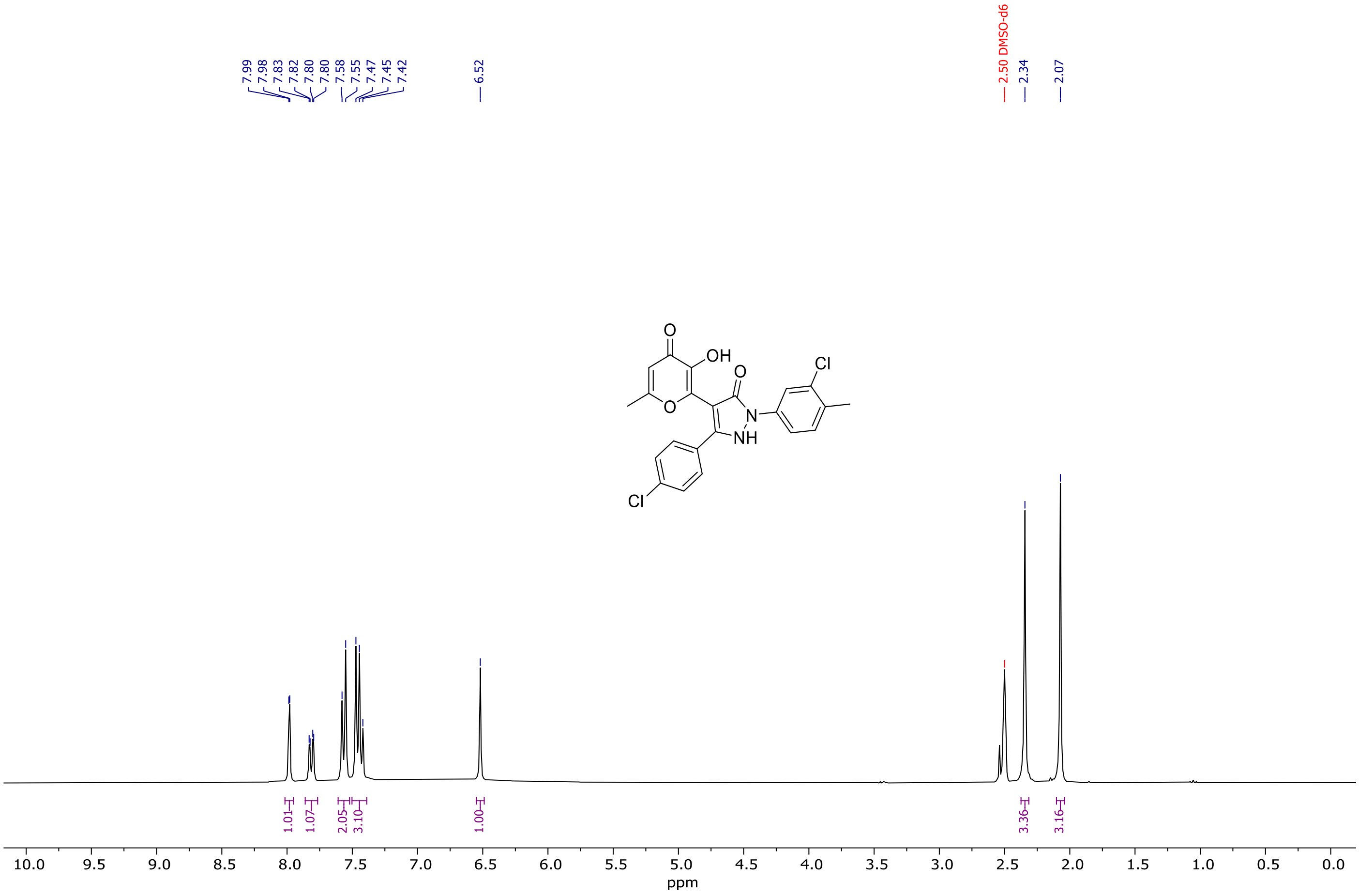

^{13}C {^{1}H} NMR spectrum (75 MHz) of **8m** in DMSO-d_6

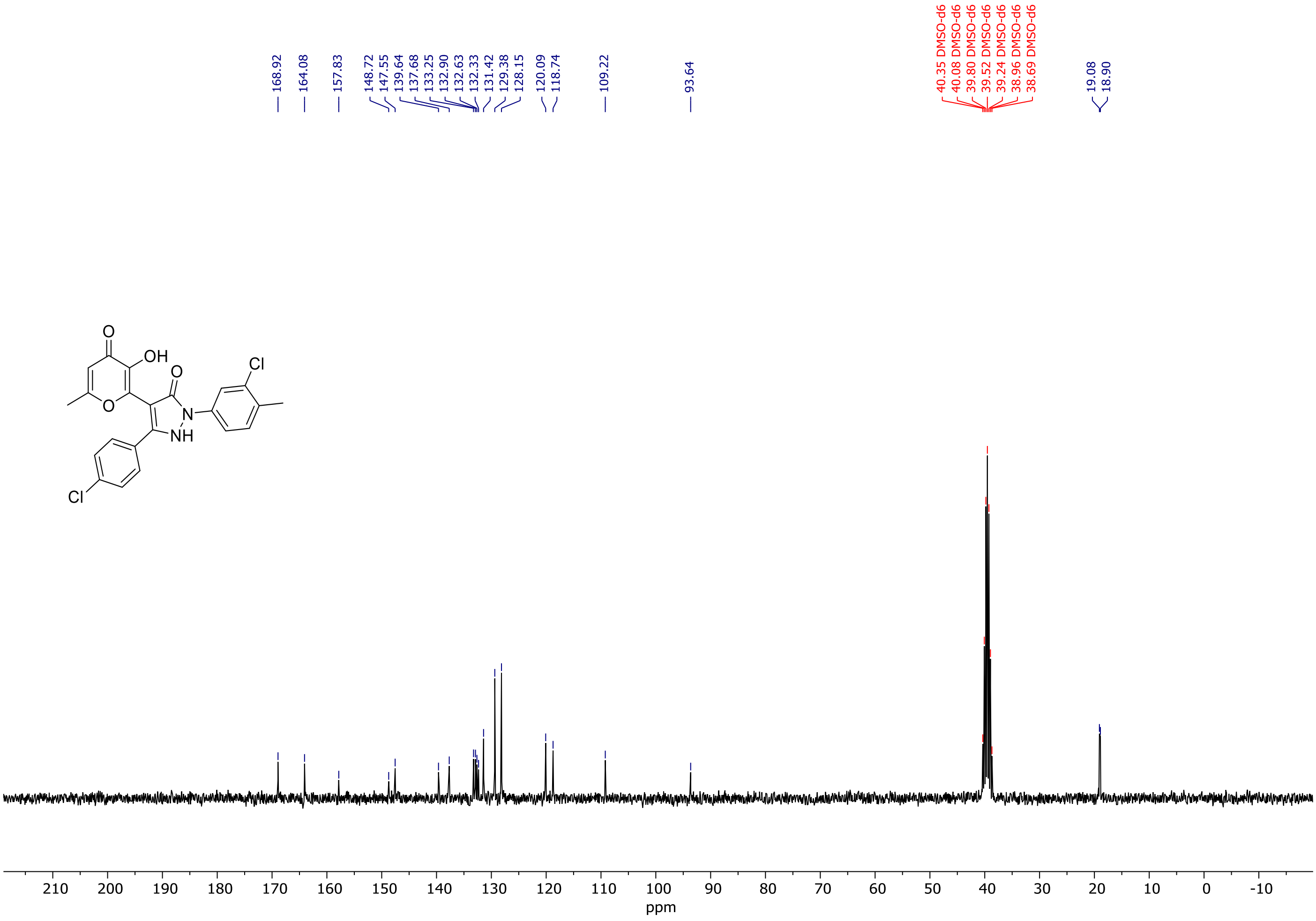

^1H NMR spectrum (300 MHz) of **8n** in DMSO-d_6

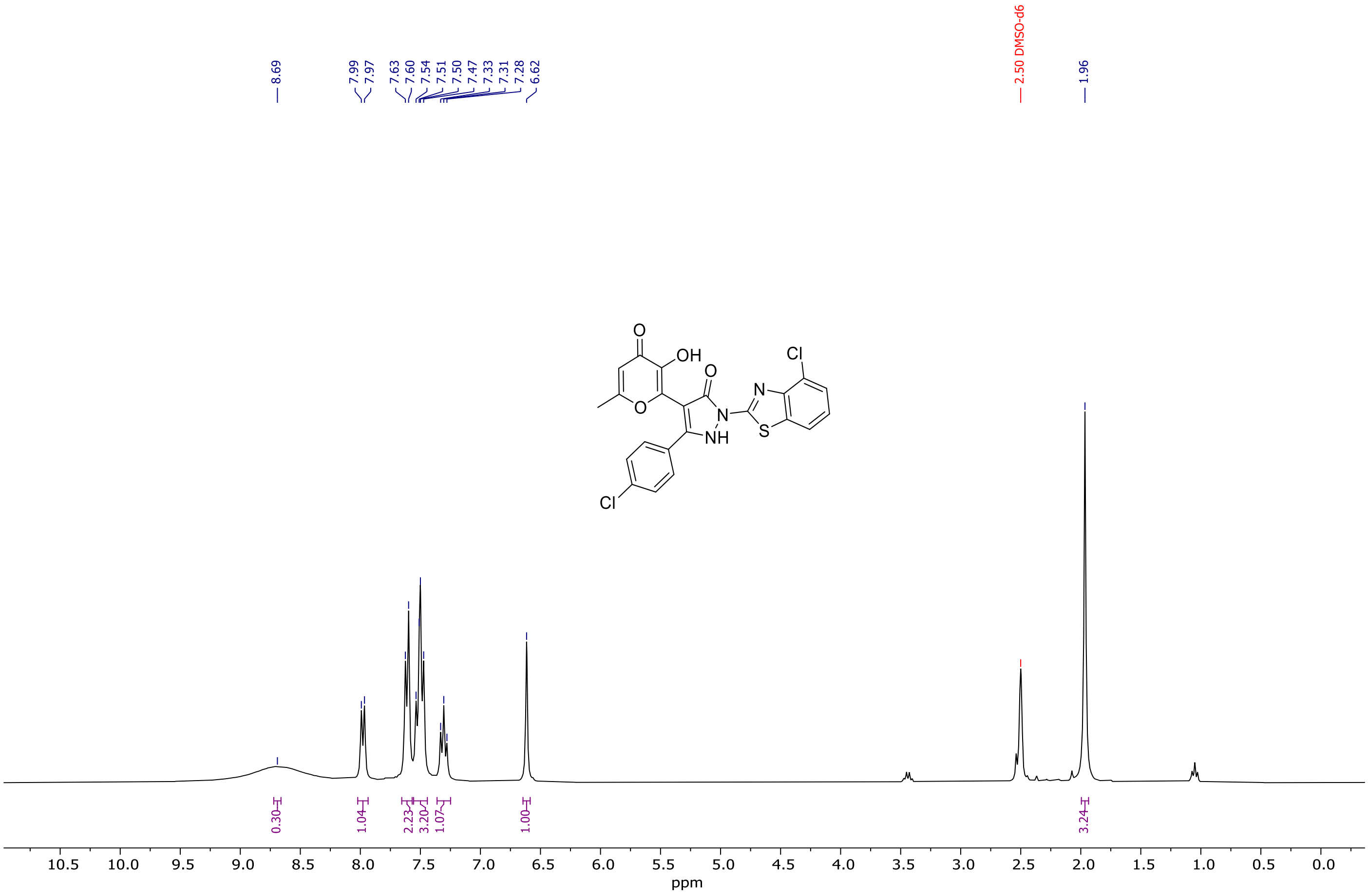

^{13}C {^1H} NMR spectrum (75 MHz) of **8n** in DMSO-d_6

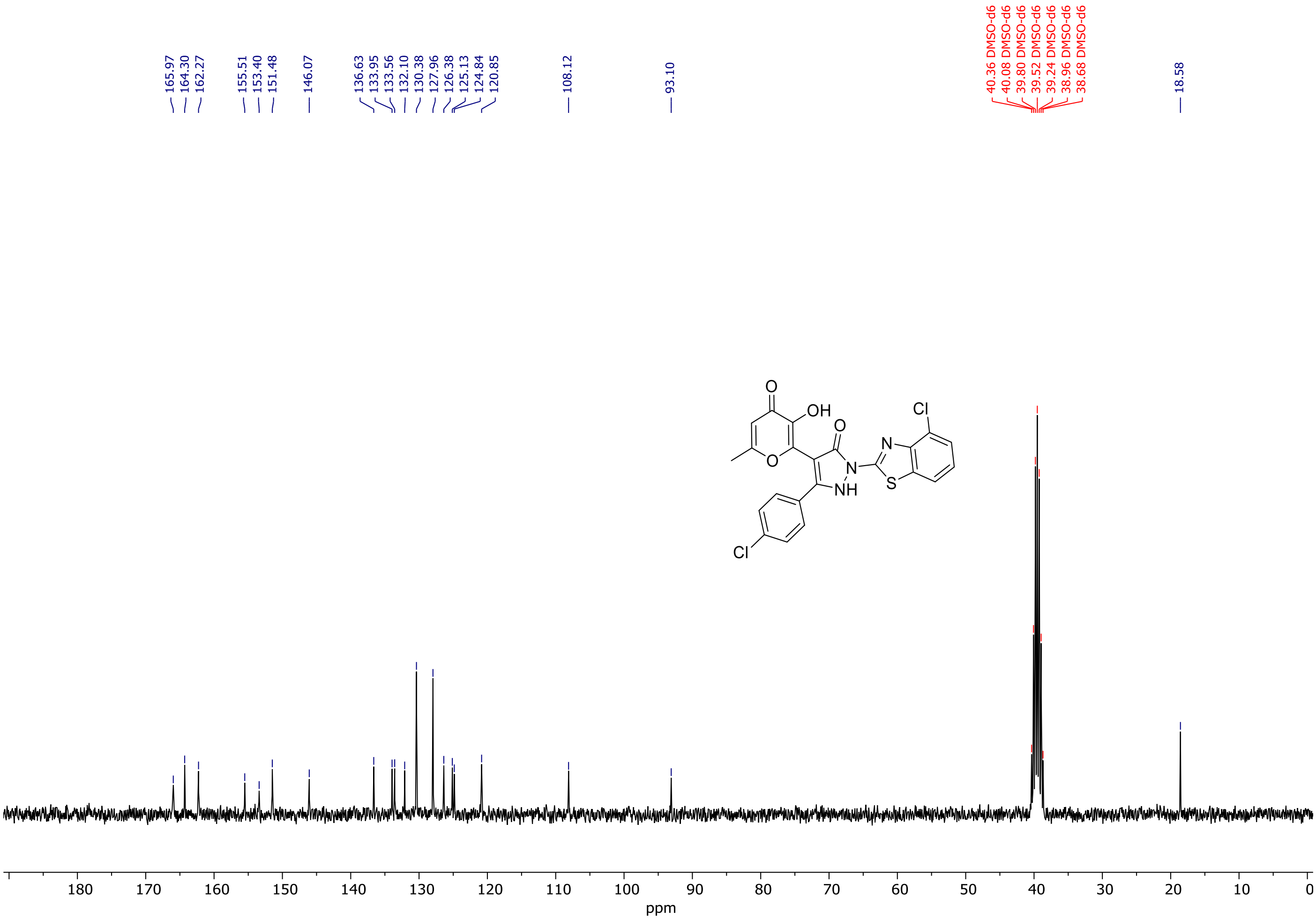

^{1}H NMR spectrum (300 MHz) of **8o** in DMSO-d_6

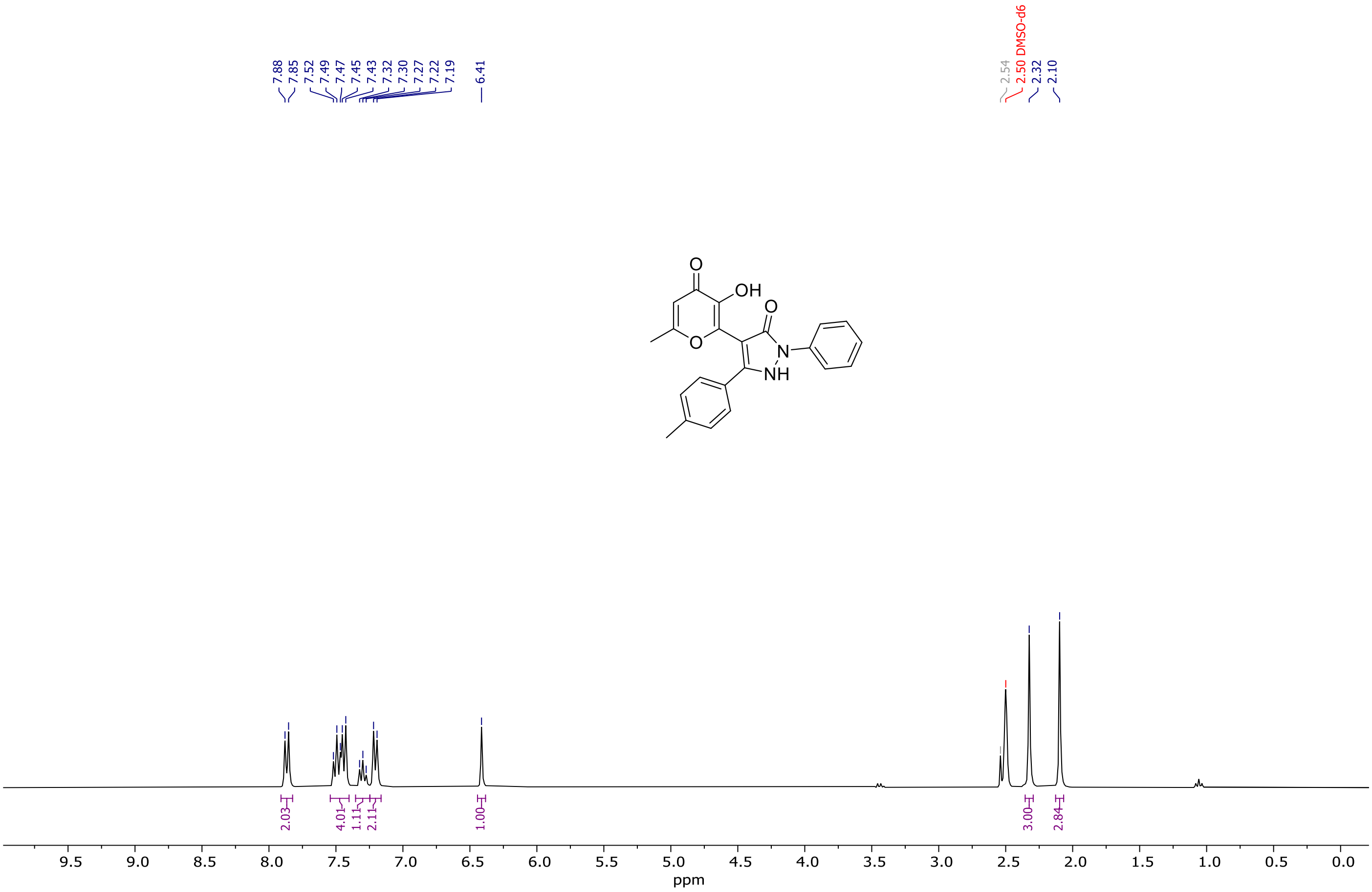

^{13}C {^{1}H} NMR spectrum (75 MHz) of **8o** in DMSO-d_6

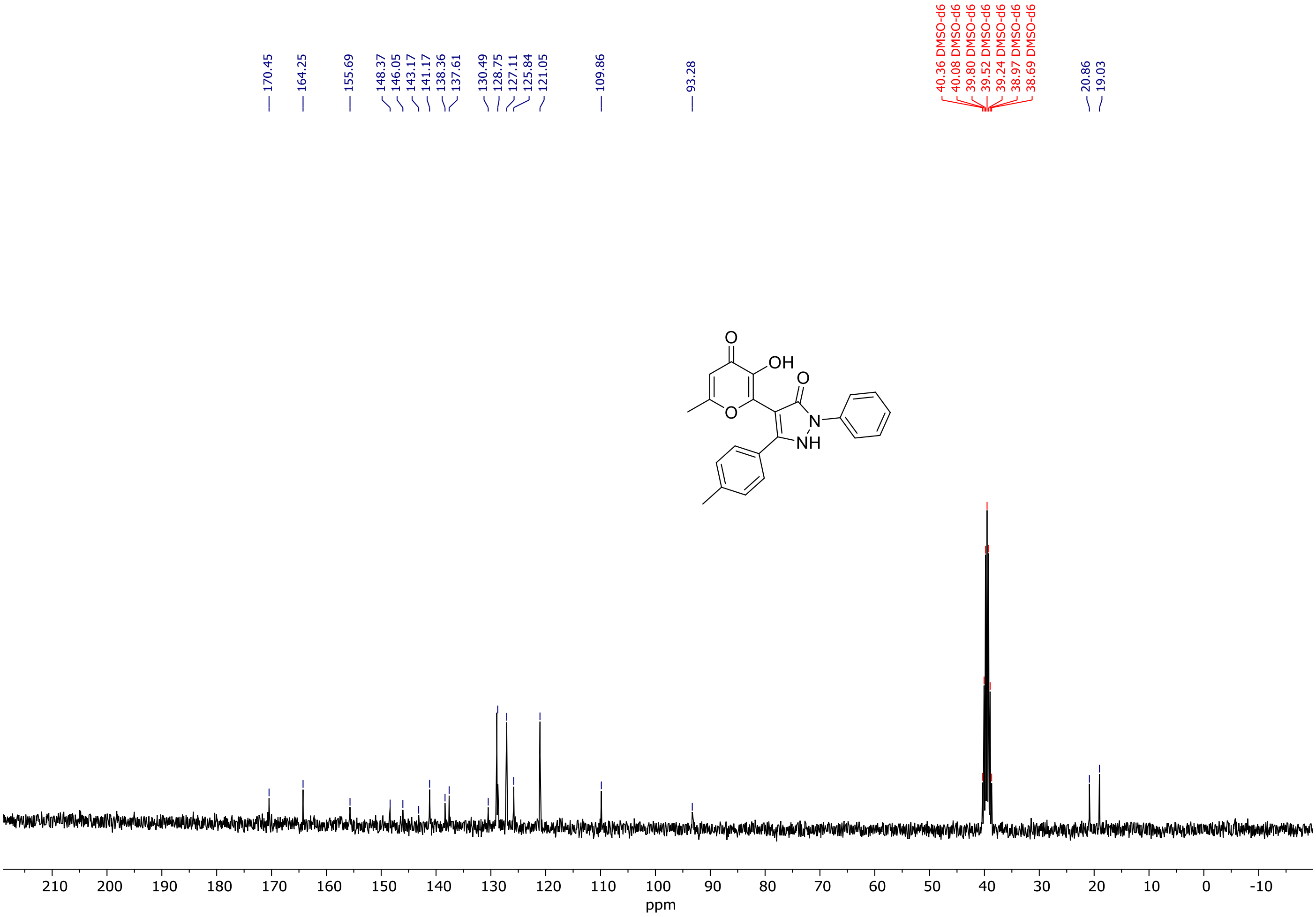

^{1}H NMR spectrum (300 MHz) of **8p** in DMSO-d_6

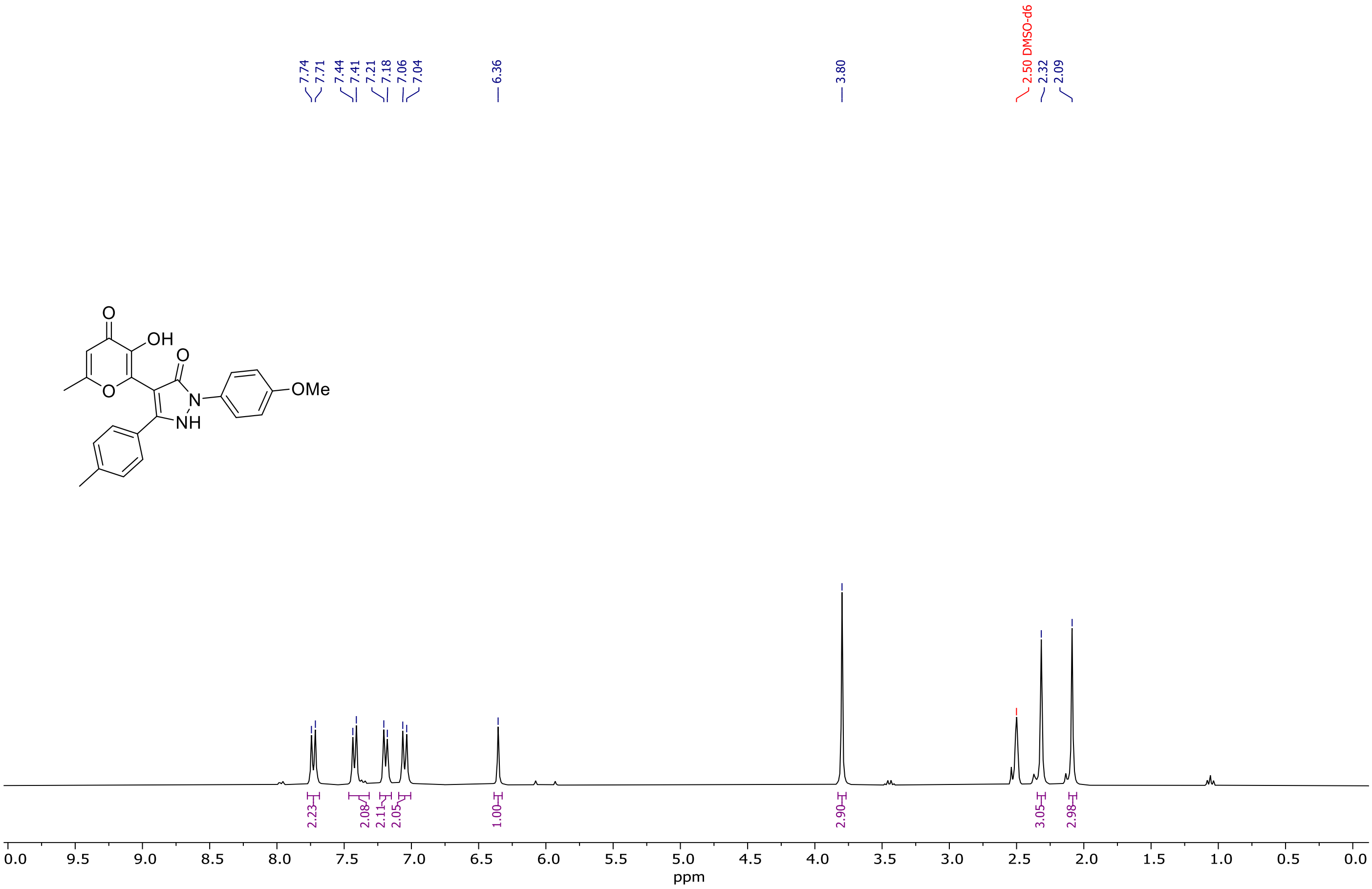

^{13}C {^{1}H} NMR spectrum (75 MHz) of **8p** in DMSO-d_6

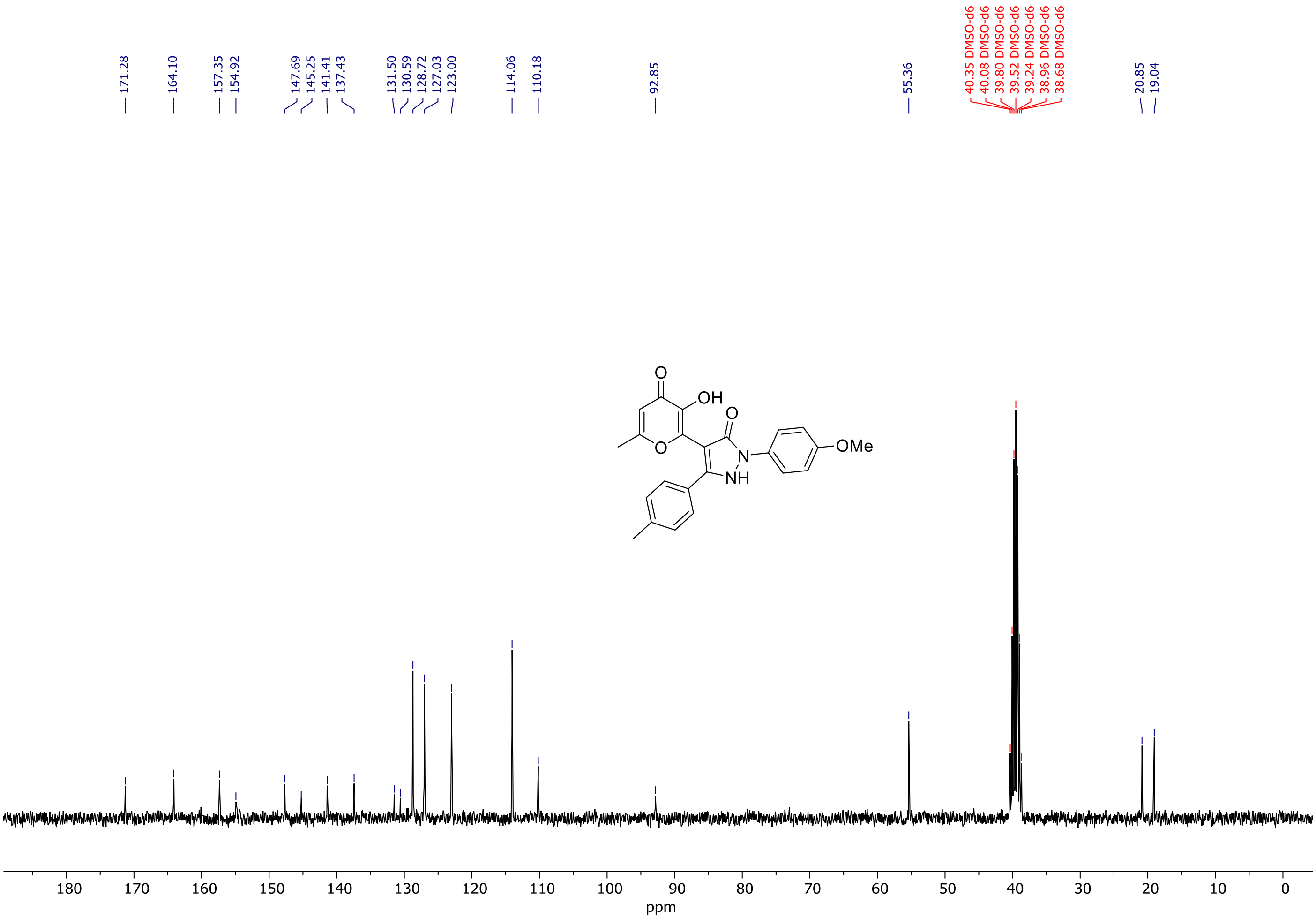

^{1}H NMR spectrum (300 MHz) of **8q** in DMSO-d_6

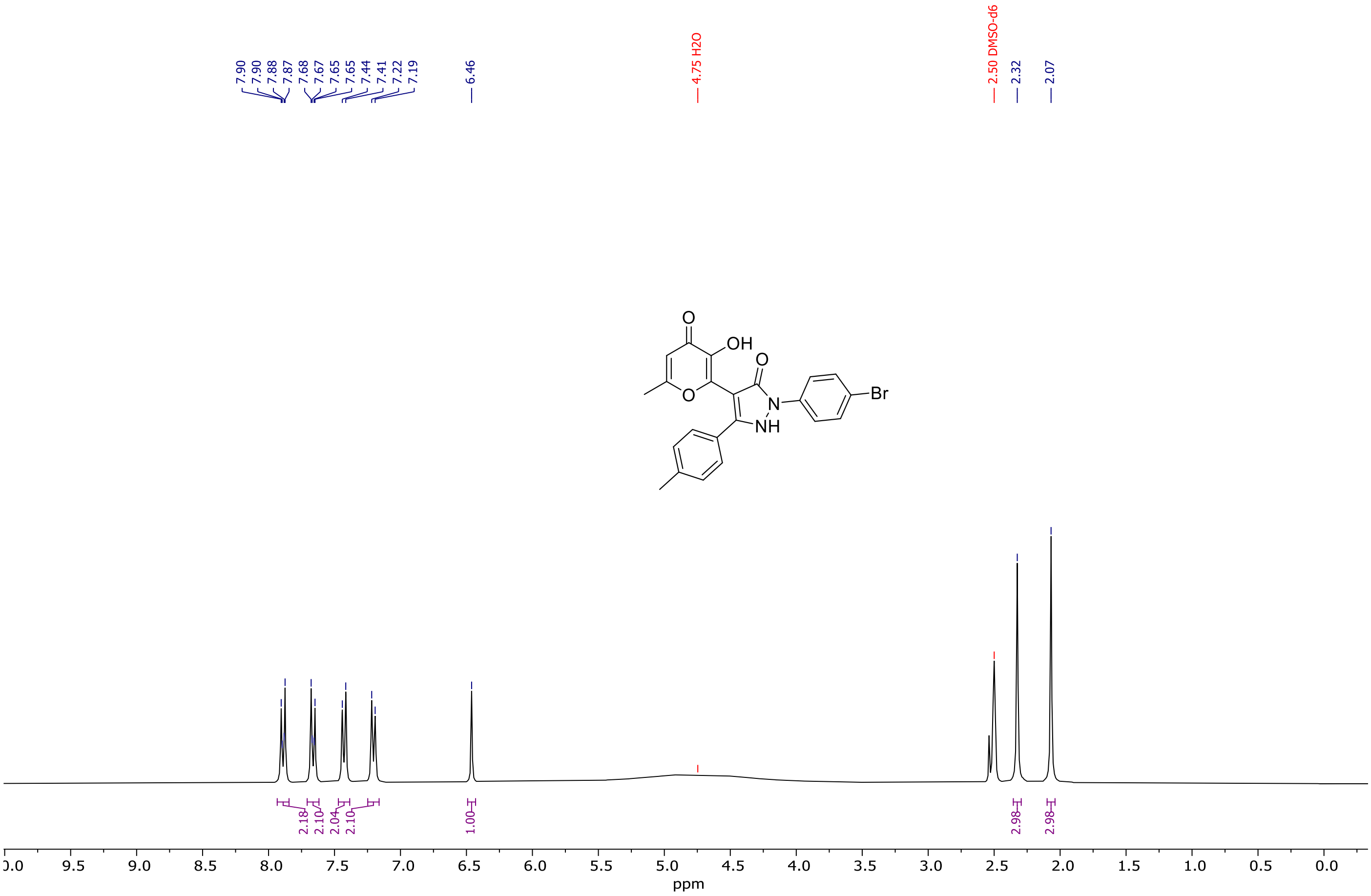

^{13}C {^{1}H} NMR spectrum (75 MHz) of **8q** in DMSO-d_6

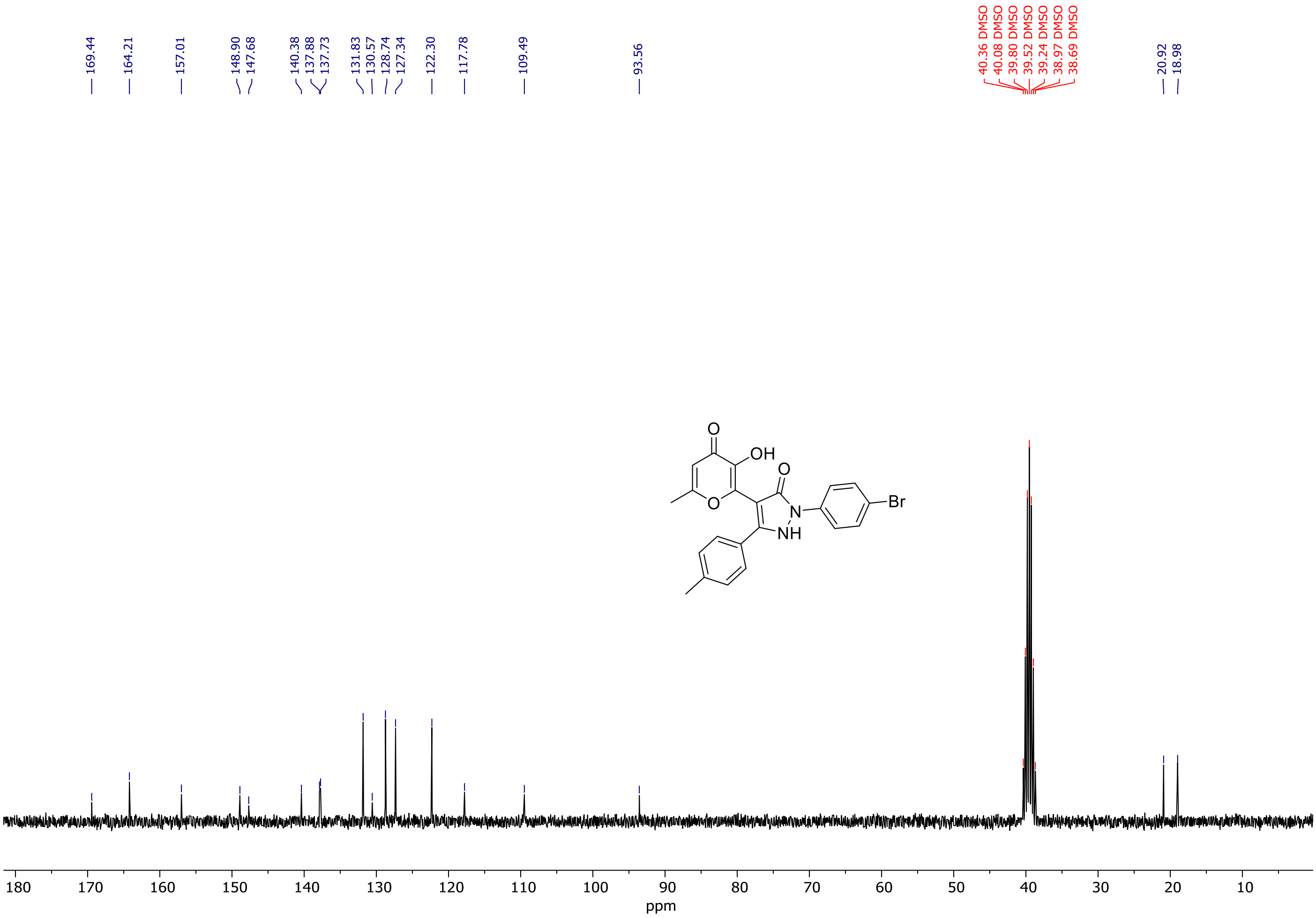

^{1}H NMR spectrum (300 MHz) of **8r** in DMSO-d_6

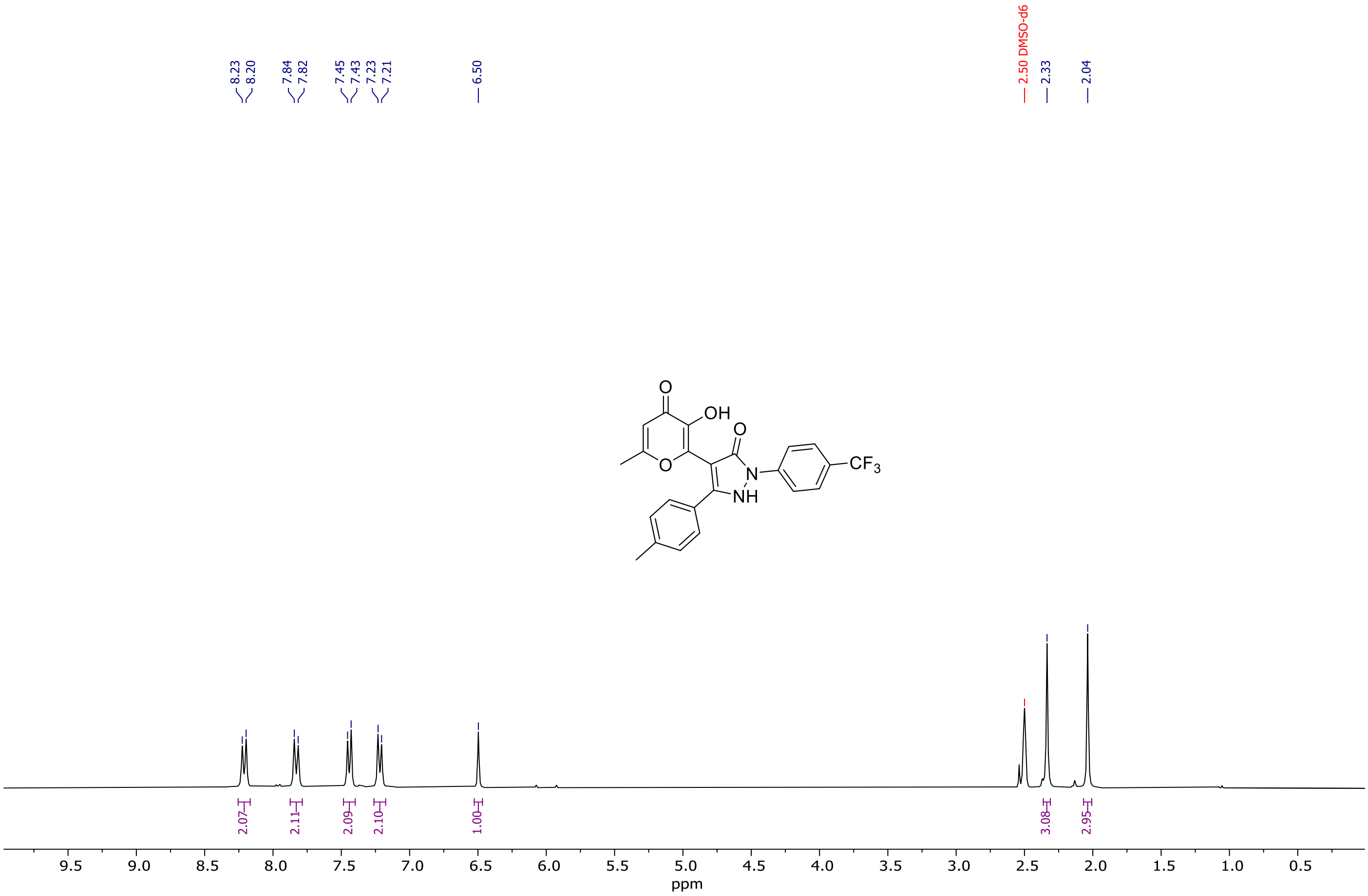

^{13}C {^{1}H} NMR spectrum (75 MHz) of **8r** in DMSO-d_6

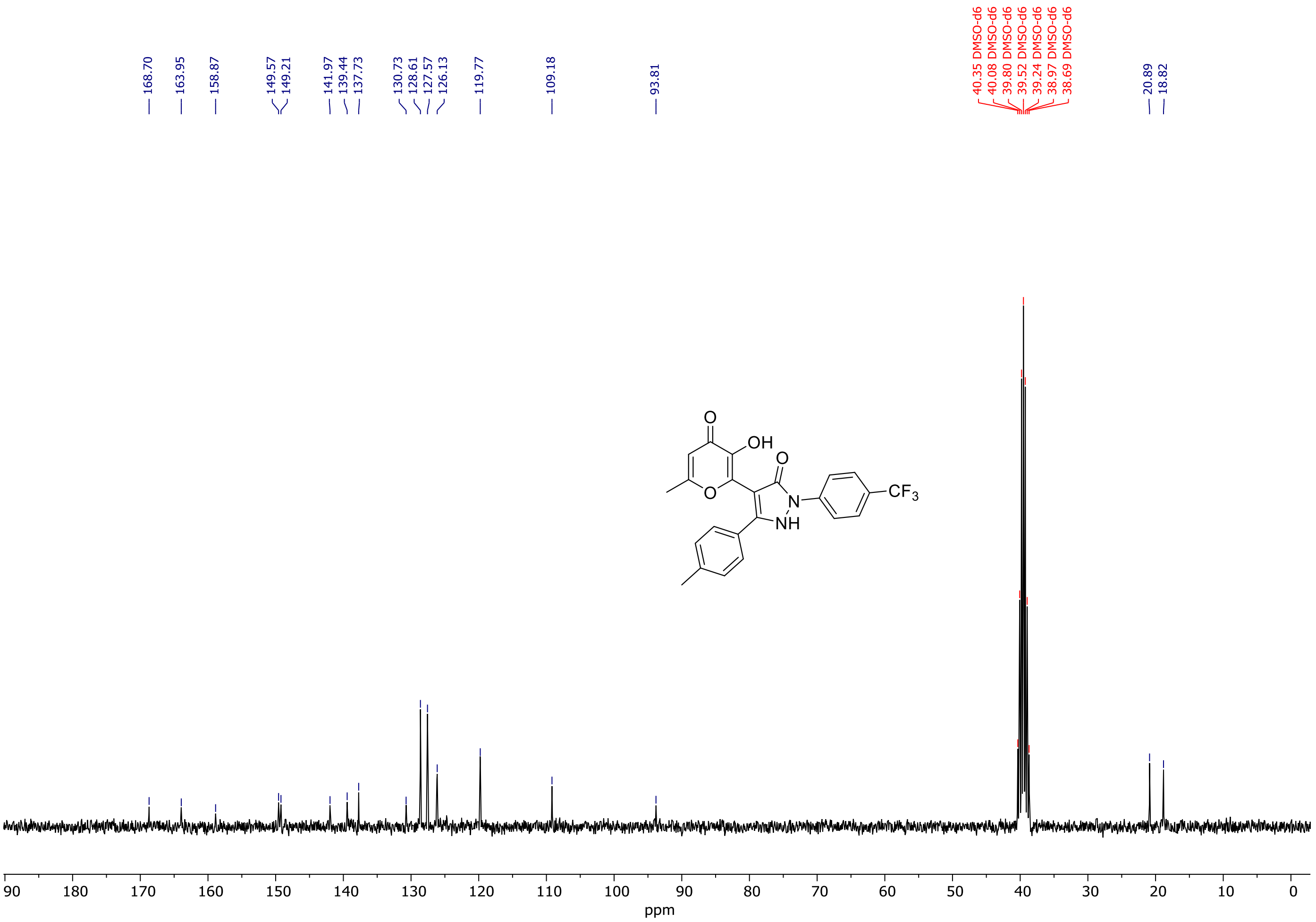

^{1}H NMR spectrum (300 MHz) of **8s** in DMSO-d_6

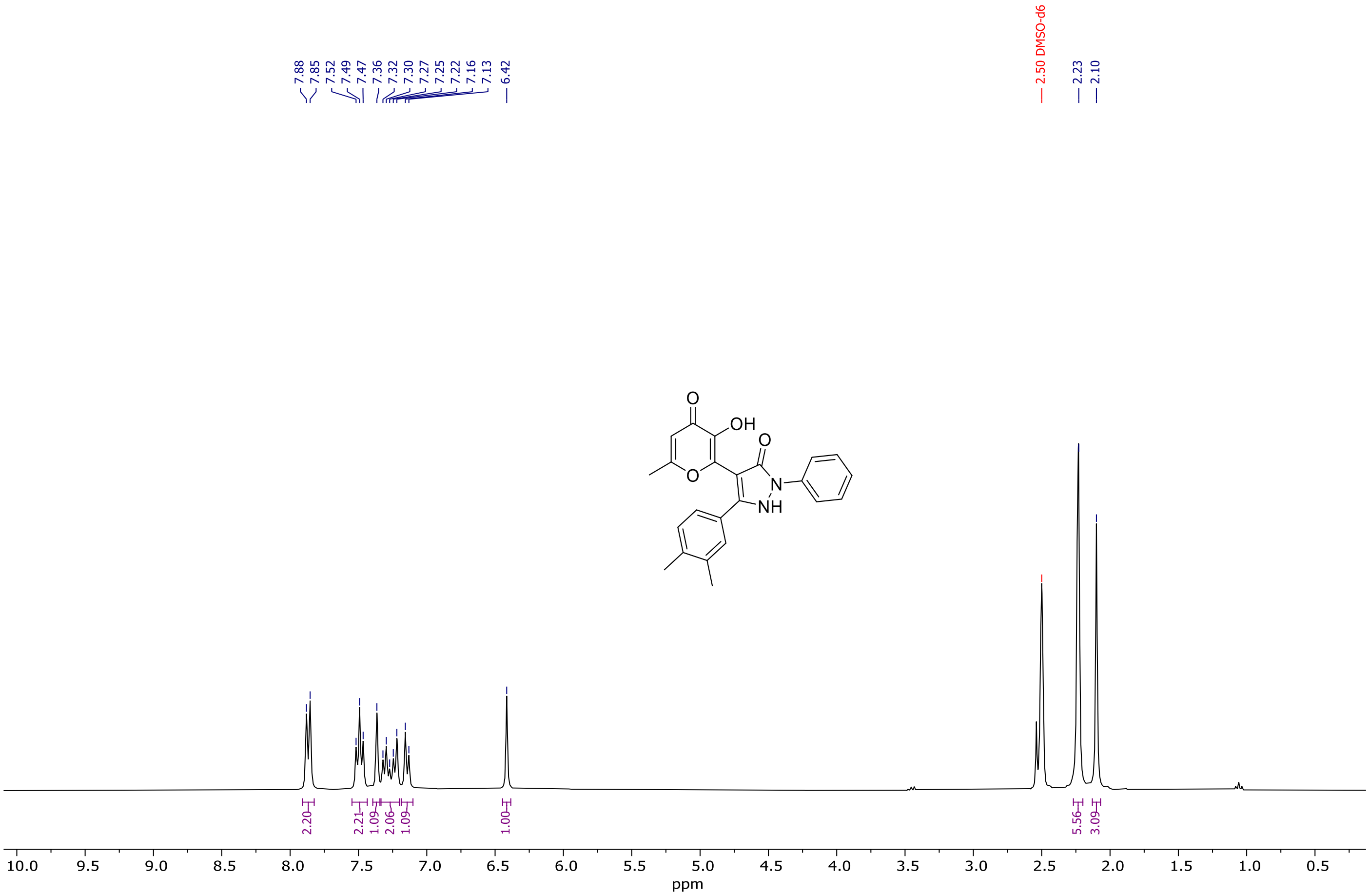

^{13}C {^{1}H} NMR spectrum (75 MHz) of **8s** in DMSO-d_6

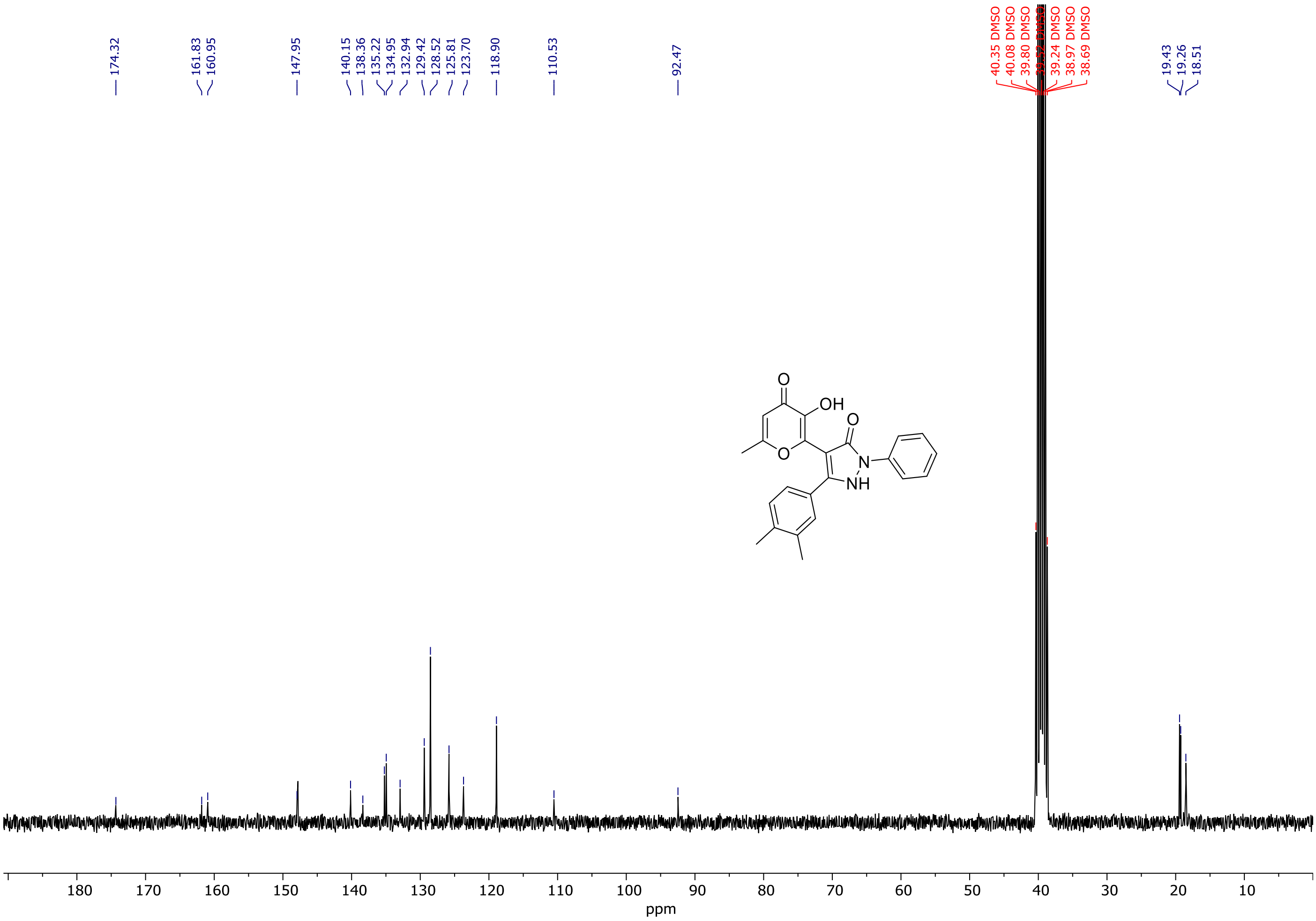

10. NMR ^{1}H and ^{13}C spectra for compounds 10

^{1}H NMR spectrum (300 MHz) of **10a** in DMSO-d_6

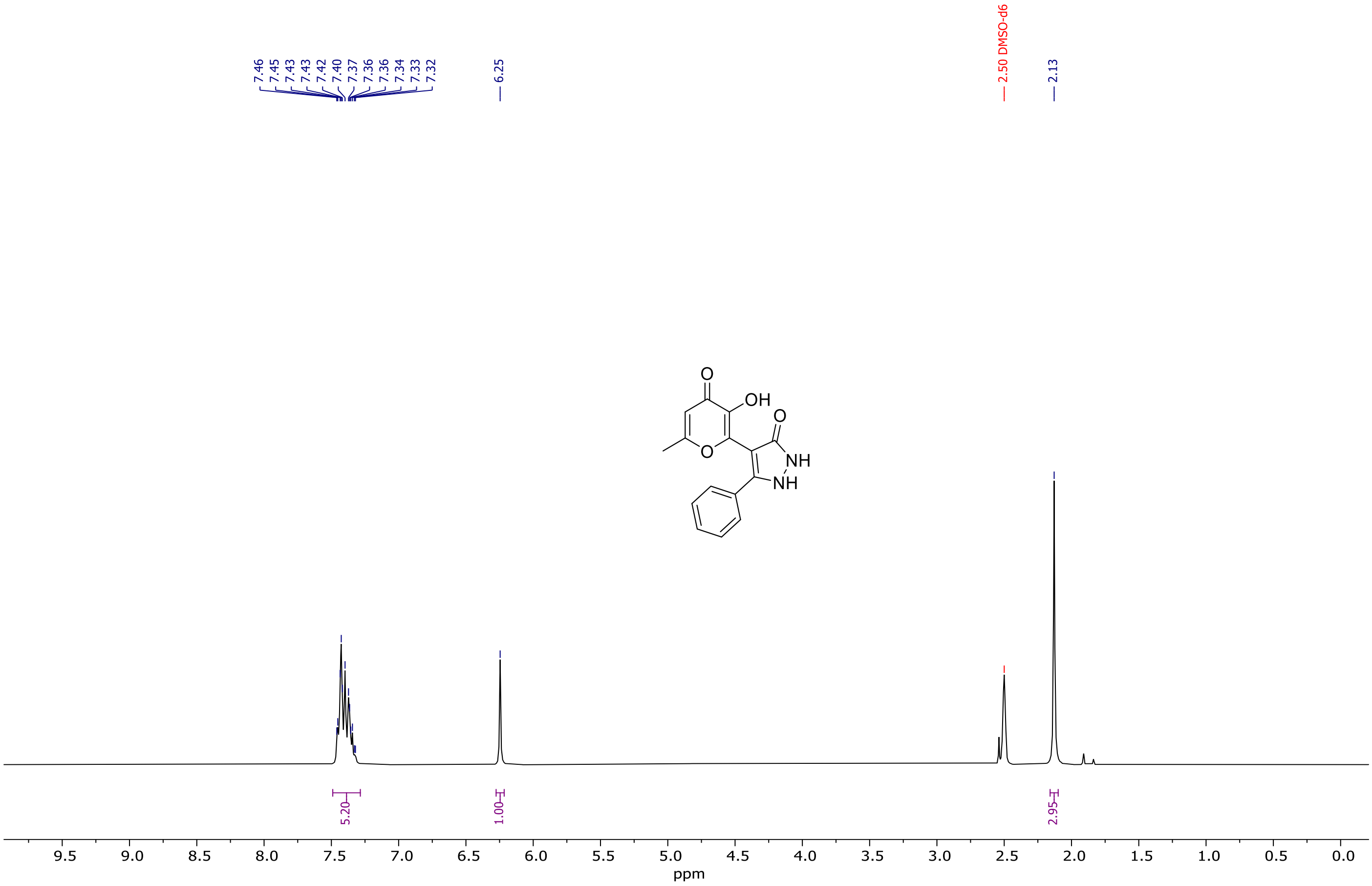

^{13}C {^{1}H} NMR spectrum (75 MHz) of **10a** in DMSO-d_6

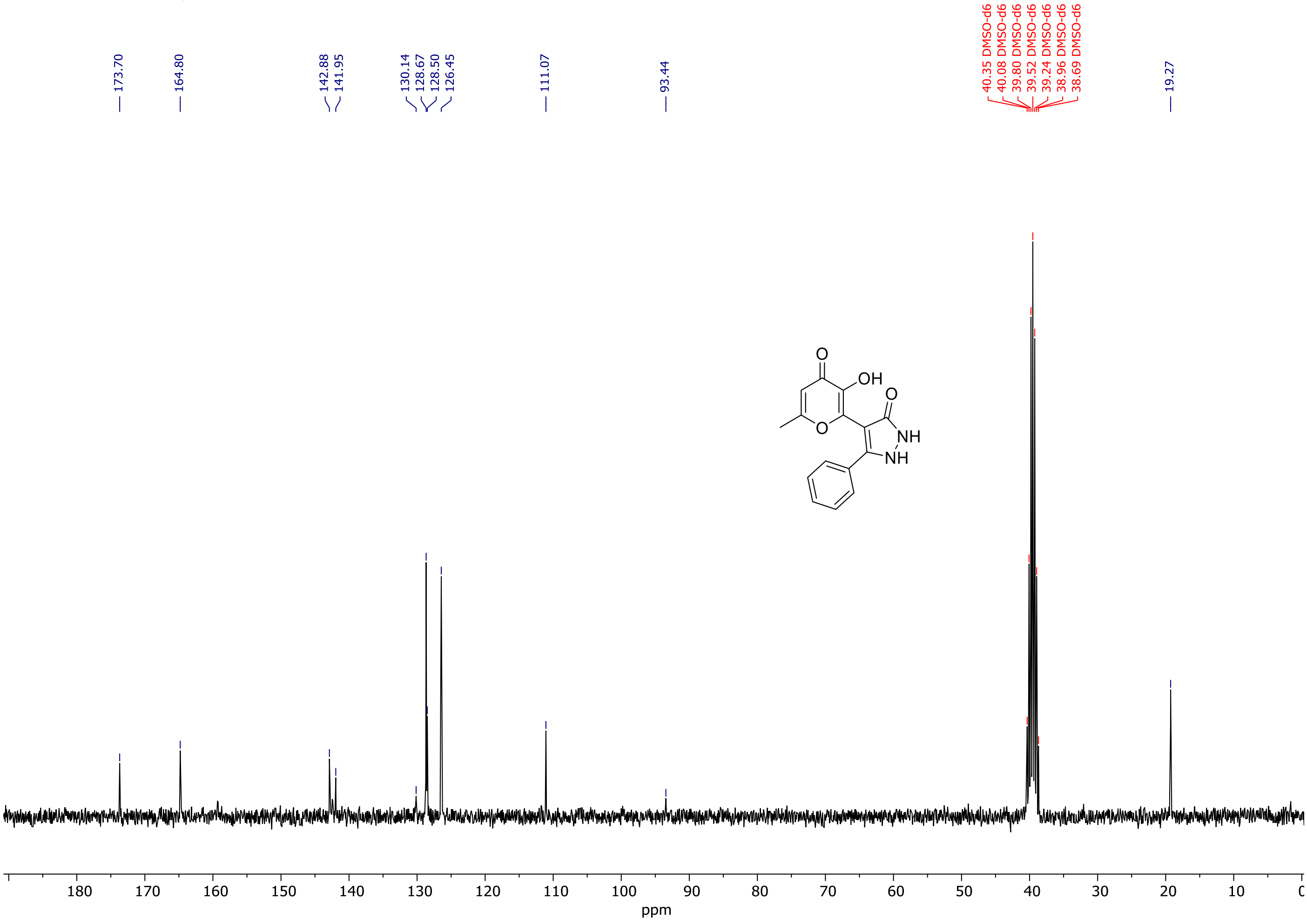

^{1}H NMR spectrum (300 MHz) of **10b** in DMSO-d_6

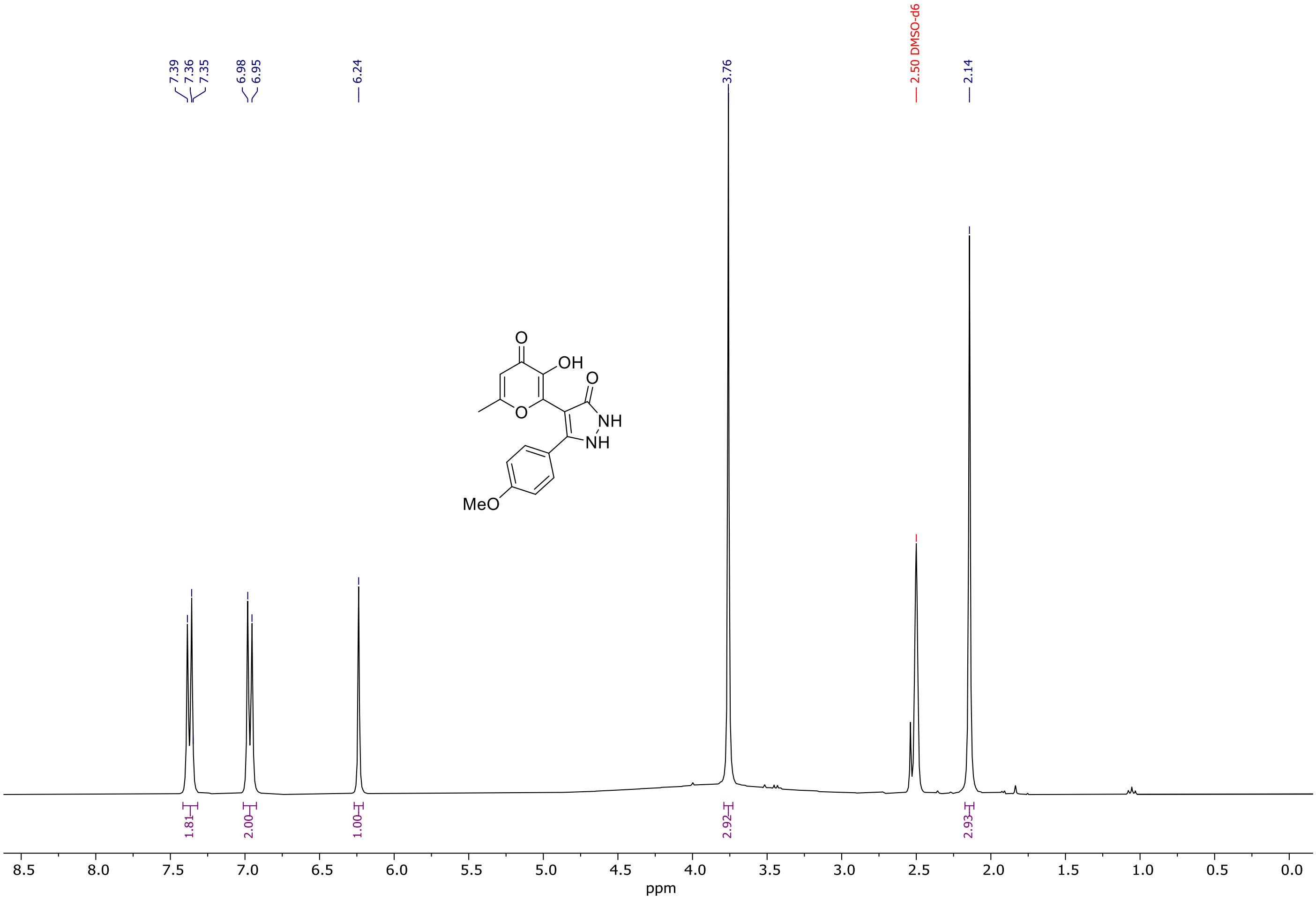

^{13}C {^{1}H} NMR spectrum (75 MHz) of **10b** in DMSO-d_6

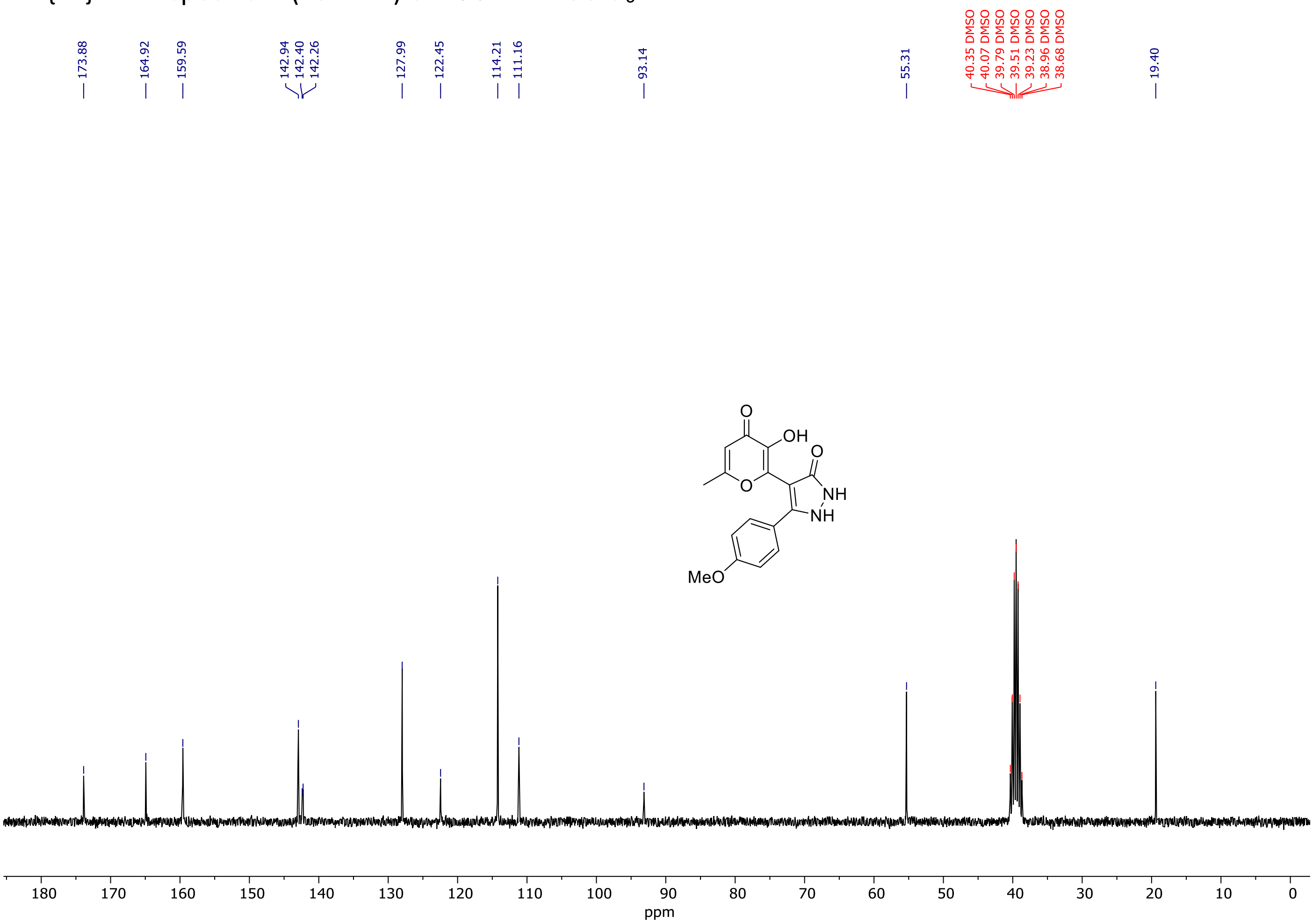

^{1}H NMR spectrum (300 MHz) of **10c** in DMSO-d_6

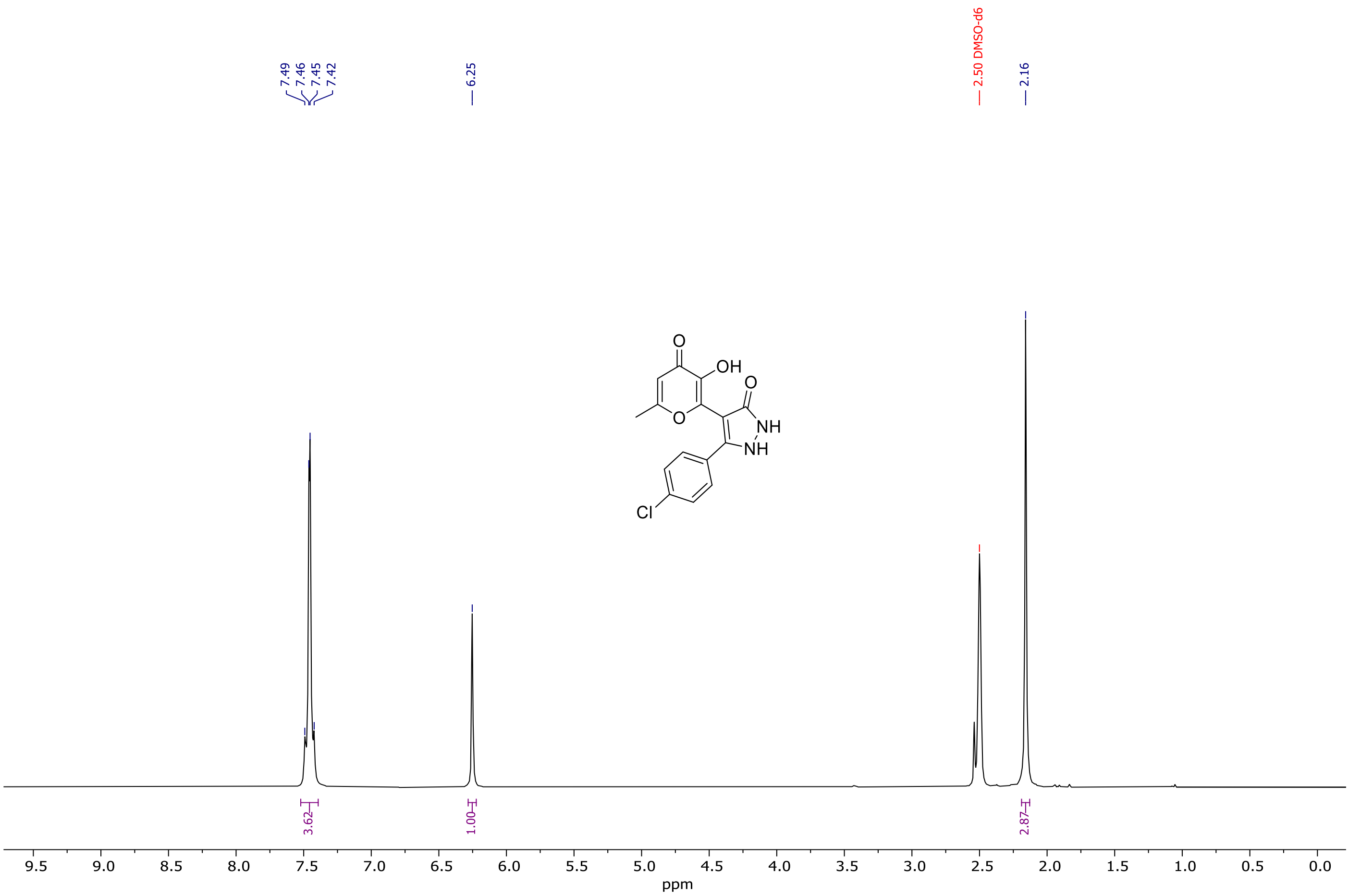

^{13}C {^{1}H} NMR spectrum (75 MHz) of **10c** in DMSO-d_6

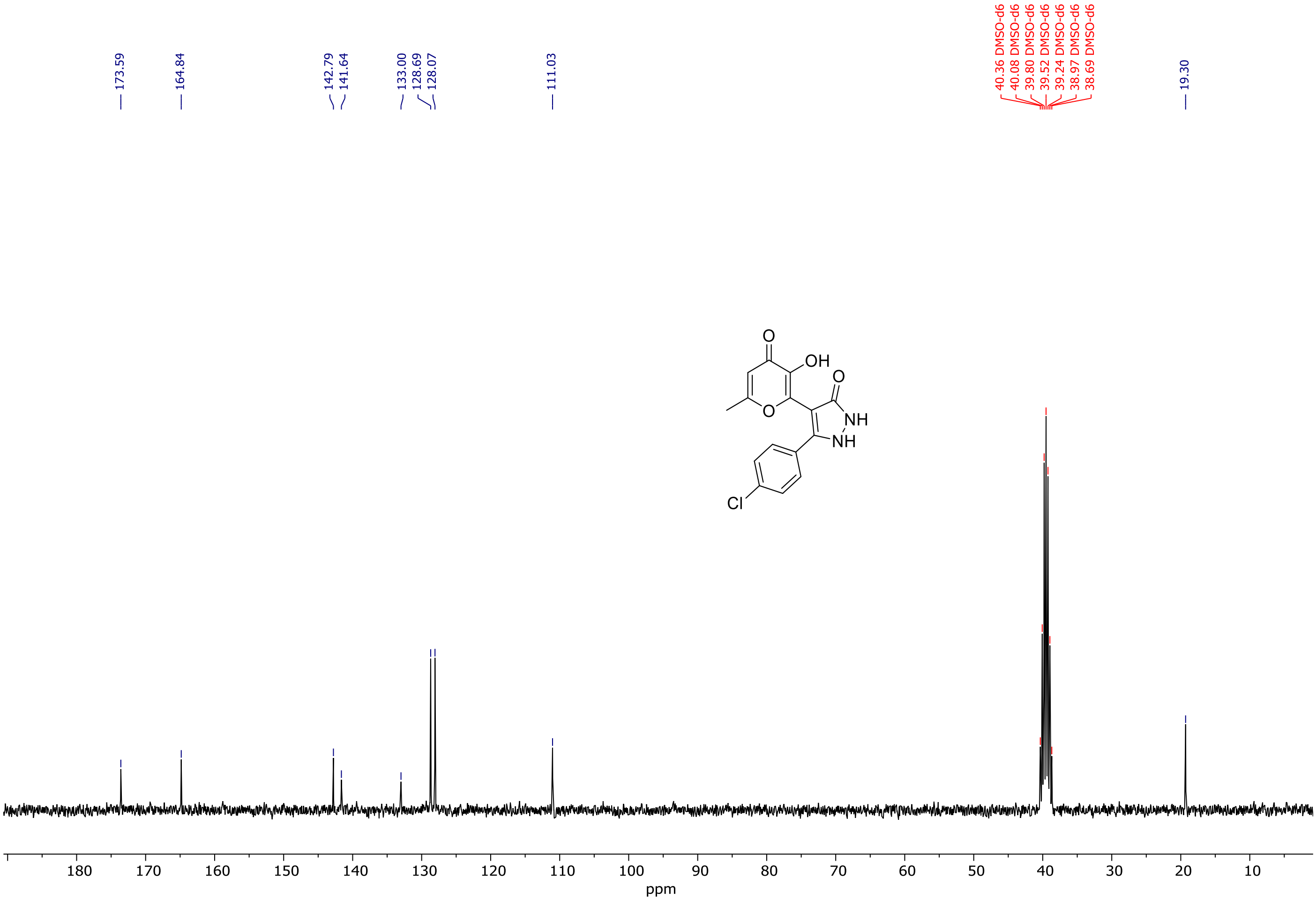

^{1}H NMR spectrum (300 MHz) of **10d** in DMSO-d_6

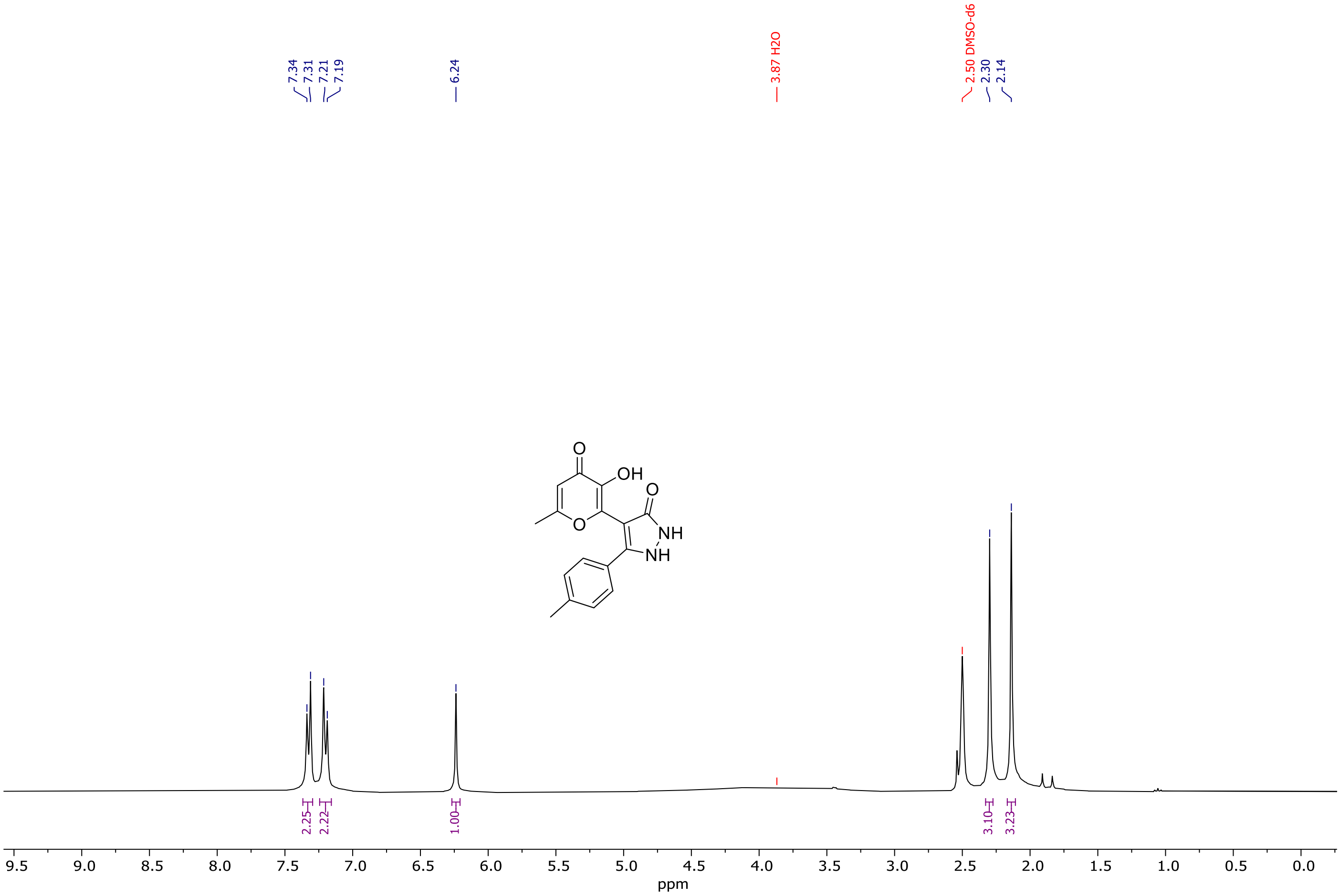

^{13}C {^{1}H} NMR spectrum (75 MHz) of **10d** in DMSO-d_6

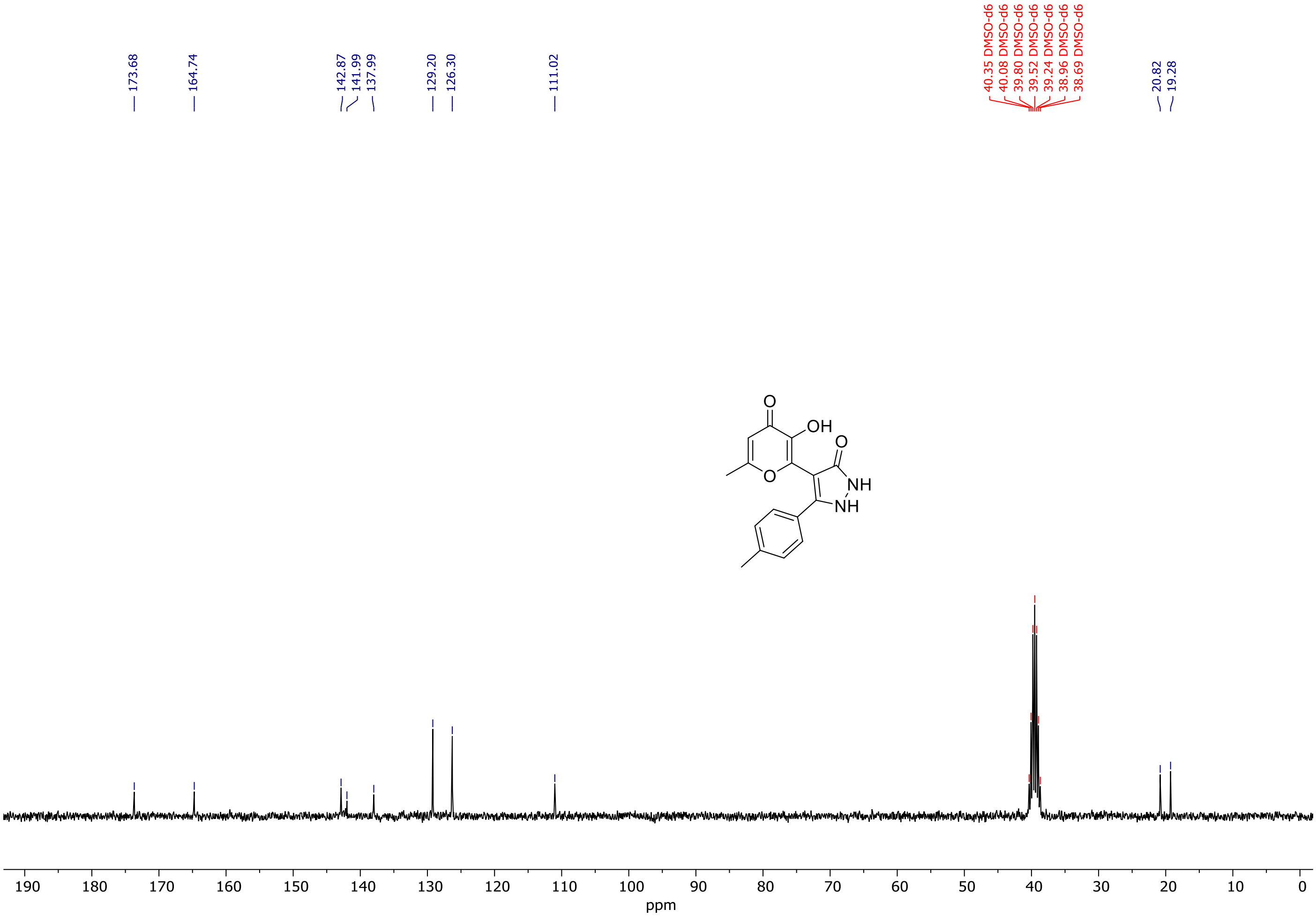

^{1}H NMR spectrum (300 MHz) of **10e** in DMSO-d_6

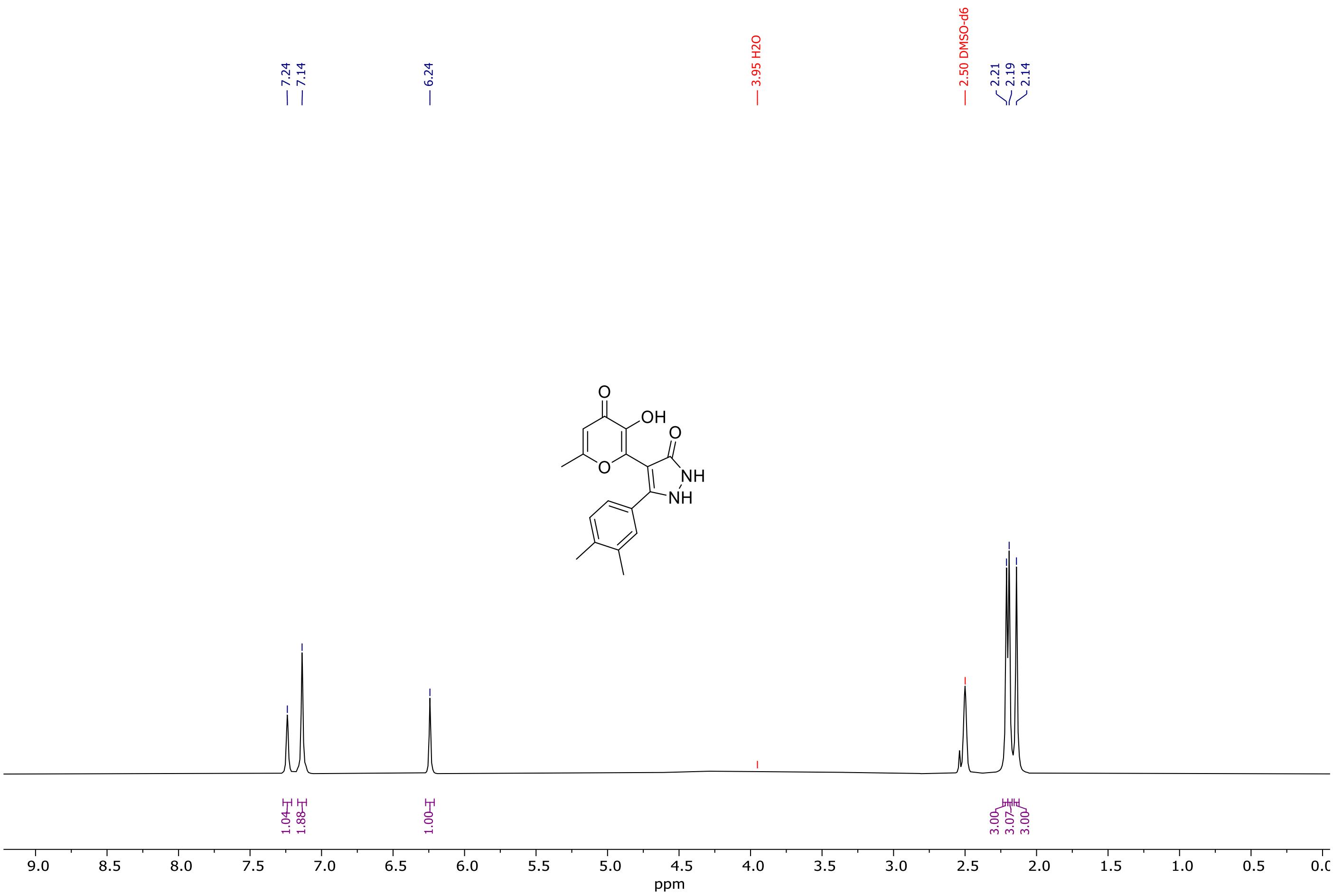

^{13}C {^{1}H} NMR spectrum (75 MHz) of **10e** in DMSO-d_6

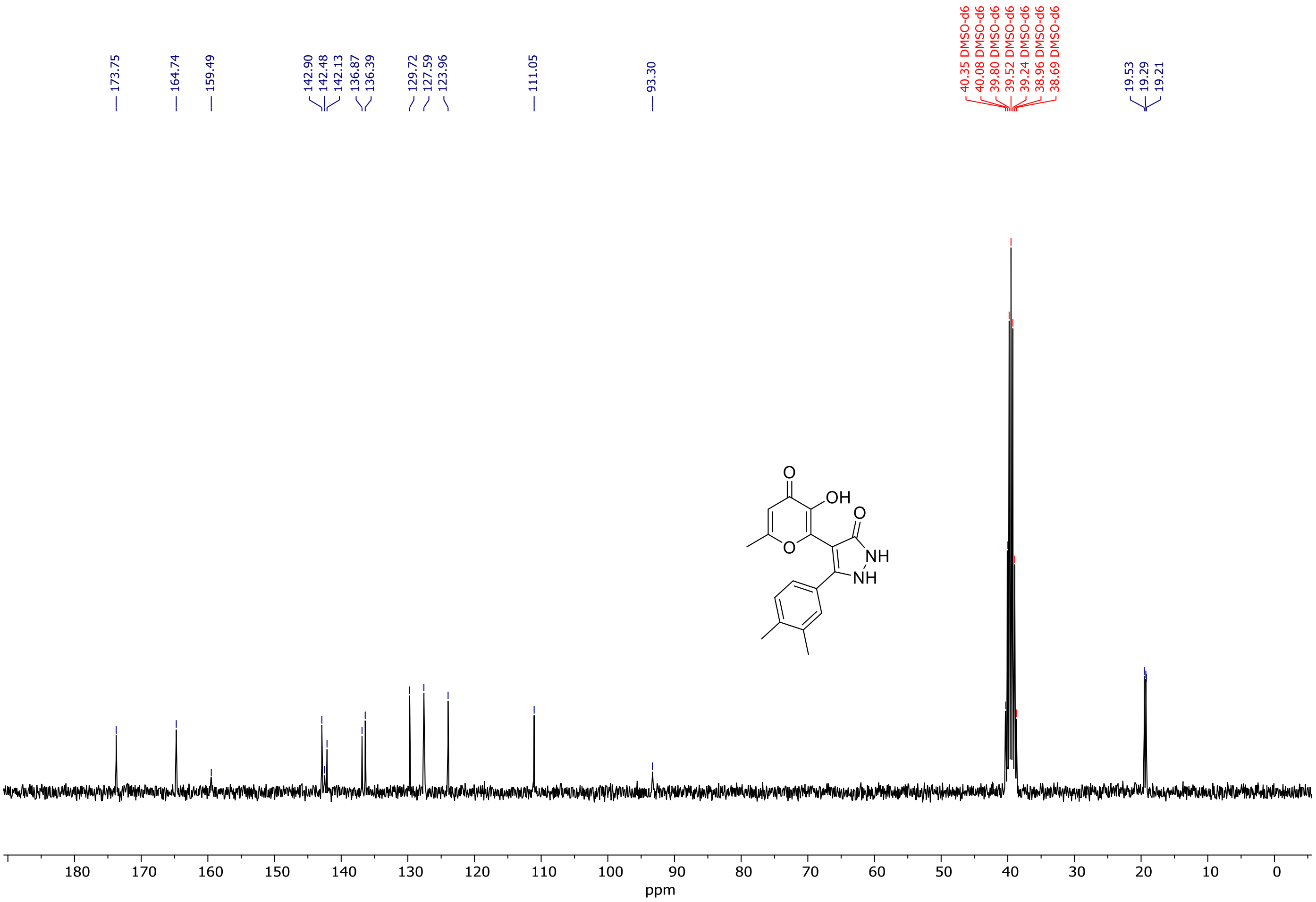

11. NMR ^{1}H and ^{13}C spectra for compounds 11 and 13

^{1}H NMR spectrum (300 MHz) of **11** in DMSO-d_6

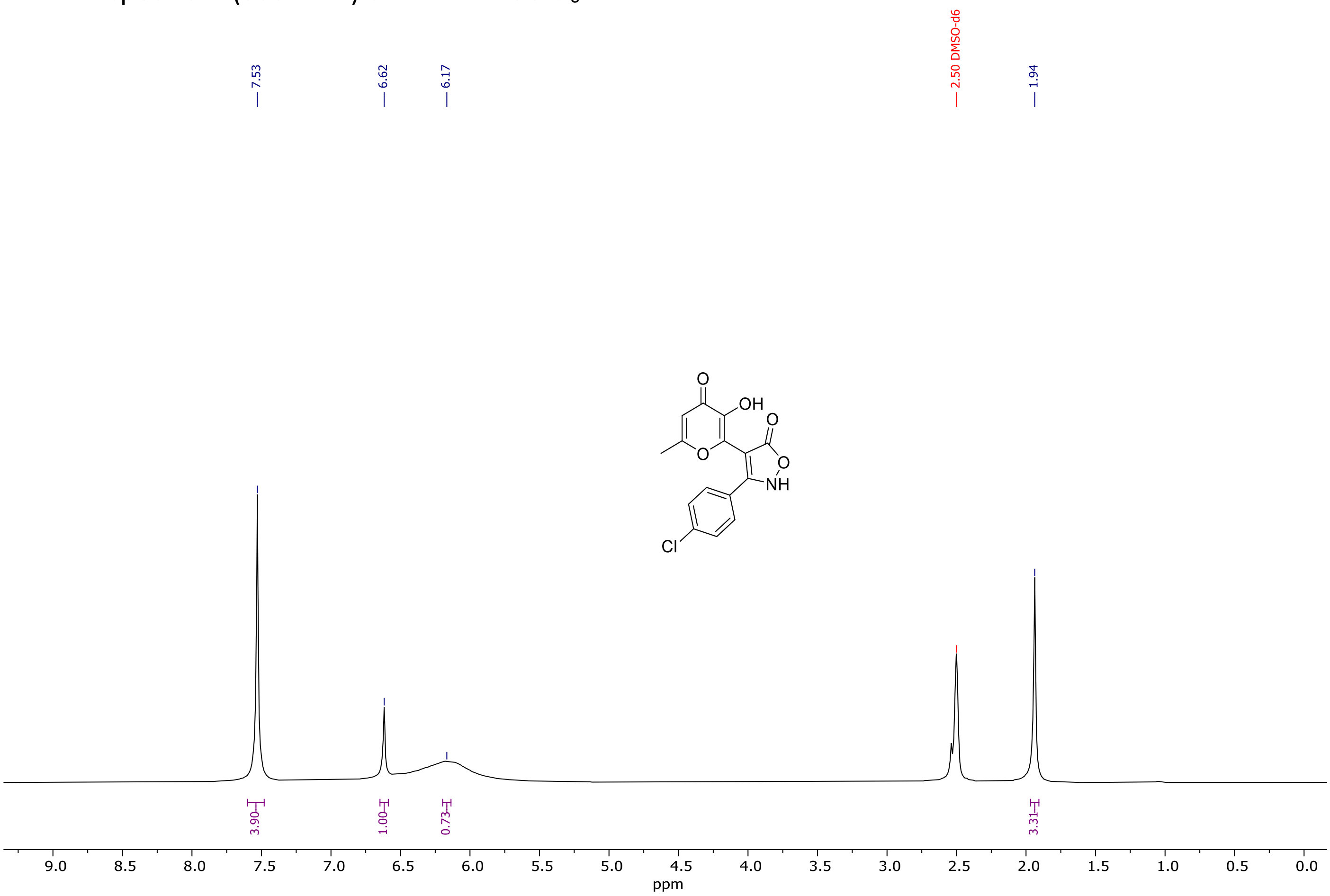

^{13}C {^{1}H} NMR spectrum (75 MHz) of **11** in DMSO-d_6

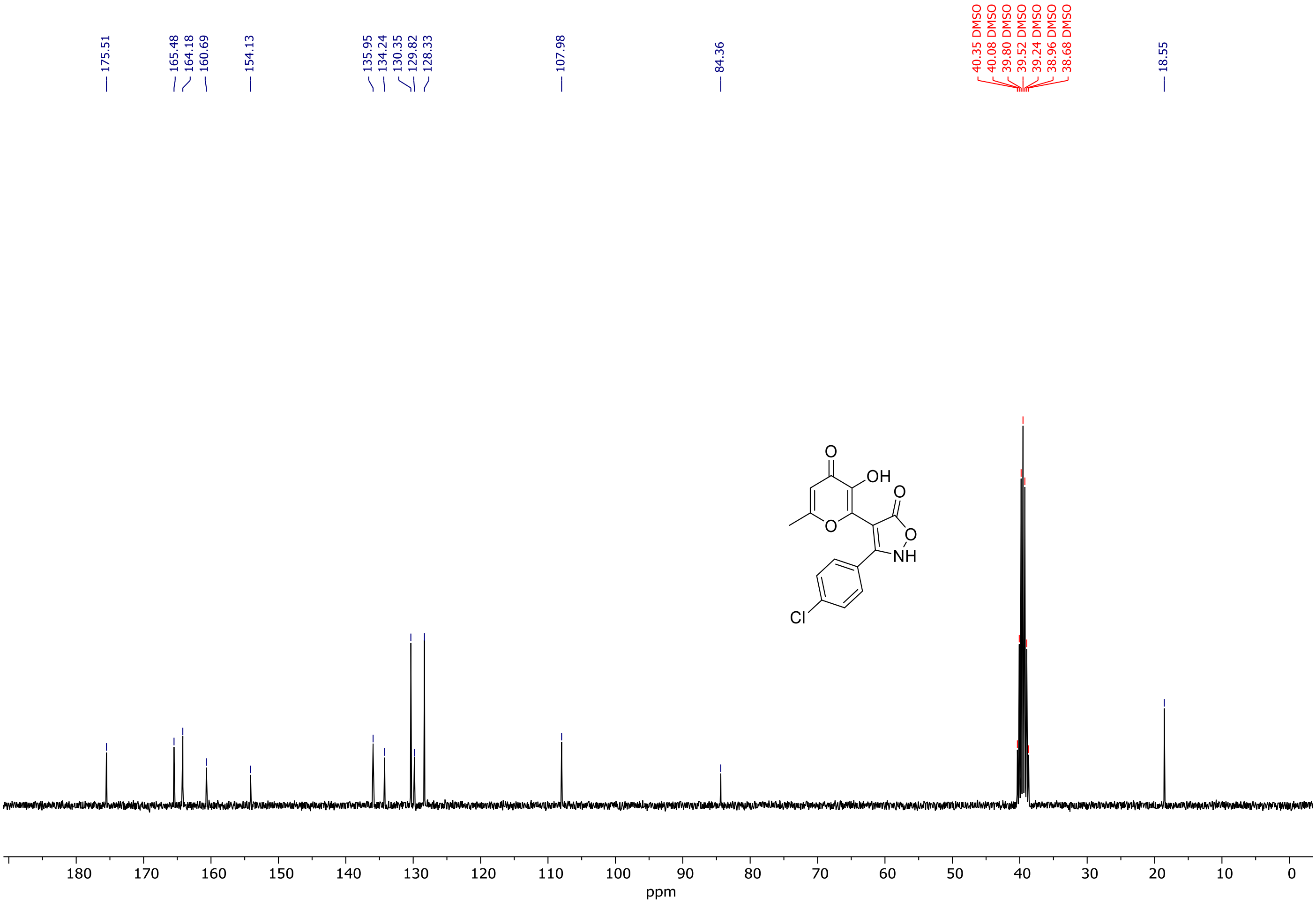

^{1}H NMR spectrum (300 MHz) of **13** in DMSO-d_6

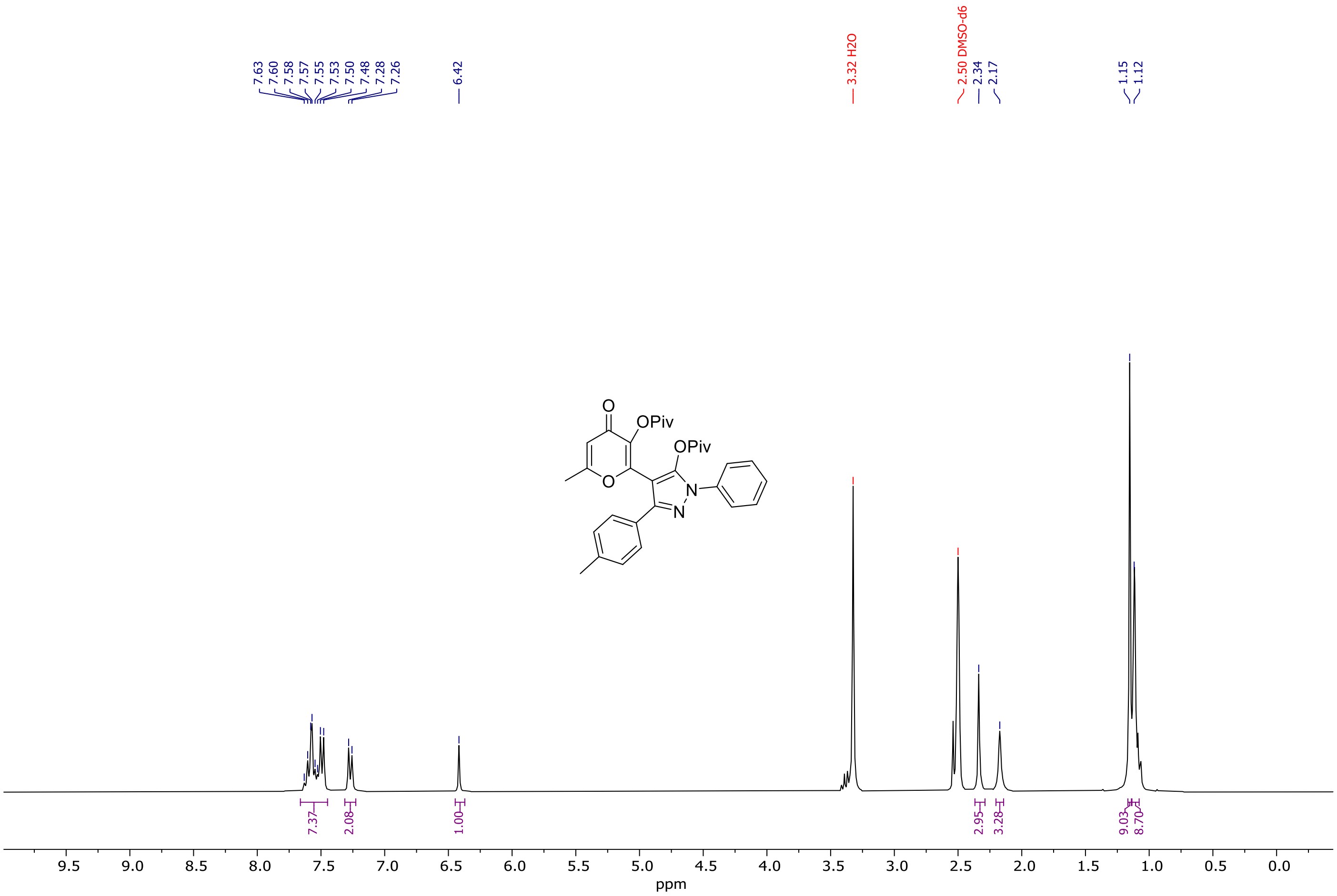

^{13}C {^{1}H} NMR spectrum (75 MHz) of **13** in DMSO-d_6

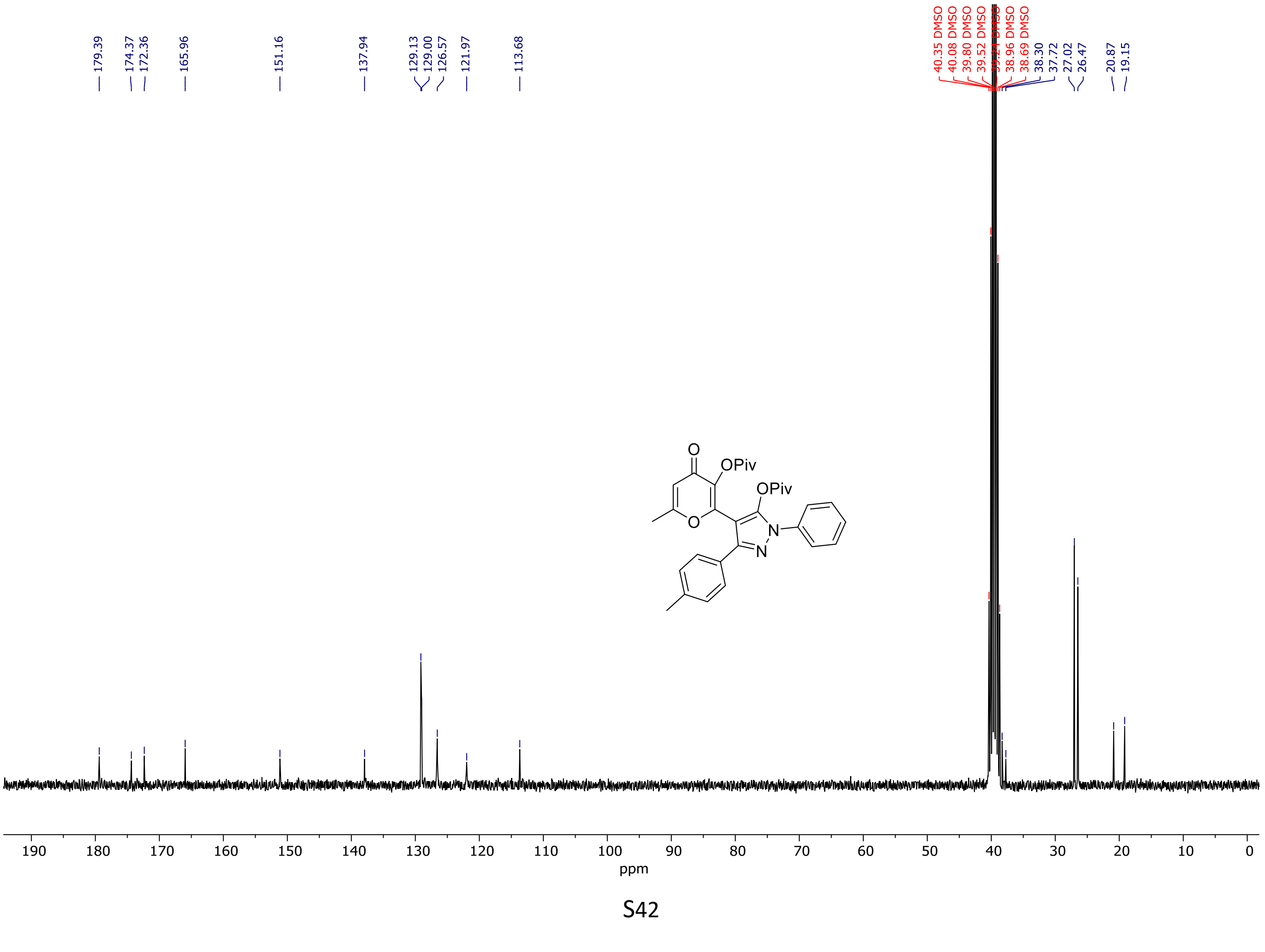

12. X-ray crystallographic data and refinement details

X-ray diffraction data were collected at 100K on a four-circle Rigaku Synergy S diffractometer equipped with a HyPix6000HE area-detector (kappa geometry, shutterless ω-scan technique), using graphite monochromatized Cu K_a-radiation. The intensity data were integrated and corrected for absorption and decay by the CrysAlisPro program[1]. The structure was solved by direct methods using SHELXT[2] and refined on F^2 using SHELXL-2018[3] in the OLEX2 program.[4] All non-hydrogen atoms were refined with individual anisotropic displacement parameters. The location of hydrogen atom at N1 (H1 atom) (for **4a**), amino and hydroxy hydrogen atoms (for **8o**), some of hydrogen atoms (H1, H2, H2A) (for 1**0c**) and (H4, H5, H6A, H6B) (for **11**) were found from the electron density-difference map; these atoms were refined with an individual isotropic displacement parameter. All other hydrogen atoms were placed in ideal calculated positions and refined as riding atoms with relative isotropic displacement parameters. For compound **13** all hydrogen atoms were placed in ideal calculated positions and refined as riding atoms with relative isotropic displacement parameters. A rotating group model was applied for methyl groups. The Mercury program suite[5] was used for molecular graphics.

1. CrysAlisPro. Version 1.171.41.106a. *Rigaku Oxford Diffraction*, **2021**.

2. Sheldrick, G. M. SHELXT - Integrated space-group and crystal-structure determination. *Acta Cryst.* **2015**, A71(1), 3-8. http://doi.org/10.1107/S2053273314026370

3. Sheldrick, G. M. Crystal structure refinement with SHELXL. *Acta Cryst.* **2015**, C71(1), 3-8. http://doi.org/10.1107/S2053229614024218

4. Dolomanov O.V.; Bourhis L.J.; Gildea R.J.; Howard J.A.K.; Puschmann H. OLEX2: a complete structure solution, refinement and analysis program. *J. Appl. Cryst.* **2009**, 42(2), 339-341. http://doi.org/10.1107/S0021889808042726

5. Macrae, C. F.; Sovago, I.; Cottrell, S. J.; Galek, P. T. A.; McCabe, P.; Pidcock, E.; Platings, M.; Shields, G. P.; Stevens, J. S.; Towler, M.; Wood, P. A. Mercury 4.0: from visualization to analysis, design and prediction. *J. Appl. Cryst.* **2020**, 53, 226-235. https://doi.org/10.1107/S1600576719014092

12.1 Crystallographic data for *(Z)-3-((benzylamino)(phenyl)methylene)-5-methyl-2H-furo[3,2-b]pyran-2,7(3H)-dione* (**4a**)

Table 1. Crystal data and structure refinement for **4a**

Identification code	2401913	
Empirical formula	$C_{22}H_{17}NO_4$	
Formula weight	359.36	
Temperature	100.02(10) K	
Wavelength	1.54184 Å	
Crystal system	Monoclinic	
Space group	$P2_1/c$	
Unit cell dimensions	a = 10.37770(10) Å	a= 90°.
	b = 10.39060(10) Å	b= 94.8020(10)°.
	c = 15.97540(10) Å	g = 90°.
Volume	1716.59(3) $Å^3$	
Z	4	
Density (calculated)	1.391 g/cm^3	
Absorption coefficient	0.787 mm^{-1}	
F(000)	752	
Crystal size	0.72 x 0.42 x 0.28 mm^3	
Theta range for data collection	4.275 to 79.992°.	
Index ranges	-13<=h<=13, -13<=k<=13, -16<=l<=20	
Reflections collected	22997	
Independent reflections	3727 [R(int) = 0.0313]	
Observed reflections	3588	
Completeness to theta = 67.684°	100.0 %	
Absorption correction	Gaussian	
Max. and min. transmission	1.000 and 0.280	
Refinement method	Full-matrix least-squares on F^2	
Data / restraints / parameters	3727 / 0 / 250	
Goodness-of-fit on F^2	1.068	
Final R indices [I>2sigma(I)]	R1 = 0.0352, wR2 = 0.0872	
R indices (all data)	R1 = 0.0363, wR2 = 0.0879	
Extinction coefficient	0.0025(3)	
Largest diff. peak and hole	0.292 and -0.180 e.$Å^{-3}$	

Table 2. Atomic coordinates (x 10^4) and equivalent isotropic displacement parameters ($Å^2$x 10^3) for **4a**. U(eq) is defined as one third of the trace of the orthogonalized U^{ij} tensor.

	x	y	z	U(eq)
O(1)	2414(1)	5159(1)	5214(1)	18(1)
O(2)	3705(1)	2674(1)	3443(1)	27(1)
O(3)	5023(1)	2967(1)	5151(1)	18(1)
O(4)	6173(1)	2988(1)	6423(1)	21(1)
N(1)	5003(1)	4583(1)	7580(1)	17(1)
C(1)	771(1)	6060(1)	4283(1)	26(1)
C(2)	1817(1)	5084(1)	4414(1)	20(1)
C(3)	2209(1)	4262(1)	3836(1)	21(1)
C(4)	3329(1)	3416(1)	3980(1)	19(1)
C(5)	3921(1)	3590(1)	4809(1)	17(1)
C(6)	3463(1)	4405(1)	5382(1)	16(1)
C(7)	4272(1)	4324(1)	6147(1)	16(1)
C(8)	5258(1)	3403(1)	5976(1)	17(1)
C(9)	4151(1)	4855(1)	6942(1)	16(1)
C(10)	3011(1)	5679(1)	7073(1)	16(1)
C(11)	2054(1)	5207(1)	7557(1)	20(1)
C(12)	946(1)	5931(1)	7639(1)	24(1)
C(13)	797(1)	7125(1)	7249(1)	24(1)
C(14)	1757(1)	7599(1)	6774(1)	22(1)
C(15)	2862(1)	6875(1)	6682(1)	19(1)
C(16)	4934(1)	5004(1)	8452(1)	18(1)
C(17)	6250(1)	5358(1)	8863(1)	17(1)
C(18)	6695(1)	4793(1)	9623(1)	20(1)
C(19)	7879(1)	5162(1)	10028(1)	24(1)
C(20)	8622(1)	6096(1)	9677(1)	25(1)
C(21)	8181(1)	6658(1)	8915(1)	23(1)
C(22)	7003(1)	6293(1)	8507(1)	18(1)

Table 3. Bond lengths [Å] and angles [°] for **4a**.

O(1)-C(2)	1.3744(13)
O(1)-C(6)	1.3495(13)
O(2)-C(4)	1.2400(14)
O(3)-C(5)	1.3860(13)
O(3)-C(8)	1.3962(13)
O(4)-C(8)	1.2177(13)
N(1)-H(1)	0.885(17)
N(1)-C(9)	1.3228(14)
N(1)-C(16)	1.4681(13)
C(1)-H(1A)	0.9800
C(1)-H(1B)	0.9800
C(1)-H(1C)	0.9800
C(1)-C(2)	1.4881(16)
C(2)-C(3)	1.3454(16)
C(3)-H(3)	0.9500
C(3)-C(4)	1.4610(16)
C(4)-C(5)	1.4242(15)
C(5)-C(6)	1.3605(15)
C(6)-C(7)	1.4269(15)
C(7)-C(8)	1.4439(15)
C(7)-C(9)	1.4000(15)
C(9)-C(10)	1.4892(15)
C(10)-C(11)	1.3969(15)
C(10)-C(15)	1.3934(16)
C(11)-H(11)	0.9500
C(11)-C(12)	1.3900(16)
C(12)-H(12)	0.9500
C(12)-C(13)	1.3908(18)
C(13)-H(13)	0.9500
C(13)-C(14)	1.3910(17)
C(14)-H(14)	0.9500
C(14)-C(15)	1.3902(16)
C(15)-H(15)	0.9500
C(16)-H(16A)	0.9900
C(16)-H(16B)	0.9900
C(16)-C(17)	1.5101(14)
C(17)-C(18)	1.3929(15)
C(17)-C(22)	1.3970(15)
C(18)-H(18)	0.9500
C(18)-C(19)	1.3935(17)
C(19)-H(19)	0.9500
C(19)-C(20)	1.3866(18)
C(20)-H(20)	0.9500
C(20)-C(21)	1.3931(17)
C(21)-H(21)	0.9500
C(21)-C(22)	1.3893(16)
C(22)-H(22)	0.9500
C(6)-O(1)-C(2)	116.43(9)
C(5)-O(3)-C(8)	107.07(8)
C(9)-N(1)-H(1)	115.4(10)
C(9)-N(1)-C(16)	125.79(10)
C(16)-N(1)-H(1)	118.4(10)
H(1A)-C(1)-H(1B)	109.5

H(1A)-C(1)-H(1C)	109.5
H(1B)-C(1)-H(1C)	109.5
C(2)-C(1)-H(1A)	109.5
C(2)-C(1)-H(1B)	109.5
C(2)-C(1)-H(1C)	109.5
O(1)-C(2)-C(1)	111.03(10)
C(3)-C(2)-O(1)	122.38(10)
C(3)-C(2)-C(1)	126.51(10)
C(2)-C(3)-H(3)	118.1
C(2)-C(3)-C(4)	123.78(10)
C(4)-C(3)-H(3)	118.1
O(2)-C(4)-C(3)	123.89(10)
O(2)-C(4)-C(5)	125.90(11)
C(5)-C(4)-C(3)	110.20(10)
O(3)-C(5)-C(4)	125.74(10)
C(6)-C(5)-O(3)	110.43(9)
C(6)-C(5)-C(4)	123.82(10)
O(1)-C(6)-C(5)	123.29(10)
O(1)-C(6)-C(7)	127.67(10)
C(5)-C(6)-C(7)	109.03(9)
C(6)-C(7)-C(8)	104.60(9)
C(9)-C(7)-C(6)	131.57(10)
C(9)-C(7)-C(8)	123.48(10)
O(3)-C(8)-C(7)	108.85(9)
O(4)-C(8)-O(3)	120.27(10)
O(4)-C(8)-C(7)	130.87(10)
N(1)-C(9)-C(7)	120.63(10)
N(1)-C(9)-C(10)	120.17(9)
C(7)-C(9)-C(10)	119.12(9)
C(11)-C(10)-C(9)	119.04(10)
C(15)-C(10)-C(9)	120.59(10)
C(15)-C(10)-C(11)	120.27(10)
C(10)-C(11)-H(11)	120.2
C(12)-C(11)-C(10)	119.58(11)
C(12)-C(11)-H(11)	120.2
C(11)-C(12)-H(12)	119.9
C(11)-C(12)-C(13)	120.16(11)
C(13)-C(12)-H(12)	119.9
C(12)-C(13)-H(13)	119.9
C(12)-C(13)-C(14)	120.16(11)
C(14)-C(13)-H(13)	119.9
C(13)-C(14)-H(14)	120.0
C(15)-C(14)-C(13)	120.04(11)
C(15)-C(14)-H(14)	120.0
C(10)-C(15)-H(15)	120.1
C(14)-C(15)-C(10)	119.78(10)
C(14)-C(15)-H(15)	120.1
N(1)-C(16)-H(16A)	109.3
N(1)-C(16)-H(16B)	109.3
N(1)-C(16)-C(17)	111.76(9)
H(16A)-C(16)-H(16B)	107.9
C(17)-C(16)-H(16A)	109.3
C(17)-C(16)-H(16B)	109.3
C(18)-C(17)-C(16)	119.85(10)
C(18)-C(17)-C(22)	119.50(10)
C(22)-C(17)-C(16)	120.58(10)

C(17)-C(18)-H(18)	119.9
C(17)-C(18)-C(19)	120.26(11)
C(19)-C(18)-H(18)	119.9
C(18)-C(19)-H(19)	119.9
C(20)-C(19)-C(18)	120.24(11)
C(20)-C(19)-H(19)	119.9
C(19)-C(20)-H(20)	120.2
C(19)-C(20)-C(21)	119.56(11)
C(21)-C(20)-H(20)	120.2
C(20)-C(21)-H(21)	119.7
C(22)-C(21)-C(20)	120.53(11)
C(22)-C(21)-H(21)	119.7
C(17)-C(22)-H(22)	120.0
C(21)-C(22)-C(17)	119.91(10)
C(21)-C(22)-H(22)	120.0

Table 4. Anisotropic displacement parameters ($Å^2$ x 10^3) for **4a**. The anisotropic displacement factor exponent takes the form: $-2p^2[h^2 a^{*2}U^{11} + ... + 2 h k a^* b^* U^{12}]$

	U^{11}	U^{22}	U^{33}	U^{23}	U^{13}	U^{12}
O(1)	17(1)	20(1)	17(1)	-1(1)	-1(1)	3(1)
O(2)	42(1)	22(1)	17(1)	-3(1)	1(1)	8(1)
O(3)	20(1)	17(1)	17(1)	0(1)	1(1)	4(1)
O(4)	19(1)	20(1)	22(1)	2(1)	-1(1)	4(1)
N(1)	18(1)	16(1)	17(1)	-1(1)	0(1)	1(1)
C(1)	23(1)	28(1)	25(1)	1(1)	-3(1)	7(1)
C(2)	18(1)	21(1)	18(1)	2(1)	-2(1)	0(1)
C(3)	24(1)	21(1)	17(1)	1(1)	-3(1)	-1(1)
C(4)	26(1)	16(1)	17(1)	1(1)	2(1)	0(1)
C(5)	18(1)	15(1)	18(1)	2(1)	2(1)	1(1)
C(6)	16(1)	14(1)	17(1)	1(1)	2(1)	-1(1)
C(7)	16(1)	15(1)	18(1)	1(1)	1(1)	0(1)
C(8)	19(1)	15(1)	17(1)	1(1)	2(1)	-2(1)
C(9)	16(1)	13(1)	18(1)	1(1)	1(1)	-3(1)
C(10)	16(1)	18(1)	15(1)	-4(1)	-1(1)	-1(1)
C(11)	20(1)	22(1)	18(1)	1(1)	0(1)	-2(1)
C(12)	18(1)	32(1)	21(1)	-1(1)	3(1)	-1(1)
C(13)	18(1)	29(1)	24(1)	-5(1)	0(1)	6(1)
C(14)	24(1)	20(1)	23(1)	-2(1)	-1(1)	3(1)
C(15)	20(1)	19(1)	18(1)	-1(1)	1(1)	-1(1)
C(16)	19(1)	19(1)	16(1)	-2(1)	1(1)	-2(1)
C(17)	18(1)	16(1)	16(1)	-3(1)	1(1)	2(1)
C(18)	22(1)	20(1)	18(1)	1(1)	2(1)	1(1)
C(19)	24(1)	30(1)	18(1)	1(1)	-3(1)	5(1)
C(20)	17(1)	32(1)	26(1)	-5(1)	-3(1)	0(1)
C(21)	20(1)	23(1)	25(1)	-2(1)	4(1)	-3(1)
C(22)	21(1)	18(1)	17(1)	0(1)	2(1)	1(1)

Table 5. Hydrogen coordinates (x 10^4) and isotropic displacement parameters ($Å^2$ x 10^3) for **4a**.

	x	y	z	U(eq)
H(1)	5613(15)	4027(16)	7470(9)	26(4)
H(1A)	305	6122	4791	39
H(1B)	169	5803	3807	39
H(1C)	1149	6898	4166	39
H(3)	1729	4231	3303	25
H(11)	2161	4395	7828	24
H(12)	289	5611	7963	28
H(13)	39	7618	7307	28
H(14)	1656	8418	6513	27
H(15)	3514	7194	6353	23
H(16A)	4562	4304	8777	22
H(16B)	4353	5758	8463	22
H(18)	6189	4154	9867	24
H(19)	8179	4772	10546	29
H(20)	9427	6351	9955	30
H(21)	8689	7296	8672	27
H(22)	6709	6679	7986	22

Table 6. Torsion angles [°] for **4a**.

O(1)-C(2)-C(3)-C(4)	-2.83(18)
O(1)-C(6)-C(7)-C(8)	-178.54(10)
O(1)-C(6)-C(7)-C(9)	8.19(19)
O(2)-C(4)-C(5)-O(3)	0.60(19)
O(2)-C(4)-C(5)-C(6)	-179.08(11)
O(3)-C(5)-C(6)-O(1)	178.49(9)
O(3)-C(5)-C(6)-C(7)	-0.73(12)
N(1)-C(9)-C(10)-C(11)	-67.86(14)
N(1)-C(9)-C(10)-C(15)	115.65(12)
N(1)-C(16)-C(17)-C(18)	-126.48(11)
N(1)-C(16)-C(17)-C(22)	56.51(13)
C(1)-C(2)-C(3)-C(4)	173.49(11)
C(2)-O(1)-C(6)-C(5)	-0.83(15)
C(2)-O(1)-C(6)-C(7)	178.24(10)
C(2)-C(3)-C(4)-O(2)	-178.71(12)
C(2)-C(3)-C(4)-C(5)	0.24(16)
C(3)-C(4)-C(5)-O(3)	-178.33(10)
C(3)-C(4)-C(5)-C(6)	1.99(15)
C(4)-C(5)-C(6)-O(1)	-1.79(17)
C(4)-C(5)-C(6)-C(7)	178.98(10)
C(5)-O(3)-C(8)-O(4)	179.14(10)
C(5)-O(3)-C(8)-C(7)	-0.09(11)
C(5)-C(6)-C(7)-C(8)	0.64(12)
C(5)-C(6)-C(7)-C(9)	-172.63(11)
C(6)-O(1)-C(2)-C(1)	-173.76(9)
C(6)-O(1)-C(2)-C(3)	3.07(16)
C(6)-C(7)-C(8)-O(3)	-0.33(11)
C(6)-C(7)-C(8)-O(4)	-179.45(11)
C(6)-C(7)-C(9)-N(1)	176.10(11)
C(6)-C(7)-C(9)-C(10)	-0.78(17)
C(7)-C(9)-C(10)-C(11)	109.04(12)
C(7)-C(9)-C(10)-C(15)	-67.45(14)
C(8)-O(3)-C(5)-C(4)	-179.20(10)
C(8)-O(3)-C(5)-C(6)	0.51(12)
C(8)-C(7)-C(9)-N(1)	3.92(16)
C(8)-C(7)-C(9)-C(10)	-172.96(10)
C(9)-N(1)-C(16)-C(17)	-141.25(11)
C(9)-C(7)-C(8)-O(3)	173.64(9)
C(9)-C(7)-C(8)-O(4)	-5.48(19)
C(9)-C(10)-C(11)-C(12)	-175.88(10)
C(9)-C(10)-C(15)-C(14)	176.44(10)
C(10)-C(11)-C(12)-C(13)	-0.63(17)
C(11)-C(10)-C(15)-C(14)	0.00(16)
C(11)-C(12)-C(13)-C(14)	0.02(17)
C(12)-C(13)-C(14)-C(15)	0.61(17)
C(13)-C(14)-C(15)-C(10)	-0.61(17)
C(15)-C(10)-C(11)-C(12)	0.62(16)
C(16)-N(1)-C(9)-C(7)	-175.53(10)
C(16)-N(1)-C(9)-C(10)	1.32(16)
C(16)-C(17)-C(18)-C(19)	-176.76(10)
C(16)-C(17)-C(22)-C(21)	176.57(10)
C(17)-C(18)-C(19)-C(20)	0.14(18)
C(18)-C(17)-C(22)-C(21)	-0.46(16)
C(18)-C(19)-C(20)-C(21)	-0.40(18)

C(19)-C(20)-C(21)-C(22)	0.23(18)
C(20)-C(21)-C(22)-C(17)	0.20(17)
C(22)-C(17)-C(18)-C(19)	0.29(16)

Table 7. Hydrogen bonds for **4a** [Å and °].

D-H...A	d(D-H)	d(H...A)	d(D...A)	<(DHA)
N(1)-H(1)...O(4)	0.885(17)	2.113(16)	2.8309(12)	137.7(13)

12.2 Crystallographic data for *4-(3-hydroxy-6-methyl-4-oxo-4H-pyran-2-yl)-2-phenyl-5-(p-tolyl)-1,2-dihydro-3H-pyrazol-3-one* (**8o**)

Table 8. Crystal data and structure refinement for **8o**.

Identification code	2402120	
Empirical formula	$C_{22}H_{18}N_2O_4$	
Formula weight	374.38	
Temperature	100.00(10) K	
Wavelength	1.54184 Å	
Crystal system	Triclinic	
Space group	P-1	
Unit cell dimensions	a = 9.3891(2) Å	a= 72.702(2)°.
	b = 9.7478(2) Å	b= 69.595(2)°.
	c = 11.7537(3) Å	g = 61.845(3)°.
Volume	877.38(4) Å^3	
Z	2	
Density (calculated)	1.417 Mg/m^3	
Absorption coefficient	0.810 mm^{-1}	
F(000)	392	
Crystal size	0.69 x 0.24 x 0.19 mm^3	
Theta range for data collection	4.066 to 79.717°.	
Index ranges	-10<=h<=11, -11<=k<=12, -14<=l<=14	
Reflections collected	23341	
Independent reflections	3793 [R(int) = 0.0345]	
Completeness to theta = 67.684°	100.0 %	
Absorption correction	Gaussian	
Max. and min. transmission	1.000 and 0.254	
Refinement method	Full-matrix least-squares on F^2	
Data / restraints / parameters	3793 / 0 / 263	
Goodness-of-fit on F^2	1.045	
Final R indices [I>2sigma(I)]	R1 = 0.0404, wR2 = 0.1084	
R indices (all data)	R1 = 0.0412, wR2 = 0.1091	
Largest diff. peak and hole	0.260 and -0.295 e.Å^{-3}	

Table 9. Atomic coordinates (x 10^4) and equivalent isotropic displacement parameters ($Å^2$x 10^3) for **8o**. U(eq) is defined as one third of the trace of the orthogonalized U^{ij} tensor.

	x	y	z	U(eq)
O(1)	5776(1)	7196(1)	6057(1)	17(1)
O(2)	9442(1)	5841(1)	2637(1)	21(1)
O(3)	9384(1)	7722(1)	3817(1)	19(1)
O(4)	8016(1)	10208(1)	4992(1)	21(1)
N(1)	8872(1)	3623(1)	3490(1)	17(1)
N(2)	7977(1)	3105(1)	4598(1)	16(1)
C(1)	8780(1)	5059(1)	3533(1)	16(1)
C(2)	7802(1)	5408(1)	4738(1)	15(1)
C(3)	7371(1)	4142(1)	5363(1)	15(1)
C(4)	9495(1)	2841(1)	2451(1)	16(1)
C(5)	9160(1)	3732(1)	1332(1)	19(1)
C(6)	9714(2)	2973(2)	328(1)	22(1)
C(7)	10577(2)	1341(2)	457(1)	24(1)
C(8)	10908(2)	468(1)	1576(1)	23(1)
C(9)	10381(1)	1213(1)	2586(1)	19(1)
C(10)	7250(1)	6880(1)	5158(1)	15(1)
C(11)	7994(1)	7914(1)	4739(1)	16(1)
C(12)	7325(1)	9277(1)	5338(1)	17(1)
C(13)	5865(1)	9442(1)	6336(1)	18(1)
C(14)	5123(1)	8445(1)	6638(1)	17(1)
C(15)	3529(1)	8574(1)	7570(1)	21(1)
C(16)	6467(1)	3792(1)	6639(1)	16(1)
C(17)	5324(1)	3142(1)	6886(1)	17(1)
C(18)	4506(1)	2761(1)	8089(1)	19(1)
C(19)	4800(1)	3011(1)	9077(1)	19(1)
C(20)	5948(1)	3660(1)	8821(1)	18(1)
C(21)	6771(1)	4050(1)	7622(1)	17(1)
C(22)	3909(2)	2591(2)	10379(1)	26(1)

Table 10. Bond lengths [Å] and angles [°] for **8o**.

O(1)-C(10)	1.3778(13)
O(1)-C(14)	1.3509(13)
O(2)-C(1)	1.2611(14)
O(3)-H(3)	0.91(2)
O(3)-C(11)	1.3480(13)
O(4)-C(12)	1.2502(14)
N(1)-N(2)	1.3790(13)
N(1)-C(1)	1.3768(14)
N(1)-C(4)	1.4314(14)
N(2)-H(2)	0.939(19)
N(2)-C(3)	1.3393(14)
C(1)-C(2)	1.4317(15)
C(2)-C(3)	1.3974(15)
C(2)-C(10)	1.4528(14)
C(3)-C(16)	1.4722(15)
C(4)-C(5)	1.3915(16)
C(4)-C(9)	1.3898(16)
C(5)-H(5)	0.9500
C(5)-C(6)	1.3918(16)
C(6)-H(6)	0.9500
C(6)-C(7)	1.3922(18)
C(7)-H(7)	0.9500
C(7)-C(8)	1.3861(19)
C(8)-H(8)	0.9500
C(8)-C(9)	1.3945(16)
C(9)-H(9)	0.9500
C(10)-C(11)	1.3692(15)
C(11)-C(12)	1.4544(15)
C(12)-C(13)	1.4370(16)
C(13)-H(13)	0.9500
C(13)-C(14)	1.3481(16)
C(14)-C(15)	1.4888(15)
C(15)-H(15A)	0.9800
C(15)-H(15B)	0.9800
C(15)-H(15C)	0.9800
C(16)-C(17)	1.3983(15)
C(16)-C(21)	1.3967(15)
C(17)-H(17)	0.9500
C(17)-C(18)	1.3876(16)
C(18)-H(18)	0.9500
C(18)-C(19)	1.3931(16)
C(19)-C(20)	1.3985(16)
C(19)-C(22)	1.5063(16)
C(20)-H(20)	0.9500
C(20)-C(21)	1.3885(15)
C(21)-H(21)	0.9500
C(22)-H(22A)	0.9800
C(22)-H(22B)	0.9800
C(22)-H(22C)	0.9800
C(14)-O(1)-C(10)	120.69(8)
C(11)-O(3)-H(3)	109.6(13)
N(2)-N(1)-C(4)	121.22(9)
C(1)-N(1)-N(2)	109.14(9)

C(1)-N(1)-C(4)	128.76(9)
N(1)-N(2)-H(2)	119.8(11)
C(3)-N(2)-N(1)	109.22(9)
C(3)-N(2)-H(2)	130.1(11)
O(2)-C(1)-N(1)	123.40(10)
O(2)-C(1)-C(2)	130.61(10)
N(1)-C(1)-C(2)	105.98(9)
C(1)-C(2)-C(10)	125.20(10)
C(3)-C(2)-C(1)	106.87(9)
C(3)-C(2)-C(10)	127.68(10)
N(2)-C(3)-C(2)	108.72(10)
N(2)-C(3)-C(16)	119.45(9)
C(2)-C(3)-C(16)	131.80(10)
C(5)-C(4)-N(1)	118.89(10)
C(9)-C(4)-N(1)	119.65(10)
C(9)-C(4)-C(5)	121.44(10)
C(4)-C(5)-H(5)	120.4
C(4)-C(5)-C(6)	119.21(11)
C(6)-C(5)-H(5)	120.4
C(5)-C(6)-H(6)	120.1
C(5)-C(6)-C(7)	119.88(11)
C(7)-C(6)-H(6)	120.1
C(6)-C(7)-H(7)	119.8
C(8)-C(7)-C(6)	120.32(11)
C(8)-C(7)-H(7)	119.8
C(7)-C(8)-H(8)	119.8
C(7)-C(8)-C(9)	120.46(11)
C(9)-C(8)-H(8)	119.8
C(4)-C(9)-C(8)	118.68(11)
C(4)-C(9)-H(9)	120.7
C(8)-C(9)-H(9)	120.7
O(1)-C(10)-C(2)	111.53(9)
C(11)-C(10)-O(1)	120.85(9)
C(11)-C(10)-C(2)	127.59(10)
O(3)-C(11)-C(10)	123.97(10)
O(3)-C(11)-C(12)	116.20(9)
C(10)-C(11)-C(12)	119.77(10)
O(4)-C(12)-C(11)	120.71(10)
O(4)-C(12)-C(13)	123.84(10)
C(13)-C(12)-C(11)	115.44(9)
C(12)-C(13)-H(13)	119.2
C(14)-C(13)-C(12)	121.55(10)
C(14)-C(13)-H(13)	119.2
O(1)-C(14)-C(15)	112.00(9)
C(13)-C(14)-O(1)	121.24(10)
C(13)-C(14)-C(15)	126.72(10)
C(14)-C(15)-H(15A)	109.5
C(14)-C(15)-H(15B)	109.5
C(14)-C(15)-H(15C)	109.5
H(15A)-C(15)-H(15B)	109.5
H(15A)-C(15)-H(15C)	109.5
H(15B)-C(15)-H(15C)	109.5
C(17)-C(16)-C(3)	120.01(10)
C(21)-C(16)-C(3)	121.08(10)
C(21)-C(16)-C(17)	118.87(10)
C(16)-C(17)-H(17)	119.8

C(18)-C(17)-C(16)	120.41(10)
C(18)-C(17)-H(17)	119.8
C(17)-C(18)-H(18)	119.4
C(17)-C(18)-C(19)	121.24(10)
C(19)-C(18)-H(18)	119.4
C(18)-C(19)-C(20)	117.97(10)
C(18)-C(19)-C(22)	120.80(10)
C(20)-C(19)-C(22)	121.23(11)
C(19)-C(20)-H(20)	119.3
C(21)-C(20)-C(19)	121.37(10)
C(21)-C(20)-H(20)	119.3
C(16)-C(21)-H(21)	119.9
C(20)-C(21)-C(16)	120.13(10)
C(20)-C(21)-H(21)	119.9
C(19)-C(22)-H(22A)	109.5
C(19)-C(22)-H(22B)	109.5
C(19)-C(22)-H(22C)	109.5
H(22A)-C(22)-H(22B)	109.5
H(22A)-C(22)-H(22C)	109.5
H(22B)-C(22)-H(22C)	109.5

Table 11. Anisotropic displacement parameters (Å^2 x 10^3) for **8o**. The anisotropic displacement factor exponent takes the form: $-2p^2[h^2 a^{*2}U^{11} + ... + 2 h k a^* b^* U^{12}]$

	U^{11}	U^{22}	U^{33}	U^{23}	U^{13}	U^{12}
O(1)	16(1)	16(1)	20(1)	-6(1)	-1(1)	-9(1)
O(2)	28(1)	22(1)	18(1)	-4(1)	0(1)	-18(1)
O(3)	18(1)	20(1)	22(1)	-7(1)	0(1)	-12(1)
O(4)	27(1)	18(1)	24(1)	-4(1)	-4(1)	-15(1)
N(1)	21(1)	17(1)	16(1)	-3(1)	0(1)	-12(1)
N(2)	19(1)	16(1)	16(1)	-3(1)	-1(1)	-11(1)
C(1)	18(1)	16(1)	18(1)	-4(1)	-4(1)	-9(1)
C(2)	16(1)	15(1)	18(1)	-3(1)	-3(1)	-8(1)
C(3)	15(1)	15(1)	18(1)	-4(1)	-4(1)	-7(1)
C(4)	15(1)	19(1)	19(1)	-6(1)	-1(1)	-10(1)
C(5)	19(1)	19(1)	21(1)	-3(1)	-3(1)	-10(1)
C(6)	25(1)	30(1)	18(1)	-4(1)	-3(1)	-18(1)
C(7)	25(1)	31(1)	24(1)	-14(1)	4(1)	-18(1)
C(8)	20(1)	18(1)	31(1)	-10(1)	1(1)	-9(1)
C(9)	17(1)	19(1)	22(1)	-4(1)	-3(1)	-10(1)
C(10)	14(1)	15(1)	16(1)	-3(1)	-3(1)	-6(1)
C(11)	14(1)	15(1)	18(1)	-2(1)	-4(1)	-7(1)
C(12)	20(1)	15(1)	19(1)	-1(1)	-7(1)	-9(1)
C(13)	21(1)	15(1)	19(1)	-5(1)	-4(1)	-8(1)
C(14)	17(1)	16(1)	18(1)	-4(1)	-5(1)	-6(1)
C(15)	18(1)	24(1)	23(1)	-8(1)	-1(1)	-10(1)
C(16)	16(1)	13(1)	18(1)	-3(1)	-2(1)	-6(1)
C(17)	17(1)	16(1)	20(1)	-4(1)	-4(1)	-8(1)
C(18)	16(1)	19(1)	22(1)	-3(1)	-3(1)	-10(1)
C(19)	16(1)	18(1)	19(1)	-3(1)	-2(1)	-6(1)
C(20)	17(1)	18(1)	19(1)	-5(1)	-4(1)	-7(1)
C(21)	15(1)	16(1)	21(1)	-4(1)	-3(1)	-8(1)
C(22)	25(1)	37(1)	20(1)	-2(1)	-2(1)	-19(1)

Table 12. Hydrogen coordinates (x 10^4) and isotropic displacement parameters ($Å^2$x 10^3) for **8o**.

	x	y	z	U(eq)
H(3)	9500(30)	7070(30)	3340(20)	49(5)
H(2)	8020(20)	2090(20)	4755(17)	38(5)
H(5)	8561	4845	1253	23
H(6)	9503	3569	-444	27
H(7)	10940	822	-225	29
H(8)	11498	-646	1656	28
H(9)	10622	620	3350	23
H(13)	5411	10274	6794	22
H(15A)	2642	8947	7152	32
H(15B)	3236	9322	8102	32
H(15C)	3660	7537	8069	32
H(17)	5105	2959	6226	21
H(18)	3730	2323	8241	22
H(20)	6169	3837	9482	21
H(21)	7542	4494	7470	20
H(22A)	2751	2859	10412	40
H(22B)	3927	3182	10914	40
H(22C)	4468	1458	10658	40

Table 13. Torsion angles [°] for **8o**.

O(1)-C(10)-C(11)-O(3)	-176.20(9)
O(1)-C(10)-C(11)-C(12)	6.78(16)
O(2)-C(1)-C(2)-C(3)	179.52(11)
O(2)-C(1)-C(2)-C(10)	4.96(19)
O(3)-C(11)-C(12)-O(4)	0.59(16)
O(3)-C(11)-C(12)-C(13)	-178.65(9)
O(4)-C(12)-C(13)-C(14)	177.02(11)
N(1)-N(2)-C(3)-C(2)	2.74(12)
N(1)-N(2)-C(3)-C(16)	-175.64(9)
N(1)-C(1)-C(2)-C(3)	0.57(12)
N(1)-C(1)-C(2)-C(10)	-173.98(10)
N(1)-C(4)-C(5)-C(6)	-177.82(10)
N(1)-C(4)-C(9)-C(8)	176.98(10)
N(2)-N(1)-C(1)-O(2)	-177.99(10)
N(2)-N(1)-C(1)-C(2)	1.05(12)
N(2)-N(1)-C(4)-C(5)	128.78(11)
N(2)-N(1)-C(4)-C(9)	-49.60(14)
N(2)-C(3)-C(16)-C(17)	-42.13(15)
N(2)-C(3)-C(16)-C(21)	135.75(11)
C(1)-N(1)-N(2)-C(3)	-2.39(12)
C(1)-N(1)-C(4)-C(5)	-39.23(16)
C(1)-N(1)-C(4)-C(9)	142.39(12)
C(1)-C(2)-C(3)-N(2)	-2.04(12)
C(1)-C(2)-C(3)-C(16)	176.07(11)
C(1)-C(2)-C(10)-O(1)	151.12(10)
C(1)-C(2)-C(10)-C(11)	-26.88(18)
C(2)-C(3)-C(16)-C(17)	139.92(12)
C(2)-C(3)-C(16)-C(21)	-42.19(18)
C(2)-C(10)-C(11)-O(3)	1.63(19)
C(2)-C(10)-C(11)-C(12)	-175.39(10)
C(3)-C(2)-C(10)-O(1)	-22.30(16)
C(3)-C(2)-C(10)-C(11)	159.71(12)
C(3)-C(16)-C(17)-C(18)	177.94(10)
C(3)-C(16)-C(21)-C(20)	-177.71(10)
C(4)-N(1)-N(2)-C(3)	-172.52(10)
C(4)-N(1)-C(1)-O(2)	-8.82(18)
C(4)-N(1)-C(1)-C(2)	170.22(10)
C(4)-C(5)-C(6)-C(7)	0.60(17)
C(5)-C(4)-C(9)-C(8)	-1.36(16)
C(5)-C(6)-C(7)-C(8)	-0.88(18)
C(6)-C(7)-C(8)-C(9)	0.03(18)
C(7)-C(8)-C(9)-C(4)	1.07(17)
C(9)-C(4)-C(5)-C(6)	0.53(16)
C(10)-O(1)-C(14)-C(13)	1.88(16)
C(10)-O(1)-C(14)-C(15)	179.85(9)
C(10)-C(2)-C(3)-N(2)	172.34(10)
C(10)-C(2)-C(3)-C(16)	-9.6(2)
C(10)-C(11)-C(12)-O(4)	177.84(10)
C(10)-C(11)-C(12)-C(13)	-1.39(16)
C(11)-C(12)-C(13)-C(14)	-3.78(17)
C(12)-C(13)-C(14)-O(1)	3.65(17)
C(12)-C(13)-C(14)-C(15)	-174.01(11)
C(14)-O(1)-C(10)-C(2)	174.66(9)
C(14)-O(1)-C(10)-C(11)	-7.19(16)

C(16)-C(17)-C(18)-C(19)	-0.13(17)
C(17)-C(16)-C(21)-C(20)	0.20(16)
C(17)-C(18)-C(19)-C(20)	0.04(17)
C(17)-C(18)-C(19)-C(22)	-179.87(11)
C(18)-C(19)-C(20)-C(21)	0.16(17)
C(19)-C(20)-C(21)-C(16)	-0.29(17)
C(21)-C(16)-C(17)-C(18)	0.01(16)
C(22)-C(19)-C(20)-C(21)	-179.92(11)

Table 14. Hydrogen bonds for **8o** [Å and °].

D-H...A	d(D-H)	d(H...A)	d(D...A)	<(DHA)
O(3)-H(3)...O(2)	0.91(2)	1.68(2)	2.5803(11)	170(2)
N(2)-H(2)...O(4)#1	0.939(19)	1.769(19)	2.7068(12)	176.3(17)

Symmetry transformations used to generate equivalent atoms:
#1 x,y-1,z

12.3 Crystallographic data for *5-(4-chlorophenyl)-4-(3-hydroxy-6-methyl-4-oxo-4H-pyran-2-yl)-1,2-dihydro-3H-pyrazol-3-one* (**10c**)

Table 15. Crystal data and structure refinement for **10c**.

Identification code	2402116	
Empirical formula	$C_{15}H_{11}ClN_2O_4$	
Formula weight	318.71	
Temperature	100.00(10) K	
Wavelength	1.54184 Å	
Crystal system	Monoclinic	
Space group	P1 21/n 1	
Unit cell dimensions	a = 9.0870(2) Å	a= 90°.
	b = 10.4025(2) Å	b= 105.442(2)°.
	c = 15.4572(3) Å	g = 90°.
Volume	1408.38(5) $Å^3$	
Z	4	
Density (calculated)	1.503 g/cm^3	
Absorption coefficient	2.601 mm^{-1}	
F(000)	656	
Crystal size	0.04 x 0.02 x 0.02 mm^3	
Theta range for data collection	5.131 to 80.673°.	
Index ranges	-11<=h<=11, -12<=k<=13, -19<=l<=19	
Reflections collected	19397	
Independent reflections	3093 [R(int) = 0.0337]	
Observed reflections	2976	
Completeness to theta = 67.684°	100.0 %	
Absorption correction	Semi-empirical from equivalents	
Max. and min. transmission	1.00000 and 0.90723	
Refinement method	Full-matrix least-squares on F^2	
Data / restraints / parameters	3093 / 0 / 213	
Goodness-of-fit on F^2	1.139	
Final R indices [I>2sigma(I)]	R1 = 0.0443, wR2 = 0.1165	
R indices (all data)	R1 = 0.0453, wR2 = 0.1172	
Extinction coefficient	0.0029(4)	
Largest diff. peak and hole	0.386 and -0.802 e.$Å^{-3}$	

Table 16. Atomic coordinates (x 10^4) and equivalent isotropic displacement parameters (Å^2 x 10^3) for **10c** U(eq) is defined as one third of the trace of the orthogonalized U^{ij} tensor.

	x	y	z	U(eq)
Cl(1)	3723(1)	3814(1)	9378(1)	26(1)
O(1)	4300(2)	8606(1)	4302(1)	26(1)
O(2)	2544(2)	6887(1)	3419(1)	30(1)
O(3)	919(2)	4834(1)	2786(1)	24(1)
O(4)	3196(2)	4879(1)	5418(1)	22(1)
N(1)	5411(2)	8145(2)	6586(1)	22(1)
N(2)	5367(2)	8830(2)	5826(1)	22(1)
C(1)	4467(2)	6175(2)	7088(1)	20(1)
C(2)	2998(2)	5978(2)	7178(1)	23(1)
C(3)	2760(2)	5240(2)	7874(1)	22(1)
C(4)	4016(2)	4708(2)	8483(1)	21(1)
C(5)	5488(2)	4879(2)	8404(1)	23(1)
C(6)	5707(2)	5620(2)	7702(1)	21(1)
C(7)	4685(2)	7030(2)	6366(1)	21(1)
C(8)	4158(2)	6960(2)	5427(1)	20(1)
C(9)	4573(2)	8157(2)	5103(1)	22(1)
C(10)	3274(2)	5930(2)	4895(1)	19(1)
C(11)	2544(2)	5922(2)	4000(1)	21(1)
C(12)	1626(2)	4826(2)	3600(1)	21(1)
C(13)	1578(2)	3781(2)	4199(1)	25(1)
C(14)	2345(2)	3839(2)	5073(1)	24(1)
C(15)	2379(3)	2854(2)	5781(1)	32(1)

Table 17. Bond lengths [Å] and angles [°] for **10c**.

Cl(1)-C(4)	1.7465(19)
O(1)-C(9)	1.283(2)
O(2)-H(2)	0.94(3)
O(2)-C(11)	1.346(2)
O(3)-C(12)	1.250(2)
O(4)-C(10)	1.373(2)
O(4)-C(14)	1.355(2)
N(1)-H(1)	0.88(3)
N(1)-N(2)	1.366(2)
N(1)-C(7)	1.333(2)
N(2)-H(2A)	0.96(3)
N(2)-C(9)	1.354(2)
C(1)-C(2)	1.394(3)
C(1)-C(6)	1.391(2)
C(1)-C(7)	1.481(2)
C(2)-H(2B)	0.9500
C(2)-C(3)	1.385(3)
C(3)-H(3)	0.9500
C(3)-C(4)	1.386(3)
C(4)-C(5)	1.386(3)
C(5)-H(5)	0.9500
C(5)-C(6)	1.388(3)
C(6)-H(6)	0.9500
C(7)-C(8)	1.404(2)
C(8)-C(9)	1.430(3)
C(8)-C(10)	1.455(2)
C(10)-C(11)	1.366(2)
C(11)-C(12)	1.451(2)
C(12)-C(13)	1.436(3)
C(13)-H(13)	0.9500
C(13)-C(14)	1.347(3)
C(14)-C(15)	1.492(3)
C(15)-H(15A)	0.9800
C(15)-H(15B)	0.9800
C(15)-H(15C)	0.9800
C(11)-O(2)-H(2)	111(2)
C(14)-O(4)-C(10)	121.19(14)
N(2)-N(1)-H(1)	117.5(18)
C(7)-N(1)-H(1)	132.3(17)
C(7)-N(1)-N(2)	109.59(15)
N(1)-N(2)-H(2A)	123.2(18)
C(9)-N(2)-N(1)	109.10(15)
C(9)-N(2)-H(2A)	125.2(18)
C(2)-C(1)-C(7)	119.20(16)
C(6)-C(1)-C(2)	119.58(17)
C(6)-C(1)-C(7)	121.14(17)
C(1)-C(2)-H(2B)	119.6
C(3)-C(2)-C(1)	120.74(17)
C(3)-C(2)-H(2B)	119.6
C(2)-C(3)-H(3)	120.7
C(2)-C(3)-C(4)	118.54(17)
C(4)-C(3)-H(3)	120.7
C(3)-C(4)-Cl(1)	118.60(15)

C(3)-C(4)-C(5)	121.93(17)
C(5)-C(4)-Cl(1)	119.47(14)
C(4)-C(5)-H(5)	120.6
C(4)-C(5)-C(6)	118.84(17)
C(6)-C(5)-H(5)	120.6
C(1)-C(6)-H(6)	119.8
C(5)-C(6)-C(1)	120.36(17)
C(5)-C(6)-H(6)	119.8
N(1)-C(7)-C(1)	119.09(16)
N(1)-C(7)-C(8)	108.60(16)
C(8)-C(7)-C(1)	132.11(16)
C(7)-C(8)-C(9)	105.51(15)
C(7)-C(8)-C(10)	127.39(16)
C(9)-C(8)-C(10)	126.96(16)
O(1)-C(9)-N(2)	122.11(16)
O(1)-C(9)-C(8)	130.79(17)
N(2)-C(9)-C(8)	107.09(15)
O(4)-C(10)-C(8)	111.16(15)
C(11)-C(10)-O(4)	120.34(16)
C(11)-C(10)-C(8)	128.48(16)
O(2)-C(11)-C(10)	125.80(17)
O(2)-C(11)-C(12)	113.84(15)
C(10)-C(11)-C(12)	120.33(16)
O(3)-C(12)-C(11)	120.42(16)
O(3)-C(12)-C(13)	123.91(17)
C(13)-C(12)-C(11)	115.66(16)
C(12)-C(13)-H(13)	119.4
C(14)-C(13)-C(12)	121.15(17)
C(14)-C(13)-H(13)	119.4
O(4)-C(14)-C(15)	111.21(16)
C(13)-C(14)-O(4)	121.26(17)
C(13)-C(14)-C(15)	127.52(17)
C(14)-C(15)-H(15A)	109.5
C(14)-C(15)-H(15B)	109.5
C(14)-C(15)-H(15C)	109.5
H(15A)-C(15)-H(15B)	109.5
H(15A)-C(15)-H(15C)	109.5
H(15B)-C(15)-H(15C)	109.5

Table 18. Anisotropic displacement parameters ($Å^2 \times 10^3$) for **10c**. The anisotropic displacement factor exponent takes the form: $-2p^2[h^2 a^{*2}U^{11} + ... + 2 h k a^* b^* U^{12}]$

	U^{11}	U^{22}	U^{33}	U^{23}	U^{13}	U^{12}
Cl(1)	32(1)	22(1)	24(1)	4(1)	7(1)	-4(1)
O(1)	40(1)	18(1)	18(1)	2(1)	3(1)	-5(1)
O(2)	43(1)	19(1)	20(1)	4(1)	-3(1)	-7(1)
O(3)	25(1)	23(1)	20(1)	0(1)	-1(1)	-3(1)
O(4)	31(1)	15(1)	18(1)	1(1)	1(1)	-4(1)
N(1)	28(1)	17(1)	16(1)	0(1)	2(1)	-3(1)
N(2)	30(1)	16(1)	18(1)	1(1)	2(1)	-4(1)
C(1)	25(1)	16(1)	17(1)	-1(1)	2(1)	-1(1)
C(2)	23(1)	21(1)	21(1)	-1(1)	1(1)	1(1)
C(3)	22(1)	21(1)	24(1)	-2(1)	5(1)	-2(1)
C(4)	27(1)	16(1)	20(1)	-1(1)	4(1)	-2(1)
C(5)	24(1)	20(1)	21(1)	2(1)	2(1)	2(1)
C(6)	21(1)	19(1)	21(1)	-1(1)	3(1)	-1(1)
C(7)	22(1)	16(1)	22(1)	-1(1)	3(1)	1(1)
C(8)	23(1)	15(1)	19(1)	0(1)	2(1)	-1(1)
C(9)	26(1)	17(1)	19(1)	-2(1)	2(1)	-1(1)
C(10)	24(1)	13(1)	20(1)	1(1)	4(1)	0(1)
C(11)	25(1)	15(1)	21(1)	2(1)	3(1)	1(1)
C(12)	21(1)	18(1)	22(1)	-1(1)	2(1)	1(1)
C(13)	27(1)	19(1)	26(1)	0(1)	2(1)	-5(1)
C(14)	29(1)	15(1)	25(1)	0(1)	3(1)	-4(1)
C(15)	45(1)	21(1)	25(1)	4(1)	3(1)	-10(1)

Table 19. Hydrogen coordinates (x 10^4) and isotropic displacement parameters ($Å^2$x 10^3) for **10c**

	x	y	z	U(eq)
H(2)	3200(40)	7560(30)	3690(20)	56(9)
H(1)	5770(30)	8540(30)	7107(18)	32(6)
H(2A)	5590(30)	9730(30)	5830(19)	43(8)
H(2B)	2150	6354	6758	27
H(3)	1758	5101	7934	27
H(5)	6332	4497	8824	27
H(6)	6709	5748	7641	25
H(13)	995	3037	3975	30
H(15A)	3441	2645	6088	47
H(15B)	1851	2075	5502	47
H(15C)	1867	3194	6215	47

Table 20. Torsion angles [°] for **10c**.

Cl(1)-C(4)-C(5)-C(6)	-178.36(14)
O(2)-C(11)-C(12)-O(3)	-0.3(3)
O(2)-C(11)-C(12)-C(13)	-179.20(17)
O(3)-C(12)-C(13)-C(14)	-178.83(19)
O(4)-C(10)-C(11)-O(2)	-179.54(17)
O(4)-C(10)-C(11)-C(12)	2.6(3)
N(1)-N(2)-C(9)-O(1)	176.90(17)
N(1)-N(2)-C(9)-C(8)	-2.7(2)
N(1)-C(7)-C(8)-C(9)	-2.4(2)
N(1)-C(7)-C(8)-C(10)	-178.26(17)
N(2)-N(1)-C(7)-C(1)	-174.57(16)
N(2)-N(1)-C(7)-C(8)	0.9(2)
C(1)-C(2)-C(3)-C(4)	0.4(3)
C(1)-C(7)-C(8)-C(9)	172.17(19)
C(1)-C(7)-C(8)-C(10)	-3.6(3)
C(2)-C(1)-C(6)-C(5)	-0.3(3)
C(2)-C(1)-C(7)-N(1)	112.1(2)
C(2)-C(1)-C(7)-C(8)	-62.1(3)
C(2)-C(3)-C(4)-Cl(1)	178.26(14)
C(2)-C(3)-C(4)-C(5)	-0.9(3)
C(3)-C(4)-C(5)-C(6)	0.8(3)
C(4)-C(5)-C(6)-C(1)	-0.2(3)
C(6)-C(1)-C(2)-C(3)	0.2(3)
C(6)-C(1)-C(7)-N(1)	-64.6(2)
C(6)-C(1)-C(7)-C(8)	121.2(2)
C(7)-N(1)-N(2)-C(9)	1.2(2)
C(7)-C(1)-C(2)-C(3)	-176.56(17)
C(7)-C(1)-C(6)-C(5)	176.38(16)
C(7)-C(8)-C(9)-O(1)	-176.4(2)
C(7)-C(8)-C(9)-N(2)	3.1(2)
C(7)-C(8)-C(10)-O(4)	-9.9(3)
C(7)-C(8)-C(10)-C(11)	168.58(19)
C(8)-C(10)-C(11)-O(2)	2.1(3)
C(8)-C(10)-C(11)-C(12)	-175.76(18)
C(9)-C(8)-C(10)-O(4)	175.14(17)
C(9)-C(8)-C(10)-C(11)	-6.4(3)
C(10)-O(4)-C(14)-C(13)	1.9(3)
C(10)-O(4)-C(14)-C(15)	-177.09(17)
C(10)-C(8)-C(9)-O(1)	-0.6(3)
C(10)-C(8)-C(9)-N(2)	178.97(18)
C(10)-C(11)-C(12)-O(3)	177.78(18)
C(10)-C(11)-C(12)-C(13)	-1.1(3)
C(11)-C(12)-C(13)-C(14)	0.0(3)
C(12)-C(13)-C(14)-O(4)	-0.4(3)
C(12)-C(13)-C(14)-C(15)	178.4(2)
C(14)-O(4)-C(10)-C(8)	175.59(16)
C(14)-O(4)-C(10)-C(11)	-3.0(3)

Table 21. Hydrogen bonds for **10c** [Å and °].

D-H...A	d(D-H)	d(H...A)	d(D...A)	<(DHA)
O(2)-H(2)...O(1)	0.94(3)	1.60(3)	2.5396(19)	171(3)
N(1)-H(1)...O(2)#1	0.88(3)	2.27(3)	2.977(2)	136(2)
N(1)-H(1)...O(3)#1	0.88(3)	1.98(3)	2.761(2)	147(2)
N(2)-H(2A)...O(1)#2	0.96(3)	1.75(3)	2.697(2)	170(3)

Symmetry transformations used to generate equivalent atoms:
#1 x+1/2,-y+3/2,z+1/2   #2 -x+1,-y+2,-z+1

12.4 Crystallographic data for *3-(4-chlorophenyl)-4-(3-hydroxy-6-methyl-4-oxo-4H-pyran-2-yl)isoxazol-5(2H)-one* (**11**)

Table 22. Crystal data and structure refinement for **11**.

Identification code	2402118	
Empirical formula	$C_{15}H_{12}ClNO_6$	
Formula weight	337.71	
Temperature	100.00(10) K	
Wavelength	1.54184 Å	
Crystal system	Triclinic	
Space group	P-1	
Unit cell dimensions	a = 6.96396(11) Å	a= 98.0696(12)°.
	b = 7.24691(10) Å	b= 98.3741(12)°.
	c = 14.1777(2) Å	g = 93.6596(12)°.
Volume	698.274(18) Å^3	
Z	2	
Density (calculated)	1.606 g/cm^3	
Absorption coefficient	2.748 mm^{-1}	
F(000)	348	
Crystal size	0.04 x 0.03 x 0.02 mm^3	
Theta range for data collection	3.188 to 80.744°.	
Index ranges	-8<=h<=8, -9<=k<=9, -18<=l<=17	
Reflections collected	15335	
Independent reflections	3032 [R(int) = 0.0436]	
Observed reflections	2866	
Completeness to theta = 67.684°	99.9 %	
Absorption correction	Semi-empirical from equivalents	
Max. and min. transmission	1.00000 and 0.82232	
Refinement method	Full-matrix least-squares on F^2	
Data / restraints / parameters	3032 / 0 / 229	
Goodness-of-fit on F^2	1.111	
Final R indices [I>2sigma(I)]	R1 = 0.0381, wR2 = 0.1048	
R indices (all data)	R1 = 0.0392, wR2 = 0.1058	
Largest diff. peak and hole	0.354 and -0.400 e.Å^{-3}	

Table 23. Atomic coordinates (x 10^4) and equivalent isotropic displacement parameters ($Å^2$x 10^3) for **11**. U(eq) is defined as one third of the trace of the orthogonalized U^{ij} tensor.

	x	y	z	U(eq)
Cl(1)	13052(1)	1727(1)	5592(1)	33(1)
O(1)	10725(2)	8851(2)	1848(1)	23(1)
O(2)	7902(2)	8854(2)	867(1)	25(1)
O(3)	6731(2)	3942(1)	2310(1)	19(1)
O(4)	4609(2)	7186(2)	803(1)	24(1)
O(5)	1752(2)	4600(2)	589(1)	23(1)
N(1)	11590(2)	7823(2)	2565(1)	22(1)
C(1)	10598(2)	3342(2)	3149(1)	21(1)
C(2)	11282(2)	2234(2)	3829(1)	23(1)
C(3)	12222(2)	3097(2)	4726(1)	23(1)
C(4)	12513(2)	5028(2)	4956(1)	24(1)
C(5)	11826(2)	6121(2)	4270(1)	22(1)
C(6)	10850(2)	5287(2)	3364(1)	19(1)
C(7)	10252(2)	6518(2)	2638(1)	19(1)
C(8)	8469(2)	6594(2)	1995(1)	19(1)
C(9)	8879(2)	8104(2)	1508(1)	21(1)
C(10)	6660(2)	5444(2)	1834(1)	18(1)
C(11)	4926(2)	5691(2)	1259(1)	19(1)
C(12)	3360(2)	4307(2)	1133(1)	20(1)
C(13)	3587(2)	2696(2)	1580(1)	20(1)
C(14)	5255(2)	2576(2)	2178(1)	20(1)
C(15)	5686(2)	1063(2)	2765(1)	23(1)
O(6)	-1245(2)	2398(2)	551(1)	24(1)

Table 24. Bond lengths [Å] and angles [°] for **11**.

Cl(1)-C(3)	1.7416(15)
O(1)-N(1)	1.4299(16)
O(1)-C(9)	1.3545(19)
O(2)-C(9)	1.2613(19)
O(3)-C(10)	1.3592(16)
O(3)-C(14)	1.3537(18)
O(4)-H(4)	0.95(3)
O(4)-C(11)	1.3514(17)
O(5)-H(5)	0.95(3)
O(5)-C(12)	1.3109(18)
N(1)-C(7)	1.310(2)
C(1)-H(1)	0.9500
C(1)-C(2)	1.390(2)
C(1)-C(6)	1.394(2)
C(2)-H(2)	0.9500
C(2)-C(3)	1.387(2)
C(3)-C(4)	1.386(2)
C(4)-H(4A)	0.9500
C(4)-C(5)	1.390(2)
C(5)-H(5A)	0.97(2)
C(5)-C(6)	1.398(2)
C(6)-C(7)	1.4859(19)
C(7)-C(8)	1.438(2)
C(8)-C(9)	1.408(2)
C(8)-C(10)	1.436(2)
C(10)-C(11)	1.392(2)
C(11)-C(12)	1.407(2)
C(12)-C(13)	1.412(2)
C(13)-H(13)	0.9500
C(13)-C(14)	1.349(2)
C(14)-C(15)	1.4874(19)
C(15)-H(15A)	0.9800
C(15)-H(15B)	0.9800
C(15)-H(15C)	0.9800
O(6)-H(6A)	0.88(3)
O(6)-H(6B)	0.86(3)
C(9)-O(1)-N(1)	109.35(11)
C(14)-O(3)-C(10)	122.86(12)
C(11)-O(4)-H(4)	110.8(17)
C(12)-O(5)-H(5)	113.3(17)
C(7)-N(1)-O(1)	105.94(12)
C(2)-C(1)-H(1)	119.6
C(2)-C(1)-C(6)	120.75(14)
C(6)-C(1)-H(1)	119.6
C(1)-C(2)-H(2)	120.6
C(3)-C(2)-C(1)	118.83(14)
C(3)-C(2)-H(2)	120.6
C(2)-C(3)-Cl(1)	119.34(12)
C(4)-C(3)-Cl(1)	119.00(12)
C(4)-C(3)-C(2)	121.67(14)
C(3)-C(4)-H(4A)	120.5
C(3)-C(4)-C(5)	118.97(14)
C(5)-C(4)-H(4A)	120.5

C(4)-C(5)-H(5A)	119.4(12)
C(4)-C(5)-C(6)	120.56(14)
C(6)-C(5)-H(5A)	120.1(12)
C(1)-C(6)-C(5)	119.21(14)
C(1)-C(6)-C(7)	122.34(13)
C(5)-C(6)-C(7)	118.29(13)
N(1)-C(7)-C(6)	114.79(13)
N(1)-C(7)-C(8)	112.20(13)
C(8)-C(7)-C(6)	133.01(13)
C(9)-C(8)-C(7)	103.70(12)
C(9)-C(8)-C(10)	125.47(13)
C(10)-C(8)-C(7)	130.82(13)
O(1)-C(9)-C(8)	108.81(13)
O(2)-C(9)-O(1)	117.50(13)
O(2)-C(9)-C(8)	133.68(14)
O(3)-C(10)-C(8)	113.43(12)
O(3)-C(10)-C(11)	118.62(13)
C(11)-C(10)-C(8)	127.94(13)
O(4)-C(11)-C(10)	124.79(13)
O(4)-C(11)-C(12)	116.28(13)
C(10)-C(11)-C(12)	118.93(13)
O(5)-C(12)-C(11)	116.88(13)
O(5)-C(12)-C(13)	123.57(13)
C(11)-C(12)-C(13)	119.55(13)
C(12)-C(13)-H(13)	120.4
C(14)-C(13)-C(12)	119.16(13)
C(14)-C(13)-H(13)	120.4
O(3)-C(14)-C(15)	112.99(12)
C(13)-C(14)-O(3)	120.48(13)
C(13)-C(14)-C(15)	126.51(13)
C(14)-C(15)-H(15A)	109.5
C(14)-C(15)-H(15B)	109.5
C(14)-C(15)-H(15C)	109.5
H(15A)-C(15)-H(15B)	109.5
H(15A)-C(15)-H(15C)	109.5
H(15B)-C(15)-H(15C)	109.5
H(6A)-O(6)-H(6B)	108(2)

Table 25. Anisotropic displacement parameters (Å^{2}x 10^3) for **11**. The anisotropic displacement factor exponent takes the form: -2p^2[h^2 a*^{2}U^{11} + ... + 2 h k a* b* U^{12}]

	U^{11}	U^{22}	U^{33}	U^{23}	U^{13}	U^{12}
Cl(1)	39(1)	32(1)	29(1)	14(1)	-1(1)	10(1)
O(1)	22(1)	19(1)	28(1)	10(1)	-1(1)	-3(1)
O(2)	23(1)	22(1)	31(1)	14(1)	-1(1)	-1(1)
O(3)	19(1)	16(1)	23(1)	8(1)	0(1)	-1(1)
O(4)	21(1)	20(1)	31(1)	13(1)	-3(1)	-2(1)
O(5)	19(1)	21(1)	27(1)	10(1)	-4(1)	-2(1)
N(1)	23(1)	20(1)	25(1)	9(1)	-1(1)	0(1)
C(1)	19(1)	20(1)	22(1)	4(1)	1(1)	1(1)
C(2)	23(1)	19(1)	26(1)	6(1)	4(1)	3(1)
C(3)	22(1)	25(1)	24(1)	10(1)	2(1)	6(1)
C(4)	23(1)	26(1)	22(1)	4(1)	-1(1)	2(1)
C(5)	21(1)	20(1)	25(1)	4(1)	1(1)	1(1)
C(6)	16(1)	20(1)	22(1)	6(1)	2(1)	2(1)
C(7)	19(1)	16(1)	21(1)	3(1)	2(1)	1(1)
C(8)	20(1)	17(1)	20(1)	5(1)	1(1)	1(1)
C(9)	22(1)	16(1)	24(1)	4(1)	2(1)	-1(1)
C(10)	22(1)	13(1)	20(1)	5(1)	2(1)	1(1)
C(11)	22(1)	16(1)	21(1)	6(1)	2(1)	2(1)
C(12)	20(1)	19(1)	19(1)	4(1)	1(1)	1(1)
C(13)	20(1)	16(1)	22(1)	4(1)	1(1)	-2(1)
C(14)	20(1)	16(1)	23(1)	5(1)	3(1)	-1(1)
C(15)	23(1)	18(1)	28(1)	10(1)	0(1)	0(1)
O(6)	23(1)	20(1)	30(1)	11(1)	-4(1)	-2(1)

Table 26. Hydrogen coordinates (x 10^4) and isotropic displacement parameters ($Å^2$x 10^3) for **11**.

	x	y	z	U(eq)
H(4)	5800(40)	7900(40)	800(20)	52(7)
H(5)	710(40)	3670(40)	570(20)	49(7)
H(1)	9952	2766	2531	25
H(2)	11108	908	3680	27
H(4A)	13171	5595	5573	29
H(5A)	12000(30)	7470(30)	4432(15)	25(5)
H(13)	2578	1708	1462	24
H(15A)	6984	666	2695	34
H(15B)	4710	-3	2542	34
H(15C)	5648	1529	3445	34
H(6A)	-2140(40)	2600(40)	90(20)	45(7)
H(6B)	-1430(40)	1240(40)	625(19)	49(7)

Table 27. Torsion angles [°] for **11**.

Cl(1)-C(3)-C(4)-C(5)	-179.09(12)
O(1)-N(1)-C(7)-C(6)	-179.26(11)
O(1)-N(1)-C(7)-C(8)	-0.08(16)
O(3)-C(10)-C(11)-O(4)	174.94(13)
O(3)-C(10)-C(11)-C(12)	-4.8(2)
O(4)-C(11)-C(12)-O(5)	-0.1(2)
O(4)-C(11)-C(12)-C(13)	179.47(13)
O(5)-C(12)-C(13)-C(14)	-175.72(14)
N(1)-O(1)-C(9)-O(2)	179.89(13)
N(1)-O(1)-C(9)-C(8)	0.70(16)
N(1)-C(7)-C(8)-C(9)	0.48(17)
N(1)-C(7)-C(8)-C(10)	179.51(15)
C(1)-C(2)-C(3)-Cl(1)	178.98(11)
C(1)-C(2)-C(3)-C(4)	-0.7(2)
C(1)-C(6)-C(7)-N(1)	-130.10(15)
C(1)-C(6)-C(7)-C(8)	50.9(2)
C(2)-C(1)-C(6)-C(5)	0.9(2)
C(2)-C(1)-C(6)-C(7)	176.07(13)
C(2)-C(3)-C(4)-C(5)	0.6(2)
C(3)-C(4)-C(5)-C(6)	0.3(2)
C(4)-C(5)-C(6)-C(1)	-1.0(2)
C(4)-C(5)-C(6)-C(7)	-176.37(13)
C(5)-C(6)-C(7)-N(1)	45.14(19)
C(5)-C(6)-C(7)-C(8)	-133.83(17)
C(6)-C(1)-C(2)-C(3)	0.0(2)
C(6)-C(7)-C(8)-C(9)	179.47(15)
C(6)-C(7)-C(8)-C(10)	-1.5(3)
C(7)-C(8)-C(9)-O(1)	-0.71(16)
C(7)-C(8)-C(9)-O(2)	-179.71(17)
C(7)-C(8)-C(10)-O(3)	-6.7(2)
C(7)-C(8)-C(10)-C(11)	173.87(15)
C(8)-C(10)-C(11)-O(4)	-5.6(2)
C(8)-C(10)-C(11)-C(12)	174.61(14)
C(9)-O(1)-N(1)-C(7)	-0.39(16)
C(9)-C(8)-C(10)-O(3)	172.17(13)
C(9)-C(8)-C(10)-C(11)	-7.3(2)
C(10)-O(3)-C(14)-C(13)	-2.6(2)
C(10)-O(3)-C(14)-C(15)	178.91(12)
C(10)-C(8)-C(9)-O(1)	-179.81(13)
C(10)-C(8)-C(9)-O(2)	1.2(3)
C(10)-C(11)-C(12)-O(5)	179.74(13)
C(10)-C(11)-C(12)-C(13)	-0.7(2)
C(11)-C(12)-C(13)-C(14)	4.8(2)
C(12)-C(13)-C(14)-O(3)	-3.2(2)
C(12)-C(13)-C(14)-C(15)	175.02(14)
C(14)-O(3)-C(10)-C(8)	-172.80(12)
C(14)-O(3)-C(10)-C(11)	6.7(2)

Table 28. Hydrogen bonds for **11** [Å and °].

D-H...A	d(D-H)	d(H...A)	d(D...A)	<(DHA)
O(4)-H(4)...O(2)	0.95(3)	1.56(3)	2.5062(15)	172(3)
O(5)-H(5)...O(6)	0.95(3)	1.59(3)	2.5344(15)	171(3)
O(6)-H(6A)...O(4)#1	0.88(3)	2.01(3)	2.8651(15)	167(2)
O(6)-H(6A)...O(5)#1	0.88(3)	2.38(3)	2.8966(15)	118(2)
O(6)-H(6B)...O(2)#2	0.86(3)	1.85(3)	2.7121(15)	172(3)

Symmetry transformations used to generate equivalent atoms:
#1 -x,-y+1,-z #2 x-1,y-1,z

12.5 Crystallographic data for *6-methyl-4-oxo-2-(1-phenyl-5-(pivaloyloxy)-3-(p-tolyl)-1H-pyrazol-4-yl)-4H-pyran-3-yl pivalate* (**13**)

Table 29. Crystal data and structure refinement for **13**.

Identification code	2402120	
Empirical formula	$C_{32}H_{34}N_2O_6$	
Formula weight	542.61	
Temperature	100.00(13) K	
Wavelength	1.54184 Å	
Crystal system	Monoclinic	
Space group	$P2_1/c$	
Unit cell dimensions	a = 9.40540(10) Å	a= 90°.
	b = 15.64300(10) Å	b= 95.1000(10)°.
	c = 20.97630(10) Å	g = 90°.
Volume	3074.00(4) Å^3	
Z	4	
Density (calculated)	1.172 Mg/m^3	
Absorption coefficient	0.660 mm^{-1}	
F(000)	1152	
Crystal size	0.41 x 0.28 x 0.14 mm^3	
Theta range for data collection	3.530 to 79.903°.	
Index ranges	-9<=h<=11, -19<=k<=19, -26<=l<=26	
Reflections collected	78608	
Independent reflections	6677 [R(int) = 0.0672]	
Completeness to theta = 67.684°	100.0 %	
Absorption correction	Gaussian	
Max. and min. transmission	1.000 and 0.344	
Refinement method	Full-matrix least-squares on F^2	
Data / restraints / parameters	6677 / 19 / 388	
Goodness-of-fit on F^2	1.033	
Final R indices [I>2sigma(I)]	R1 = 0.0484, wR2 = 0.1317	
R indices (all data)	R1 = 0.0506, wR2 = 0.1338	
Largest diff. peak and hole	0.322 and -0.313 e.Å^{-3}	

Table 30. Atomic coordinates (x 10^4) and equivalent isotropic displacement parameters ($Å^2$x 10^3) for **13**. U(eq) is defined as one third of the trace of the orthogonalized U^{ij} tensor.

	x	y	z	U(eq)
O(1)	9459(1)	3902(1)	6134(1)	27(1)
O(2)	8835(1)	5006(1)	7602(1)	28(1)
O(3)	8583(1)	3729(1)	8070(1)	44(1)
O(4)	11756(1)	4701(1)	7758(1)	34(1)
O(5)	6894(1)	5934(1)	6669(1)	27(1)
O(6)	6949(1)	6338(1)	5640(1)	41(1)
N(1)	5116(1)	4966(1)	6242(1)	27(1)
N(2)	4960(1)	4128(1)	6065(1)	28(1)
C(1)	7261(1)	4396(1)	6444(1)	25(1)
C(2)	6474(1)	5136(1)	6467(1)	26(1)
C(3)	6248(1)	3782(1)	6188(1)	26(1)
C(4)	8798(1)	4312(1)	6601(1)	25(1)
C(6)	9544(1)	4594(1)	7136(1)	26(1)
C(7)	11083(1)	4471(1)	7258(1)	27(1)
C(8)	11699(1)	4032(1)	6740(1)	30(1)
C(9)	10903(1)	3764(1)	6218(1)	29(1)
C(10)	8405(1)	4486(1)	8073(1)	31(1)
C(11)	7666(2)	4997(1)	8564(1)	36(1)
C(12)	6345(2)	5428(1)	8221(1)	55(1)
C(13)	8689(2)	5676(1)	8859(1)	57(1)
C(14)	7214(2)	4384(1)	9073(1)	53(1)
C(15)	11407(2)	3296(1)	5662(1)	40(1)
C(16)	7129(1)	6521(1)	6192(1)	29(1)
C(17)	7607(1)	7371(1)	6489(1)	32(1)
C(18)	8853(2)	7216(1)	6998(1)	38(1)
C(19)	8059(2)	7954(1)	5958(1)	41(1)
C(20)	6340(2)	7770(1)	6798(1)	42(1)
C(21)	6441(1)	2861(1)	6078(1)	27(1)
C(22)	5674(1)	2463(1)	5562(1)	28(1)
C(23)	5814(1)	1587(1)	5470(1)	29(1)
C(24)	6717(1)	1094(1)	5884(1)	28(1)
C(25)	7480(1)	1498(1)	6399(1)	30(1)
C(26)	7353(1)	2372(1)	6495(1)	29(1)
C(27)	6860(2)	143(1)	5789(1)	34(1)
C(28A)	3920(20)	5523(16)	6123(11)	29(1)
C(29A)	3149(18)	5469(11)	5532(9)	35(1)
C(30A)	2015(15)	6027(10)	5395(6)	42(1)
C(31A)	1683(12)	6631(9)	5840(6)	42(1)
C(32A)	2432(17)	6661(11)	6436(7)	40(1)
C(33A)	3590(40)	6120(20)	6579(11)	34(1)
C(28B)	3988(7)	5573(5)	6122(4)	29(1)
C(29B)	3260(6)	5609(4)	5520(3)	35(1)
C(30B)	2228(6)	6237(5)	5399(2)	42(1)
C(31B)	1948(7)	6815(4)	5871(2)	42(1)
C(32B)	2662(7)	6761(4)	6474(2)	40(1)
C(33B)	3681(11)	6124(7)	6609(4)	34(1)

Table 31. Bond lengths [Å] and angles [°] for **13**.

O(1)-C(4)	1.3662(14)
O(1)-C(9)	1.3710(15)
O(2)-C(6)	1.3895(14)
O(2)-C(10)	1.3695(15)
O(3)-C(10)	1.1965(17)
O(4)-C(7)	1.2308(16)
O(5)-C(2)	1.3661(14)
O(5)-C(16)	1.3913(15)
O(6)-C(16)	1.1890(17)
N(1)-N(2)	1.3671(14)
N(1)-C(2)	1.3480(16)
N(1)-C(28A)	1.429(3)
N(1)-C(28B)	1.4294(18)
N(2)-C(3)	1.3310(16)
C(1)-C(2)	1.3769(16)
C(1)-C(3)	1.4236(16)
C(1)-C(4)	1.4600(16)
C(3)-C(21)	1.4715(16)
C(4)-C(6)	1.3427(17)
C(6)-C(7)	1.4602(17)
C(7)-C(8)	1.4482(18)
C(8)-H(8)	0.9500
C(8)-C(9)	1.3383(19)
C(9)-C(15)	1.4896(18)
C(10)-C(11)	1.5193(18)
C(11)-C(12)	1.535(2)
C(11)-C(13)	1.527(2)
C(11)-C(14)	1.525(2)
C(12)-H(12A)	0.9800
C(12)-H(12B)	0.9800
C(12)-H(12C)	0.9800
C(13)-H(13A)	0.9800
C(13)-H(13B)	0.9800
C(13)-H(13C)	0.9800
C(14)-H(14A)	0.9800
C(14)-H(14B)	0.9800
C(14)-H(14C)	0.9800
C(15)-H(15A)	0.9800
C(15)-H(15B)	0.9800
C(15)-H(15C)	0.9800
C(16)-C(17)	1.5187(18)
C(17)-C(18)	1.533(2)
C(17)-C(19)	1.530(2)
C(17)-C(20)	1.5383(19)
C(18)-H(18A)	0.9800
C(18)-H(18B)	0.9800
C(18)-H(18C)	0.9800
C(19)-H(19A)	0.9800
C(19)-H(19B)	0.9800
C(19)-H(19C)	0.9800
C(20)-H(20A)	0.9800
C(20)-H(20B)	0.9800
C(20)-H(20C)	0.9800
C(21)-C(22)	1.3935(17)

C(21)-C(26)	1.3974(17)
C(22)-H(22)	0.9500
C(22)-C(23)	1.3920(17)
C(23)-H(23)	0.9500
C(23)-C(24)	1.3931(18)
C(24)-C(25)	1.3934(18)
C(24)-C(27)	1.5092(17)
C(25)-H(25)	0.9500
C(25)-C(26)	1.3903(17)
C(26)-H(26)	0.9500
C(27)-H(27A)	0.9800
C(27)-H(27B)	0.9800
C(27)-H(27C)	0.9800
C(28A)-C(29A)	1.382(3)
C(28A)-C(33A)	1.386(3)
C(29A)-H(29A)	0.9500
C(29A)-C(30A)	1.389(3)
C(30A)-H(30A)	0.9500
C(30A)-C(31A)	1.383(4)
C(31A)-H(31A)	0.9500
C(31A)-C(32A)	1.381(4)
C(32A)-H(32A)	0.9500
C(32A)-C(33A)	1.394(3)
C(33A)-H(33A)	0.9500
C(28B)-C(29B)	1.382(2)
C(28B)-C(33B)	1.386(2)
C(29B)-H(29B)	0.9500
C(29B)-C(30B)	1.389(2)
C(30B)-H(30B)	0.9500
C(30B)-C(31B)	1.383(3)
C(31B)-H(31B)	0.9500
C(31B)-C(32B)	1.381(3)
C(32B)-H(32B)	0.9500
C(32B)-C(33B)	1.394(3)
C(33B)-H(33B)	0.9500

C(4)-O(1)-C(9)	119.43(10)
C(10)-O(2)-C(6)	115.05(9)
C(2)-O(5)-C(16)	116.14(10)
N(2)-N(1)-C(28A)	118.3(12)
N(2)-N(1)-C(28B)	121.9(4)
C(2)-N(1)-N(2)	111.05(10)
C(2)-N(1)-C(28A)	130.4(12)
C(2)-N(1)-C(28B)	126.6(4)
C(3)-N(2)-N(1)	105.30(10)
C(2)-C(1)-C(3)	103.70(10)
C(2)-C(1)-C(4)	126.24(11)
C(3)-C(1)-C(4)	129.88(11)
O(5)-C(2)-C(1)	129.66(11)
N(1)-C(2)-O(5)	121.81(10)
N(1)-C(2)-C(1)	108.53(10)
N(2)-C(3)-C(1)	111.43(10)
N(2)-C(3)-C(21)	119.36(11)
C(1)-C(3)-C(21)	129.15(11)
O(1)-C(4)-C(1)	112.58(10)
C(6)-C(4)-O(1)	121.03(11)

C(6)-C(4)-C(1)	126.39(11)
O(2)-C(6)-C(7)	117.90(11)
C(4)-C(6)-O(2)	119.45(11)
C(4)-C(6)-C(7)	122.64(11)
O(4)-C(7)-C(6)	122.52(12)
O(4)-C(7)-C(8)	124.67(12)
C(8)-C(7)-C(6)	112.79(11)
C(7)-C(8)-H(8)	119.0
C(9)-C(8)-C(7)	122.06(11)
C(9)-C(8)-H(8)	119.0
O(1)-C(9)-C(15)	111.01(11)
C(8)-C(9)-O(1)	122.03(11)
C(8)-C(9)-C(15)	126.96(12)
O(2)-C(10)-C(11)	111.03(11)
O(3)-C(10)-O(2)	122.30(12)
O(3)-C(10)-C(11)	126.65(12)
C(10)-C(11)-C(12)	108.27(12)
C(10)-C(11)-C(13)	109.34(12)
C(10)-C(11)-C(14)	108.46(12)
C(13)-C(11)-C(12)	109.86(15)
C(14)-C(11)-C(12)	109.87(14)
C(14)-C(11)-C(13)	110.98(14)
C(11)-C(12)-H(12A)	109.5
C(11)-C(12)-H(12B)	109.5
C(11)-C(12)-H(12C)	109.5
H(12A)-C(12)-H(12B)	109.5
H(12A)-C(12)-H(12C)	109.5
H(12B)-C(12)-H(12C)	109.5
C(11)-C(13)-H(13A)	109.5
C(11)-C(13)-H(13B)	109.5
C(11)-C(13)-H(13C)	109.5
H(13A)-C(13)-H(13B)	109.5
H(13A)-C(13)-H(13C)	109.5
H(13B)-C(13)-H(13C)	109.5
C(11)-C(14)-H(14A)	109.5
C(11)-C(14)-H(14B)	109.5
C(11)-C(14)-H(14C)	109.5
H(14A)-C(14)-H(14B)	109.5
H(14A)-C(14)-H(14C)	109.5
H(14B)-C(14)-H(14C)	109.5
C(9)-C(15)-H(15A)	109.5
C(9)-C(15)-H(15B)	109.5
C(9)-C(15)-H(15C)	109.5
H(15A)-C(15)-H(15B)	109.5
H(15A)-C(15)-H(15C)	109.5
H(15B)-C(15)-H(15C)	109.5
O(5)-C(16)-C(17)	110.00(11)
O(6)-C(16)-O(5)	121.53(12)
O(6)-C(16)-C(17)	128.47(12)
C(16)-C(17)-C(18)	109.16(11)
C(16)-C(17)-C(19)	108.26(12)
C(16)-C(17)-C(20)	108.48(11)
C(18)-C(17)-C(20)	110.11(12)
C(19)-C(17)-C(18)	110.87(12)
C(19)-C(17)-C(20)	109.89(12)
C(17)-C(18)-H(18A)	109.5

C(17)-C(18)-H(18B)	109.5
C(17)-C(18)-H(18C)	109.5
H(18A)-C(18)-H(18B)	109.5
H(18A)-C(18)-H(18C)	109.5
H(18B)-C(18)-H(18C)	109.5
C(17)-C(19)-H(19A)	109.5
C(17)-C(19)-H(19B)	109.5
C(17)-C(19)-H(19C)	109.5
H(19A)-C(19)-H(19B)	109.5
H(19A)-C(19)-H(19C)	109.5
H(19B)-C(19)-H(19C)	109.5
C(17)-C(20)-H(20A)	109.5
C(17)-C(20)-H(20B)	109.5
C(17)-C(20)-H(20C)	109.5
H(20A)-C(20)-H(20B)	109.5
H(20A)-C(20)-H(20C)	109.5
H(20B)-C(20)-H(20C)	109.5
C(22)-C(21)-C(3)	119.77(11)
C(22)-C(21)-C(26)	119.24(11)
C(26)-C(21)-C(3)	120.97(11)
C(21)-C(22)-H(22)	120.0
C(23)-C(22)-C(21)	119.97(12)
C(23)-C(22)-H(22)	120.0
C(22)-C(23)-H(23)	119.4
C(22)-C(23)-C(24)	121.17(12)
C(24)-C(23)-H(23)	119.4
C(23)-C(24)-C(25)	118.50(11)
C(23)-C(24)-C(27)	121.29(12)
C(25)-C(24)-C(27)	120.20(12)
C(24)-C(25)-H(25)	119.6
C(26)-C(25)-C(24)	120.86(12)
C(26)-C(25)-H(25)	119.6
C(21)-C(26)-H(26)	119.9
C(25)-C(26)-C(21)	120.26(12)
C(25)-C(26)-H(26)	119.9
C(24)-C(27)-H(27A)	109.5
C(24)-C(27)-H(27B)	109.5
C(24)-C(27)-H(27C)	109.5
H(27A)-C(27)-H(27B)	109.5
H(27A)-C(27)-H(27C)	109.5
H(27B)-C(27)-H(27C)	109.5
C(29A)-C(28A)-N(1)	117.5(13)
C(29A)-C(28A)-C(33A)	122.0(2)
C(33A)-C(28A)-N(1)	120.5(14)
C(28A)-C(29A)-H(29A)	120.7
C(28A)-C(29A)-C(30A)	118.5(2)
C(30A)-C(29A)-H(29A)	120.7
C(29A)-C(30A)-H(30A)	119.8
C(31A)-C(30A)-C(29A)	120.4(2)
C(31A)-C(30A)-H(30A)	119.8
C(30A)-C(31A)-H(31A)	119.8
C(32A)-C(31A)-C(30A)	120.4(2)
C(32A)-C(31A)-H(31A)	119.8
C(31A)-C(32A)-H(32A)	120.0
C(31A)-C(32A)-C(33A)	120.0(2)
C(33A)-C(32A)-H(32A)	120.0

C(28A)-C(33A)-C(32A)	118.6(3)
C(28A)-C(33A)-H(33A)	120.7
C(32A)-C(33A)-H(33A)	120.7
C(29B)-C(28B)-N(1)	119.3(4)
C(29B)-C(28B)-C(33B)	121.89(14)
C(33B)-C(28B)-N(1)	118.8(4)
C(28B)-C(29B)-H(29B)	120.7
C(28B)-C(29B)-C(30B)	118.53(16)
C(30B)-C(29B)-H(29B)	120.7
C(29B)-C(30B)-H(30B)	119.8
C(31B)-C(30B)-C(29B)	120.43(17)
C(31B)-C(30B)-H(30B)	119.8
C(30B)-C(31B)-H(31B)	119.8
C(32B)-C(31B)-C(30B)	120.43(15)
C(32B)-C(31B)-H(31B)	119.8
C(31B)-C(32B)-H(32B)	120.0
C(31B)-C(32B)-C(33B)	119.99(16)
C(33B)-C(32B)-H(32B)	120.0
C(28B)-C(33B)-C(32B)	118.6(2)
C(28B)-C(33B)-H(33B)	120.7
C(32B)-C(33B)-H(33B)	120.7

Table 32. Anisotropic displacement parameters (Å^{2}x 10^3) for **13**. The anisotropic displacement factor exponent takes the form: -2p^2[h^2 a*^{2}U^{11} + ... + 2h k a* b* U^{12}]

	U^{11}	U^{22}	U^{33}	U^{23}	U^{13}	U^{12}
O(1)	23(1)	27(1)	32(1)	-4(1)	4(1)	1(1)
O(2)	30(1)	23(1)	30(1)	-1(1)	6(1)	1(1)
O(3)	55(1)	27(1)	53(1)	7(1)	22(1)	4(1)
O(4)	32(1)	33(1)	36(1)	-4(1)	-4(1)	1(1)
O(5)	28(1)	19(1)	34(1)	-2(1)	2(1)	0(1)
O(6)	54(1)	34(1)	36(1)	-1(1)	10(1)	-7(1)
N(1)	22(1)	22(1)	36(1)	-2(1)	2(1)	1(1)
N(2)	26(1)	22(1)	36(1)	-3(1)	3(1)	0(1)
C(1)	24(1)	21(1)	30(1)	-1(1)	3(1)	0(1)
C(2)	25(1)	21(1)	31(1)	-2(1)	3(1)	0(1)
C(3)	24(1)	22(1)	31(1)	-1(1)	4(1)	0(1)
C(4)	26(1)	19(1)	31(1)	1(1)	6(1)	1(1)
C(6)	27(1)	19(1)	31(1)	0(1)	6(1)	1(1)
C(7)	28(1)	22(1)	33(1)	2(1)	2(1)	-1(1)
C(8)	22(1)	29(1)	39(1)	-1(1)	4(1)	0(1)
C(9)	23(1)	28(1)	38(1)	-1(1)	6(1)	0(1)
C(10)	30(1)	28(1)	35(1)	2(1)	6(1)	-1(1)
C(11)	38(1)	35(1)	35(1)	-2(1)	12(1)	0(1)
C(12)	46(1)	69(1)	51(1)	2(1)	20(1)	20(1)
C(13)	63(1)	57(1)	52(1)	-22(1)	20(1)	-15(1)
C(14)	69(1)	52(1)	43(1)	2(1)	25(1)	-2(1)
C(15)	29(1)	48(1)	43(1)	-13(1)	7(1)	2(1)
C(16)	26(1)	24(1)	38(1)	2(1)	6(1)	2(1)
C(17)	30(1)	22(1)	46(1)	-2(1)	6(1)	0(1)
C(18)	34(1)	28(1)	50(1)	-2(1)	1(1)	-5(1)
C(19)	40(1)	26(1)	57(1)	6(1)	6(1)	0(1)
C(20)	35(1)	28(1)	65(1)	-11(1)	11(1)	1(1)
C(21)	25(1)	22(1)	33(1)	-1(1)	6(1)	-1(1)
C(22)	28(1)	24(1)	34(1)	0(1)	2(1)	1(1)
C(23)	30(1)	25(1)	32(1)	-3(1)	2(1)	-2(1)
C(24)	30(1)	22(1)	34(1)	0(1)	6(1)	0(1)
C(25)	31(1)	24(1)	35(1)	2(1)	1(1)	2(1)
C(26)	29(1)	24(1)	33(1)	-1(1)	1(1)	-1(1)
C(27)	43(1)	22(1)	37(1)	-1(1)	2(1)	1(1)
C(28A)	22(1)	24(1)	40(1)	2(1)	5(1)	2(1)
C(29A)	29(1)	40(2)	37(1)	3(1)	7(1)	4(1)
C(30A)	28(1)	52(2)	45(1)	13(1)	4(1)	6(2)
C(31A)	25(1)	35(2)	66(1)	9(1)	7(1)	6(2)
C(32A)	23(2)	32(1)	65(1)	-10(1)	6(1)	2(1)
C(33A)	26(2)	31(1)	45(1)	-6(1)	2(1)	3(1)
C(28B)	22(1)	24(1)	40(1)	2(1)	5(1)	2(1)
C(29B)	29(1)	40(2)	37(1)	3(1)	7(1)	4(1)
C(30B)	28(1)	52(2)	45(1)	13(1)	4(1)	6(2)
C(31B)	25(1)	35(2)	66(1)	9(1)	7(1)	6(2)
C(32B)	23(2)	32(1)	65(1)	-10(1)	6(1)	2(1)
C(33B)	26(2)	31(1)	45(1)	-6(1)	2(1)	3(1)

Table 33. Hydrogen coordinates (x 10^4) and isotropic displacement parameters ($Å^2$x 10^3) for **13**.

	x	y	z	U(eq)
H(8)	12699	3932	6772	36
H(12A)	5833	5743	8533	82
H(12B)	5715	4990	8014	82
H(12C)	6643	5824	7896	82
H(13A)	8227	5992	9187	85
H(13B)	8941	6072	8525	85
H(13C)	9555	5399	9055	85
H(14A)	6702	4701	9385	80
H(14B)	8062	4114	9292	80
H(14C)	6587	3943	8870	80
H(15A)	12438	3197	5735	60
H(15B)	11205	3637	5272	60
H(15C)	10911	2746	5612	60
H(18A)	9229	7766	7160	56
H(18B)	9609	6899	6807	56
H(18C)	8519	6884	7351	56
H(19A)	8395	8501	6143	61
H(19B)	7242	8054	5643	61
H(19C)	8830	7681	5747	61
H(20A)	6609	8339	6964	64
H(20B)	6084	7406	7151	64
H(20C)	5522	7820	6477	64
H(22)	5056	2789	5273	34
H(23)	5283	1320	5118	35
H(25)	8096	1170	6688	36
H(26)	7887	2639	6846	34
H(27A)	6888	-146	6205	51
H(27B)	7742	23	5590	51
H(27C)	6041	-66	5511	51
H(29A)	3391	5058	5225	42
H(30A)	1463	5993	4994	50
H(31A)	935	7028	5734	50
H(32A)	2160	7053	6749	48
H(33A)	4140	6149	6980	41
H(29B)	3462	5214	5197	42
H(30B)	1710	6270	4990	50
H(31B)	1259	7253	5779	50
H(32B)	2459	7157	6797	48
H(33B)	4155	6068	7026	41

Table 34. Torsion angles [°] for **13**.

O(1)-C(4)-C(6)-O(2)	-179.77(10)
O(1)-C(4)-C(6)-C(7)	-1.08(18)
O(2)-C(6)-C(7)-O(4)	1.57(17)
O(2)-C(6)-C(7)-C(8)	179.76(10)
O(2)-C(10)-C(11)-C(12)	60.77(15)
O(2)-C(10)-C(11)-C(13)	-58.91(16)
O(2)-C(10)-C(11)-C(14)	179.95(13)
O(3)-C(10)-C(11)-C(12)	-117.55(17)
O(3)-C(10)-C(11)-C(13)	122.78(17)
O(3)-C(10)-C(11)-C(14)	1.6(2)
O(4)-C(7)-C(8)-C(9)	177.00(13)
O(5)-C(16)-C(17)-C(18)	51.16(14)
O(5)-C(16)-C(17)-C(19)	171.94(10)
O(5)-C(16)-C(17)-C(20)	-68.84(14)
O(6)-C(16)-C(17)-C(18)	-129.34(15)
O(6)-C(16)-C(17)-C(19)	-8.56(19)
O(6)-C(16)-C(17)-C(20)	110.66(17)
N(1)-N(2)-C(3)-C(1)	-0.48(14)
N(1)-N(2)-C(3)-C(21)	177.12(11)
N(1)-C(28A)-C(29A)-C(30A)	-178(2)
N(1)-C(28A)-C(33A)-C(32A)	178(3)
N(1)-C(28B)-C(29B)-C(30B)	-176.1(7)
N(1)-C(28B)-C(33B)-C(32B)	174.8(11)
N(2)-N(1)-C(2)-O(5)	179.44(11)
N(2)-N(1)-C(2)-C(1)	-0.26(14)
N(2)-N(1)-C(28A)-C(29A)	-44(2)
N(2)-N(1)-C(28A)-C(33A)	138(2)
N(2)-N(1)-C(28B)-C(29B)	-47.5(8)
N(2)-N(1)-C(28B)-C(33B)	134.1(8)
N(2)-C(3)-C(21)-C(22)	36.03(17)
N(2)-C(3)-C(21)-C(26)	-142.08(12)
C(1)-C(3)-C(21)-C(22)	-146.85(13)
C(1)-C(3)-C(21)-C(26)	35.04(19)
C(1)-C(4)-C(6)-O(2)	0.35(18)
C(1)-C(4)-C(6)-C(7)	179.04(11)
C(2)-O(5)-C(16)-O(6)	1.24(17)
C(2)-O(5)-C(16)-C(17)	-179.22(10)
C(2)-N(1)-N(2)-C(3)	0.46(14)
C(2)-N(1)-C(28A)-C(29A)	129.6(13)
C(2)-N(1)-C(28A)-C(33A)	-48(3)
C(2)-N(1)-C(28B)-C(29B)	124.5(5)
C(2)-N(1)-C(28B)-C(33B)	-54.0(10)
C(2)-C(1)-C(3)-N(2)	0.33(14)
C(2)-C(1)-C(3)-C(21)	-176.97(12)
C(2)-C(1)-C(4)-O(1)	-129.45(13)
C(2)-C(1)-C(4)-C(6)	50.44(19)
C(3)-C(1)-C(2)-O(5)	-179.71(12)
C(3)-C(1)-C(2)-N(1)	-0.03(14)
C(3)-C(1)-C(4)-O(1)	44.95(17)
C(3)-C(1)-C(4)-C(6)	-135.17(14)
C(3)-C(21)-C(22)-C(23)	-177.67(12)
C(3)-C(21)-C(26)-C(25)	177.48(12)
C(4)-O(1)-C(9)-C(8)	-1.22(18)
C(4)-O(1)-C(9)-C(15)	179.00(11)

C(4)-C(1)-C(2)-O(5)	-4.1(2)
C(4)-C(1)-C(2)-N(1)	175.54(11)
C(4)-C(1)-C(3)-N(2)	-175.02(12)
C(4)-C(1)-C(3)-C(21)	7.7(2)
C(4)-C(6)-C(7)-O(4)	-177.14(12)
C(4)-C(6)-C(7)-C(8)	1.05(17)
C(6)-O(2)-C(10)-O(3)	-2.56(18)
C(6)-O(2)-C(10)-C(11)	179.04(11)
C(6)-C(7)-C(8)-C(9)	-1.15(18)
C(7)-C(8)-C(9)-O(1)	1.3(2)
C(7)-C(8)-C(9)-C(15)	-178.95(13)
C(9)-O(1)-C(4)-C(1)	-179.01(10)
C(9)-O(1)-C(4)-C(6)	1.09(17)
C(10)-O(2)-C(6)-C(4)	92.43(13)
C(10)-O(2)-C(6)-C(7)	-86.32(13)
C(16)-O(5)-C(2)-N(1)	-78.90(15)
C(16)-O(5)-C(2)-C(1)	100.73(15)
C(21)-C(22)-C(23)-C(24)	-0.31(19)
C(22)-C(21)-C(26)-C(25)	-0.64(19)
C(22)-C(23)-C(24)-C(25)	0.30(19)
C(22)-C(23)-C(24)-C(27)	179.37(12)
C(23)-C(24)-C(25)-C(26)	-0.46(19)
C(24)-C(25)-C(26)-C(21)	0.6(2)
C(26)-C(21)-C(22)-C(23)	0.47(19)
C(27)-C(24)-C(25)-C(26)	-179.55(12)
C(28A)-N(1)-N(2)-C(3)	175.6(13)
C(28A)-N(1)-C(2)-O(5)	5.0(15)
C(28A)-N(1)-C(2)-C(1)	-174.7(14)
C(28A)-C(29A)-C(30A)-C(31A)	1.0(15)
C(29A)-C(28A)-C(33A)-C(32A)	1(4)
C(29A)-C(30A)-C(31A)-C(32A)	-3(2)
C(30A)-C(31A)-C(32A)-C(33A)	4(3)
C(31A)-C(32A)-C(33A)-C(28A)	-3(5)
C(33A)-C(28A)-C(29A)-C(30A)	0(2)
C(28B)-N(1)-N(2)-C(3)	173.5(4)
C(28B)-N(1)-C(2)-O(5)	6.8(5)
C(28B)-N(1)-C(2)-C(1)	-172.9(4)
C(28B)-C(29B)-C(30B)-C(31B)	0.5(4)
C(29B)-C(28B)-C(33B)-C(32B)	-3.6(14)
C(29B)-C(30B)-C(31B)-C(32B)	-1.9(5)
C(30B)-C(31B)-C(32B)-C(33B)	0.5(9)
C(31B)-C(32B)-C(33B)-C(28B)	2.1(14)
C(33B)-C(28B)-C(29B)-C(30B)	2.2(8)
